# The Relationship between Air Pollution Exposure, Genetic Liability for Schizophrenia, and Brain Outcomes in the ABCD Study

**DOI:** 10.1093/schbul/sbag138

**Published:** 2026-10-07

**Authors:** Giulia Cattarinussi, Yingzhe Zhang, Fabio Sambataro, Divyangana Rakesh, Paola Dazzan

**Affiliations:** Department of Psychological Medicine, Institute of Psychiatry, Psychology & Neuroscience, King's College London, London, SE5 8AB, United Kingdom; Department of Neuroscience (DNS), Padua Neuroscience Center, University of Padova, Padua, 35128, Italy; Department of Epidemiology, Harvard T.H. Chan School of Public Health, Boston, MA 02115, United States; Department of Neuroscience (DNS), Padua Neuroscience Center, University of Padova, Padua, 35128, Italy; Neuroimaging Department, Institute of Psychology, Psychiatry & Neuroscience, King's College London, London, SE5 8AB, United Kingdom; Department of Psychological Medicine, Institute of Psychiatry, Psychology & Neuroscience, King's College London, London, SE5 8AB, United Kingdom; National Institute for Health Research (NIHR) Mental Health Biomedical Research Centre at South London and Maudsley NHS Foundation Trust and King's College London, London, SE5 8AF, United Kingdom

**Keywords:** air pollution, fMRI, genetic liability, MRI, neurodevelopment, polygenic risk score

## Abstract

**Background and Hypothesis:**

Air pollution exposure has been associated with altered neurodevelopment and increased risk of schizophrenia. Individuals at high genetic risk may be more vulnerable to these effects. Here, we examined how air pollution relates to brain structure and functional connectivity in adolescents, and we tested for the first time whether genetic risk for schizophrenia exacerbates these effects.

**Study Design:**

We included n = 1580 individuals with information on genetic risk for schizophrenia, air pollution at 9-10 years, and structural and resting-state functional magnetic resonance imaging at 13-14 years from the Adolescent Brain Cognitive Development Study. We used linear mixed-effects models to examine the association between air pollution (ambient particulate matter (PM₂._5_), nitrogen dioxide (NO₂), and ozone (O₃)) and brain measures, and test whether genetic risk for schizophrenia moderated these effects.

**Study Results:**

Higher PM₂._5_ was associated with smaller right frontal pole surface area (p-FDR = 0.039). Individuals with low polygenic risk scores for schizophrenia (PRS-SCZ) and medium PM₂._5_ had smaller left caudate volumes compared with those with medium PRS-SCZ and medium PM₂._5_ (p-FDR = 0.019). In individuals with low PRS-SCZ, higher PM₂._5_ was associated with larger left (p-FDR = 0.019) and right (p-FDR = 0.018) caudate volumes. Higher NO₂ was associated with stronger negative functional connectivity between default mode and dorsal attention networks (p-FDR = 0.034) and weaker positive functional connectivity between dorsal attention and frontoparietal networks (p-FDR = 0.034).

**Conclusions:**

Air pollution and its interplay with genetic risk for schizophrenia are associated with structural and functional changes in areas implicated in schizophrenia pathophysiology. This underscores the potential of lowering air pollution during critical periods of neurodevelopment and informing proactive environmental policies.

## Introduction

Air pollution has been ranked by the World Health Organization (WHO) as the single greatest environmental threat to human health.^1^ In addition to the risk of respiratory, cardiovascular, and neoplastic disorders, exposure to air pollution also increases the risk for mental health problems.^2^ With air pollution affecting populations worldwide, we urgently need to understand the neurobiological mechanisms by which air pollution exposure in early life contributes to mental health disorders.

Children and adolescents are particularly vulnerable to the adverse effects of air pollution, due to their higher breathing rate to body size ratio, the immaturity of their immune system, the higher permeability of the blood–brain barrier, and the longer periods spent outdoors.^3^ Given that childhood and adolescence are sensitive periods of neurodevelopment, air pollution exposure during this time may significantly influence long-term cognitive and emotional functioning.^4^^,^^5^ Several studies have found a link between air pollution exposure and the presence of psychotic experiences in childhood and adolescence^6^^,^^7^; also, exposure to high air pollution levels in childhood has also been associated with a greater risk of developing schizophrenia in adulthood.^8^^,^^9^ This relationship, however, is unlikely to be explained only by environmental factors. For example, emerging evidence suggests that the effects of air pollution may interact with genetic vulnerability to jointly shape neurodevelopment.^10^^,^^11^ In support of this, a recent study found an association between long-term air pollution exposure and higher risk of developing schizophrenia. In this sample, the highest risk was observed in individuals with high polygenic risk scores (PRS) for schizophrenia and exposure to high air pollution levels.^12^

An important challenge in interpreting findings on the association between air pollution and mental health outcomes is ruling out the confounding effect attributable to other environmental exposures, such as disadvantageous neighborhood characteristics, including socioeconomic deprivation and urban living conditions. Indeed, socioeconomic deprivation, a well-established risk factor for mental health problems,^13^^,^^14^ is in itself associated with a higher probability of exposure to air pollution.^15^ However, recent studies indicate that the association between air pollution and mental health outcomes persists even after rigorous adjustment for socioeconomic status, suggesting that this association cannot be fully explained by socioeconomic confounding alone.^16–18^ Moreover, this epidemiological evidence is corroborated by findings from animal studies that have shown that exposure to real-world levels of air pollution during gestation and in the early postnatal period modulates neurodevelopment.^19^^,^^20^

The association between air pollution and increased risk of developing schizophrenia could be at least partially explained by the neurotoxic effects of air pollution exposure during childhood. Indeed, air pollution has been linked to changes in brain structure and functional connectivity mainly in frontal, parietal, temporal, cingulate, and striatal areas.^21^^,^^22^ Previous analyses of the Adolescent Brain Cognitive Development (ABCD) Study data have shown that air pollution exposure in childhood was associated with lower subcortical gray matter microarchitecture integrity^23^ and altered gray matter volumes in widespread cortical and subcortical areas in children aged 9-10 years.^24^ Of note, alterations in the same brain regions and networks have also been reported in individuals with psychotic experiences,^25^ schizophrenia,^26^ and in association with genetic risk for schizophrenia.^27^^,^^28^ Although both childhood air pollution exposure and genetic risk for schizophrenia have been found to be associated with changes in brain regions and networks implicated in psychosis, it remains unclear how these factors interact to shape neurodevelopment in ways that may contribute to increase the risk for schizophrenia. Here, we address this gap by testing for the first time how air pollution exposure and genetic risk of schizophrenia jointly influence brain structure and function in adolescents.

We used data from the ABCD Study to investigate whether childhood exposure to air pollutants (age 9-10 years), including particulate matter (PM) with diameter < 2.5 μm (PM₂._5_), nitrogen dioxide (NO₂), and ozone (O₃), interacts with genetic risk for schizophrenia to result in changes in brain structure and function in adolescence (age 13-14 years). We selected PM₂._5_ as the primary exposure of interest as this contributes substantially to the health burden of air pollution, given its small particle size that enables deep penetration into the respiratory system.^29^ NO₂ and O₃ were examined as secondary exposures because they capture complementary aspects of air pollution (i.e., traffic-related emissions and photochemical oxidants^30^) and may influence brain development through partially distinct biological pathways. We focused on air pollution exposure in childhood and brain changes in adolescence because these represent sensitive developmental windows that may be particularly vulnerable to the effects of air pollution, and because adolescence is a period of heightened risk for the onset of mental health problems. Based on previous findings,^21^^,^^22^ we used a region-of-interest approach focusing on brain areas that are both sensitive to air pollution and critical for cognitive and emotional processes, including frontal, parietal, temporal, and subcortical regions. We examined the relationship between air pollution and brain structure and function, and investigated whether genetic risk for schizophrenia moderates this relationship. In addition, we conducted exploratory analyses to examine whether air pollution exposure, genetic risk for schizophrenia, and the brain measures identified are associated with mental health measures at age 13-14 years, focusing on internalizing symptoms, externalizing symptoms, and psychotic-like experiences. We hypothesized that greater exposure to air pollution in childhood would be associated with lower volume, cortical thickness, and surface area, as well as reduced functional connectivity of regions and networks relevant to air pollution and the pathophysiology of schizophrenia during adolescence, and that this relationship would be moderated by higher genetic risk for schizophrenia. We focused our analyses on moderation models because studies of gene–environment interaction suffer from low power to detect significant effects and have a high likelihood of spurious findings.^31^^,^^32^

## Methods

### Participants

We used data from the ABCD Study (https://abcdstudy.org/, data release 5.1), a longitudinal study on children recruited between the ages of 9 and 10 years from 21 sites across the United States from 2016 to 2018. The ABCD Study includes sociodemographic data, including age, gender, race, socio-economic status, cognitive performance, mental and physical health assessments, structural and functional brain magnetic resonance imaging (MRI) scans, as well as measures of air pollution exposure (see Supplementary Material). In this study, we used data on air pollution exposure and genetic risk at baseline (T1, n = 11875 individuals aged 9-10 years) and on neuroimaging from the 4-year follow-up (T4, n = 11880 individuals aged 13-14 years). After excluding individuals due to missing data on air pollution exposure and genetic risk for schizophrenia (PRS-SCZ) at baseline and imaging quality control criteria at the T4 timepoint, a total of n = 1580 individuals were included in the final sample for the main analysis (see Supplementary Material). The mean (SD) age of the sample at the T4 timepoint included in the current study was 14.13 years (0.70). The sample was composed of 54.6% males. The majority (96.7%) were Non-Hispanic White, 1% were Hispanic, and 2.3% were Multi-Racial/Other. Sociodemographic characteristics for the final study sample can be found in Table 1.

**Table 1:** Demographic and socioeconomic characteristics.

	Study sample (n = 1580)
Age, years m (SD)	14.13 (0.7)
Sex n (%)	
Male	863 (54.6)
Female	717 (45.38)
Race/Ethnicity n (%)	
Non-Hispanic White	1527 (96.65)
Non-Hispanic Black	NA
Hispanic	16 (1.01)
Non-Hispanic Asian	NA
Multi-Racial/Other	37 (2.34)
Highest Parental Education n (%)	
Postgraduate degree Bachelor’s degree	712 (45.06) 520 (32.91)
Some college	297 (18.80)
High school/GED	44 (2.78)
Less than high school	7 (0.44)
Combined Household Income n (%)	
Low income	830 (52.53)
Middle income	494 (31.27)
High income	193 (12.22)
NA	63 (3.99)
Handedness n (%)	
Right	1307 (82.72)
Left	122 (7.72)
Mixed	151 (9.56)
MRI Manufacturer n (%)	
GE Medical Systems	408 (25.82)
Philips Medical Systems	150 (9.49)
Siemens	1022 (64.68)
Framewise displacement (mm) at age 13-14	0.11 (0.10)
2016 annual PM**₂._5_**: concentration (lg/m^3^) at age 9-10	7.42 (1.40)
2016 annual NO₂ concentration (ppb) at age 9-10	19.14 (6.16)
2016 annual O₃ concentration (ppb) at age 9-10	40.95 (4.35)
PRS-SCZ n (%)	
Low risk	525 (33.23)
Medium risk	543 (34.37)
High risk	512 (32.41)

Abbreviations: GED, General Educational Development; GE, General Electrics; MRI, magnetic resonance imaging; NA, not applicable; NO₂, Nitrogen dioxide; O₃, ozone; PM**₂._5_**, particulate matter ≤2.5 μm; PRS-SCZ, polygenic risk score of schizophrenia; SD, standard deviation.

### MRI Acquisition and Preprocessing

Imaging data at age 13-14 years (T4) were analysed. Imaging acquisition, processing, and harmonization have been extensively described elsewhere.^33^ Briefly, MRI scans were acquired across sites using harmonized protocols, using either a 3T Siemens, Philips, or GE MRI scanner. A 3-D T1-weighted image with 1-mm voxel resolution was acquired for each participant. Individuals also completed four 5-min resting-state scans to ensure at least 8 min of relatively low-motion data. Cortical surface reconstruction (using the Desikan-Killiany atlas) and subcortical segmentation (using the aseg atlas) were completed using FreeSurfer 7.1, including total gray and white matter, as well as subcortical volumes, cortical thickness, and cortical surface area estimates for cortical regions.^34^^,^^35^ Based on previous findings^21^^,^^22^ showing an effect of air pollution on frontal, parietal, temporal, and subcortical areas, we examined cortical volume, cortical thickness, and surface area (53 measures each, corresponding to 50 cortical regions of interest (ROIs) as well as global cortical measures computed for the left hemisphere, right hemisphere, and the whole cortex) and subcortical volume (14 measures) as regions of interest (Table S1). Given prior evidence of hemisphere-specific differences in brain structure related to air pollution exposure,^24^^,^^36^ we conducted separate analyses for the left and right hemispheres. Functional MRI data were preprocessed using the ABCD Data Analysis and Informatics Core’s standardized pipeline.^34^ Briefly, preprocessing included demeaning and detrending, followed by denoising using a general linear model incorporating nuisance regressors for head motion, white matter, cerebrospinal fluid, and the global signal, and subsequent band-pass filtering (0.008-0.09 Hz). Time courses were extracted from cortical surface-based ROIs using FreeSurfer’s functionally defined parcellation derived from functional connectivity patterns. Correlation values for each pair of ROIs within- or between-network, calculated on the basis of the Gordon parcellation scheme,^37^ were then Fisher z-transformed and averaged.^34^ We included between-network functional connectivity of the following networks: CON=Cingulo-Opercular Network, CPN=Cingulo Parietal Network, DAN=Dorsal Attention Network, DMN=Default Mode Network, FPN=Frontoparietal Network, SN=Salience Network, and VAN=Ventral Attention Network (Table S1). We selected these networks because of their central role in cognitive and emotional processes, their implication in the pathophysiology of schizophrenia and prior studies reporting connectivity alterations within and between these circuits in relation to air pollution exposure.^36^^,^^38^ Scans that did not meet quality control criteria recommended by the ABCD Data Analysis, Informatics & Resource Center (DAIRC; see release notes) were excluded. See Supplementary Material for details.

### Air Pollution Exposure Measures

Details on the collection of residential addresses and linkage to 1-year annual average ambient PM₂._5_, NO₂, and O₃ have been previously described.^39^ Briefly, the primary address at the baseline study visit was geocoded by the DAIRC using the Google Maps API to generate latitude and longitude (Google Maps Platform Documentation, 2019). Daily concentrations of PM₂._5_, NO₂, and 8-h maximum ground-level O₃ were estimated at a 1-km^2^ resolution across the United States using hybrid spatiotemporal models that integrate satellite-based aerosol optical depth, land-use regression, and chemical transport models.^40^ Their estimates were then averaged over the 2016 calendar year, when the children were aged 9-10 years and assigned to the geocoded primary residential address at the baseline ABCD study visit. The 2016 calendar year was chosen to correspond with the onset of enrolment in the ABCD study. See Supplementary Material for details.

### Computation of the Polygenic Risk Score of Schizophrenia

Detailed information about genotyping in ABCD has been previously described.^41^^,^^42^ Briefly, we extracted 7 989 528 genetic variants with minor allele frequency > 0.01, SNP-level call rate > 0.98, and Hardy–Weinberg equilibrium p > 1e–10 based on the imputed genotyping data. We identified adolescents of European ancestry using the first 6 principal components with random forest models and unrelated individuals based on PC-AiR.^41^ After removing 36 adolescents with principal components outliers within the European ancestry population, we retained 4470 unrelated adolescents of European ancestry. We calculated a PRS-SCZ based on a genome-wide association study conducted in individuals of European ancestry, including 53 386 cases and 77 258 controls.^43^ Although these genome-wide association study (GWAS) summary statistics are based on adults, previous research suggests a robust overlap between salient genetic factors in adolescents and adults.^44^ The single nucleotide polymorphism (SNP) weights for PRS construction were computed using PRS-CS software, a Bayesian scoring method which places a continuous shrinkage prior on SNP effect sizes.^45^ European samples from the 1000 Genomes Project Phase 3v5 reference panel were used to model linkage disequilibrium among variants. We then used PLINK to calculate polygenic scores for individuals in the target ABCD sample by summing all included variants weighted by the inferred posterior effect size for the effect allele. Individuals were categorized into three groups: low risk for schizophrenia (lowest tertile), medium risk for schizophrenia (middle tertile), or high risk for schizophrenia (highest tertile). This was done in line with previous studies to avoid imposing a linearity assumption on the association between genetic liability, air pollution measures and outcomes.^12^ Of note, PRS-SCZ was not correlated with air pollution measures (see Supplementary Material).

### Mental Health Measures

We used youth-reported internalizing and externalizing symptoms from the Brief Problem Monitor (BPM).^46^ Participants also completed the Prodromal Questionnaire–Brief Child Version (PQ-BC), a 21-item measure validated in children using the ABCD Study sample,^47^ assessing the presence of psychotic-like experiences over the past month. The total severity score was used as the measure of psychotic-like experiences.

### Statistical Analysis

We used linear mixed-effects models to examine the association between childhood annual PM₂._5_, NO₂, and O₃ exposure (included in the same model simultaneously as predictors) and adolescent brain measures (the outcome). In additional analyses, we modeled the interaction between PM₂._5_, NO₂, and O₃ exposure and PRS-SCZ as the predictor (each interaction was modeled separately). Based on previous literature,^12^ the annual average concentrations of air pollutants for each participant were classified into tertiles (low, intermediate, and high) to account for potential non-linear effects of air pollution on brain measures. Using these categories, we modeled an interaction term of air pollution (tertiles) and genetic risk (low, medium, and high) in the models (Table S2). We included a random effect for ABCD site and family within ABCD site for participant to account for clustered data. In exploratory analyses, we further used linear mixed-effects models to examine the associations between air pollution exposure, genetic risk for schizophrenia, and the brain measures identified with internalizing symptoms, externalizing symptoms, and psychotic-like experiences at age 13-14 years. Effect sizes were estimated using partial R^2^ to quantify the proportion of variance uniquely explained by each predictor in the models. Analyses were corrected for multiple comparisons using a false discovery rate (FDR) of *P* < .05 using the Benjamini-Hochberg method. Corrections were performed separately within each modality, including cortical volume, cortical thickness, and surface area (n = 53 comparisons for each modality), functional connectivity (n = 28 comparisons), and subcortical volume (n = 14 comparisons). PM₂._5_ was defined a priori as the primary exposure of interest, and all analyses were corrected for multiple comparisons using FDR without incorporating an additional correction factor for testing multiple pollutants. Analyses of NO₂ and O₃ were treated as secondary and were also FDR-corrected within their respective test sets. Models were conducted in three steps: unadjusted, adjusted for covariates of interest (baseline age, sex at birth, familial relationships, race and ethnicity, highest household education, total combined family income, child handedness, MRI manufacturer, and ABCD site), and fully adjusted for covariates of interest and multiple pollutants (PM₂._5_, NO₂, and O₃) (see Supplementary Material). Here, we report only the findings for the fully adjusted analyses. To ensure the robustness of our findings, we conducted sensitivity analyses to assess additional confounding effects of population density and distance to major roads, as well as of neighborhood deprivation (operationalized as area-deprivation index). In addition, we performed stratified analyses across low-, middle-, and high-income groups to examine whether associations were consistent across socioeconomic groups and across scanners. Lastly, considering that PRS-SCZ was calculated based on a genome-wide association study of European ancestry, we repeated the analyses only in the group of European ancestry. All model results can be found in the Supplementary Material. Analyses were performed using R (version 4.4.2).

## Results

### Association between PM₂._5_ and Brain Measures

Higher PM₂._5_ levels were significantly associated with a smaller surface area of the right frontal pole (F = 7.467, p-FDR = 0.040, partial R^2^ = 0.071) (Table S6). Specifically, individuals in the low PM₂._5_ tertile showed greater cortical surface area compared to those in the high PM₂._5_ tertile (p-FDR = 0.011). Similarly, individuals in the medium PM₂._5_ tertile had significantly larger cortical surface area relative to those in the high group (p-FDR = 0.004) (Figure 1, Table S7), suggesting that elevated PM₂._5_ levels may be associated with lower frontal surface area. Levels of PM₂._5_ were not associated with brain functional connectivity.

**Figure 1:**
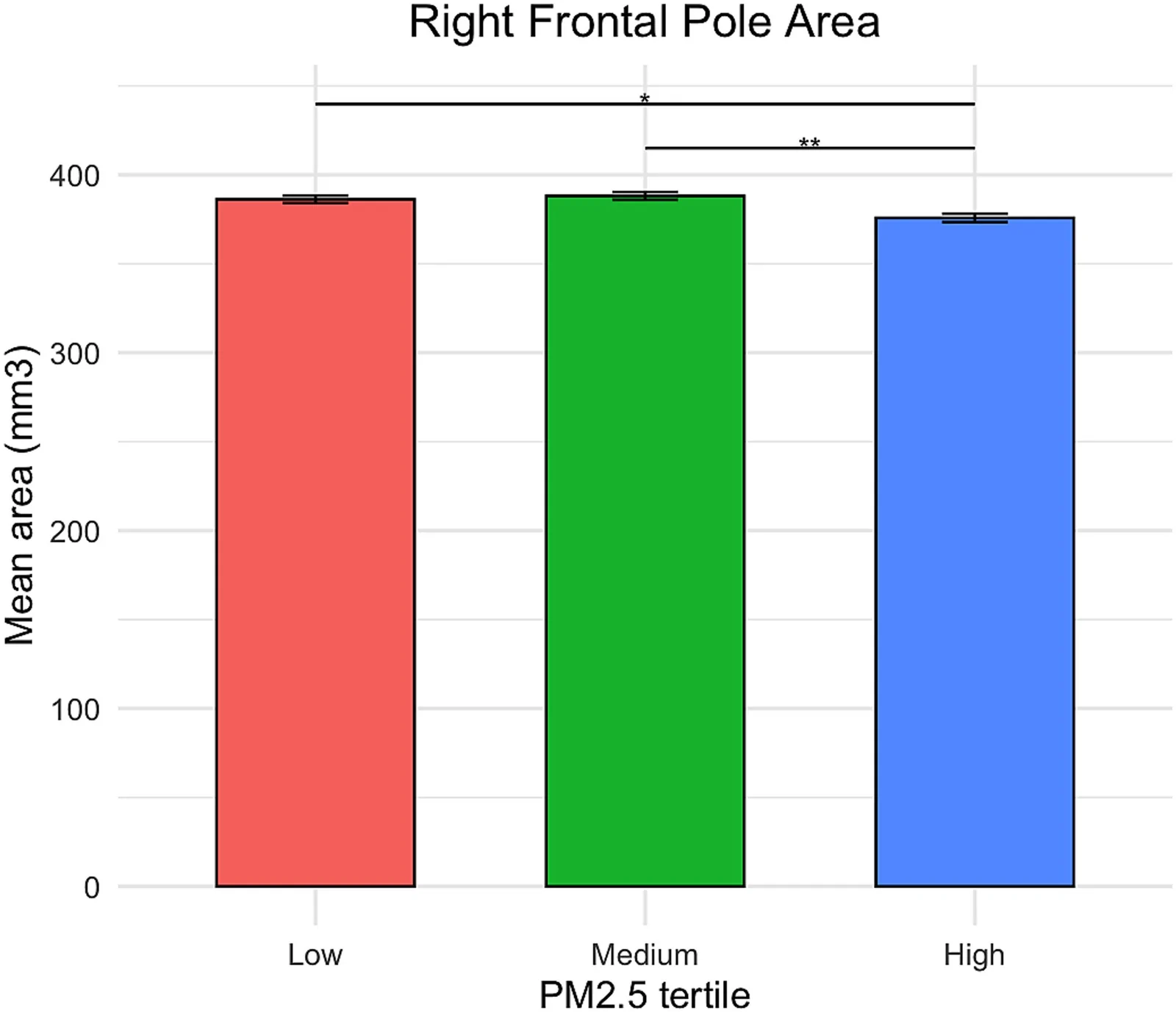
Association between PM₂._5_ Exposure and Right Frontal Pole Cortical Area (Fully Adjusted Model). Differences in Right Frontal Pole Cortical Area (mm^2^) across PM₂._5_ Exposure Tertiles. Points represent Estimated Differences between Tertiles, with 95% Confidence Intervals Shown as Error Bars. Statistical Significance Is Indicated as Follows: ^**^ P ≤ .01, ^*^ P ≤ .05. Graphs Were Generated Using *ggplot2* in R.

### Association between PM₂._5_, Genetic Risk for Schizophrenia, and Brain Measures

We found that PRS-SCZ significantly moderated the association between PM₂._5_ tertiles and left and right caudate volumes (F = 4.629, p-FDR = 0.014, partial R^2^ = 0.012 and F = 3.858, p-FDR = 0.028, partial R^2^ = 0.010; respectively) (Table 2). Specifically, individuals with low PRS-SCZ and medium PM₂._5_ exposure had significantly smaller left caudate volumes compared with individuals in the medium PRS-SCZ and medium PM₂._5_ exposure groups (p-FDR = 0.019). This suggests that genetic risk for schizophrenia modulates the association between PM₂._5_ exposure and striatal volume. Furthermore, in the low PRS-SCZ group, individuals exposed to high PM₂._5_ levels had a larger left caudate volume than those exposed to medium PM₂._5_ (p-FDR = 0.019) (Figure 2a, Table S8). For the right caudate, individuals in the low PRS-SCZ and high PM₂._5_ exposure group had significantly greater volume compared to the low PRS-SCZ and medium PM₂._5_ exposure group (p-FDR = 0.018) (Figure 2b, Table S9). No other comparisons were significant. There was no effect of PRS-SCZ or PM₂._5_ on caudate volume (p’s > 0.1, Tables S10 and S11). The results remained significant among participants of European ancestry (Table S12). This indicates that higher PM₂._5_ exposure may differentially affect caudate volume depending on genetic risk for schizophrenia, highlighting a potential gene–environment interaction.

**Table 2:** Results from linear mixed-effects models testing the interaction effects of PM₂._5_ tertile and polygenic risk score of schizophrenia tertile on subcortical volumes (fully adjusted model).

**Subcortical volume**	**term**	**F**	**p-FDR**
Volume in mm^3^ of left-thalamus-proper	PRS-SCZtert:PM₂._5_tert	1.180	0.617
Volume in mm^3^ of left-caudate	PRS-SCZtert:PM₂._5_tert	4.629	**0.014**
Volume in mm^3^ of left-putamen	PRS-SCZtert:PM₂._5_tert	2.026	0.248
Volume in mm^3^ of left-pallidum	PRS-SCZtert:PM₂._5_tert	1.104	0.617
Volume in mm^3^ of left-hippocampus	PRS-SCZtert:PM₂._5_tert	0.611	0.840
Volume in mm^3^ of left-amygdala	PRS-SCZtert:PM₂._5_tert	0.310	0.871
Volume in mm^3^ of left-accumbens-area	PRS-SCZtert:PM₂._5_tert	0.895	0.725
Volume in mm^3^ of right-thalamus-proper	PRS-SCZtert:PM₂._5_tert	2.056	0.248
Volume in mm^3^ of right-caudate	PRS-SCZtert:PM₂._5_tert	3.858	**0.028**
Volume in mm^3^ of right-putamen	PRS-SCZtert:PM₂._5_tert	2.914	0.095
Volume in mm^3^ of right -pallidum	PRS-SCZtert:PM₂._5_tert	0.522	0.840
Volume in mm^3^ of right -hippocampus	PRS-SCZtert:PM₂._5_tert	0.417	0.858
Volume in mm^3^ of right -amygdala	PRS-SCZtert:PM₂._5_tert	1.541	0.438
Volume in mm^3^ of right -accumbens-area	PRS-SCZtert:PM₂._5_tert	0.596	0.840

PM₂._5_tert: particulate matter ≤2.5 μm tertile; PRS-SCZtert: polygenic risk score of schizophrenia. Bold values indicate significance.

**Figure 2:**
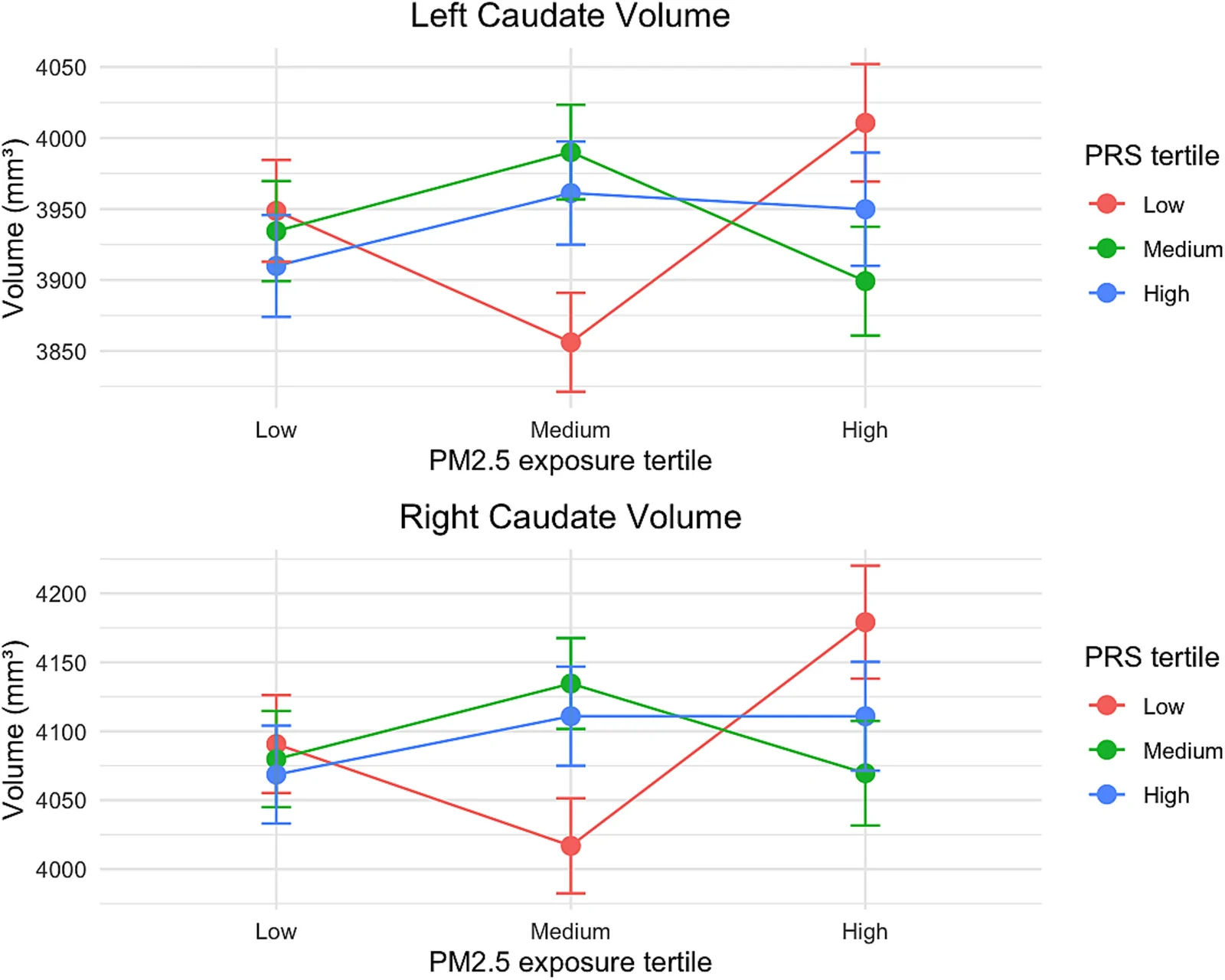
Association between PM₂._5_ Exposure, PRS-SCZ and Subcortical Brain Volumes. Estimated Volumes of the Left Caudate (A) and Right Caudate (B) across Tertiles of PM₂._5_ Exposure, Stratified by Polygenic Risk Score for Schizophrenia (PRS-SCZ). Points Represent Estimated Marginal Means with Standard Error Bars. Colors Indicate PRS-SCZ Tertiles. Graphs Were Generated Using *ggplot2* in R.

### Association between NO₂ and O₃ and Brain Measures

Levels of NO₂ and O₃ were not associated with any brain structural measure. In the fully adjusted model, including both covariates and other pollutants, NO₂ levels were significantly associated with DAN with the DMN (F = 5.541, p-FDR = 0.043, partial R^2^ = 0.036) and the FPN (F = 6.312, p-FDR = 0.043, partial R^2^ = 0.069). In the overall sample, connectivity between the DMN and the DAN was negative, consistent with their typical anti-correlated relationship. This negative connectivity was stronger (i.e., more negative) in individuals in the high NO₂ tertile compared to those in the low and medium NO₂ tertile (p-FDR = 0.002 and p-FDR = 0.017, respectively), suggesting that higher NO₂ levels are associated with greater anti-correlation between the DAN and DMN. In contrast, connectivity between the DAN and the FPN was positive. We observed greater positive connectivity between the DAN and the FPN in the low NO₂ tertile relative to both the medium and the high NO₂ tertile (p-FDR = 0.005 and p-FDR = 0.017, respectively), suggesting that higher NO₂ levels are linked to reduced positive DAN–FPN connectivity (see Supplementary Material). Levels of O₃ were not associated with brain functional connectivity.

### Association between NO₂ and O₃, Genetic Risk for Schizophrenia, and Brain Measures

There was no association between levels of NO₂ and O₃, PRS-SCZ, and brain measures.

### Sensitivity Analysis

Results remained significant in models that additionally adjusted for population density and distance to roads. Most results remained significant in models that additionally adjusted for area deprivation index (see Supplementary Material).

### Association of Air Pollution, Genetic Risk for Schizophrenia, and Brain Measures with Mental Health

Exploratory analyses showed that higher PM₂._5_ levels were significantly associated with higher psychotic-like experiences total severity score at age 13-14 years (F = 3.206, *P* = 0.046). Resting-state fMRI connectivity between the DAN and DMN was positively associated with internalizing (F = 6.450, *P* = 0.011) and externalizing (F = 4.903, *P* = 0.026) symptoms. All other tested associations were not statistically significant (Table S34).

## Discussion

To our knowledge, this is the first study to explore the interplay between air pollution exposure and genetic risk of schizophrenia in relation to brain structure and function in adolescents. We found that individuals with a low PRS-SCZ and medium PM₂._5_ exposure have significantly smaller caudate volumes compared to both individuals with medium genetic risk and medium PM₂._5_ exposure, and also to those with a similarly low PRS-SCZ but a high PM₂.5 exposure. This points to the presence of environment-dependent effects of genetic susceptibility on striatal volume. In addition, using a large adolescent sample, we show that exposure during childhood to PM₂._5_ is associated with lower right frontal pole area, while exposure to NO₂ is associated with altered functional connectivity of the DAN with the DMN and FPN in adolescents. This suggests that PM₂._5_ and NO₂ influence brain structure and function during sensitive periods of development.

Our study shows that exposure to PM₂._5_ is associated with changes in striatal volume that differ depending on genetic risk for schizophrenia. Importantly, we observe a differential association between genetic risk for schizophrenia and striatal volume depending on the level of PM₂._5_ exposure. Contrary to our hypothesis, this interaction was driven primarily by individuals with low genetic risk for schizophrenia, rather than those with higher genetic risk. In particular, low PRS-SCZ was associated with smaller left caudate volume, and this effect was strongest at medium PM₂._5_ exposure. This pattern suggests that in individuals with low genetic risk for schizophrenia, environmental exposure may have a more pronounced association with caudate development, as there is less underlying genetic risk influencing its volume. The absence of a similar effect in the *low PM₂._5_ exposure* group may reflect a threshold effect, where very low levels of PM₂._5_ exposure are not linked to structural changes. Interestingly, within the low PRS-SCZ group, individuals exposed to high PM₂._5_ exhibited larger bilateral caudate volume compared with those exposed to medium PM₂._5_. One possible explanation for this counterintuitive pattern is that neurodevelopmental variability associated with higher genetic risk for schizophrenia may reduce the relative contribution of environmental variation in PM₂._5_ exposure to striatal structure, thereby limiting our ability to detect additional effects. Alternatively, it may indicate that the present study design and effect sizes are insufficient to detect gene–environment interactions in these groups. Striatal structural alterations are particularly relevant to schizophrenia, given the striatum's central role in cognitive control, reward processing and goal-directed behaviors,^48^^,^^49^ domains frequently affected in psychiatric disorders.^50–52^ Changes in striatal volume during adolescence could therefore reflect early neurodevelopmental abnormalities that increase vulnerability to cognitive impairment and psychiatric symptoms later in life. The differential effects observed at moderate versus high PM.25 exposure levels highlight that environmental risk factors may interact with genetic background in complex, non-linear ways, modifying neurodevelopmental trajectories that contribute to schizophrenia risk. In addition, PM₂._5_ exposure levels were relatively low in this predominantly European ancestry sample, which may limit the generalisability of these findings, as these associations may differ across the broader sociodemographic and ethnic distribution where both exposure profiles and vulnerability are likely to vary substantially.

Consistent with our first hypothesis, greater exposure to PM₂._5_ in late childhood was associated with structural alterations in brain areas involved in the pathophysiology of psychiatric disorders. In particular, in line with previous investigations in a different subset of ABCD data,^24^ adolescents at ages 13-14 that had higher PM₂._5_ exposure at 9-10 years of age presented a smaller right frontal pole area compared to those with lower PM₂._5_ exposure. The frontal pole, which is comprised of the most rostral portions of the superior, middle, and inferior frontal gyri, is involved in several cognitive functions, including learning, problem solving, and decision making.^53^^,^^54^ We speculate that lower right frontal pole area in association to PM₂._5_ exposure may reflect air pollutants-induced changes in neurodevelopmental processes, such as neuronal proliferation and synaptic pruning.^24^^,^^55^ Compelling evidence suggests that frontal pole abnormalities are implicated in the pathophysiology of a number of psychiatric disorders, including major depressive disorder,^56^ bipolar disorder^57^ and schizophrenia,^58^ which suggests that exposure to PM₂._5_ during childhood may partially contribute to risk for these disorders through effects on brain maturation during adolescence. In line with this, exploratory analyses showed that higher PM₂._5_ levels were associated with higher psychotic-like experiences severity at age 13-14 years, which confirms previous findings.^18^ An important aspect to consider when interpreting our findings is that the average levels of PM₂._5_ exposure were relatively low compared to the other studies.^59–61^ Thus, it is possible there may be differential dose-dependent effects that warrant further investigation.

Notably, we found no associations between PM₂._5_ and cortical thickness or brain volume. Given that these measures reflect partially distinct maturational processes,^62^ our result could have neurobiological implications about developmental timing. Indeed, surface area expands much more dynamically than cortical thickness and largely drives the growth of the cerebral cortex during the perinatal and early postnatal stages.^63^ In addition, timing of development of surface areas and cortical thickness varies over the cortex, with primary sensory regions reaching their peak earlier in development, followed by later maturation of higher-order association areas in parietal and prefrontal regions.^62^ The selective association with surface area therefore raises the possibility that PM₂._5_ exposure may be more closely related to earlier phases of cortical development, with limited impact on later maturational processes indexed by cortical thickness and brain volume.

In a secondary analysis, we observed that high NO₂ exposure at 9-10 years of age was associated with stronger negative connectivity between the DMN and DAN and weaker positive connectivity between the DAN and FPN in adolescents at ages 13-14. A recent systematic review highlighted a significant association between air pollution exposure and abnormal functional connectivity between large-scale brain networks during childhood.^64^ Our findings extend previous work by providing prospective evidence that childhood exposure to air pollution is linked to altered functional connectivity in early adolescence. DAN, DMN, and FPN play a key role in visuospatial attention, executive function, and self-referential thoughts.^65–67^ Alterations in the interplay between these networks have commonly been reported in adolescents and adults with psychiatric disorders, including major depressive disorder, and schizophrenia^68–71^ and have also been linked to transdiagnostic dimensions of psychopathology.^69^ Our finding of stronger negative connectivity between the DAN and the DMN may suggest less effective communication between these two networks, which may result in difficulties in switching between externally focused and internally focused stimuli, ultimately leading to impairments in goal-directed control of attention, emotion, and self-referential thought.^72^ Differently, weaker connectivity between the DAN and the FPN may indicate reduced top-down cognitive control,^73^ possibly affecting attention and executive function. We could therefore speculate that childhood exposure to NO₂ could contribute to changes in brain networks underlying cognitive and emotional processing in adolescents. Of note, global signal regression was applied as part of the preprocessing pipeline, which may have introduced “artefactual” anti-correlations.^74^ However, the negative connectivity between the DMN and the DAN was consistent with their well-established intrinsic anticorrelated relationship. Importantly, these effects were observed both in the adjusted and fully adjusted models that also considered the confounding effect of the other air pollutants. This suggests that NO₂ and PM₂._5_ may exert independent effects on neurodevelopment, rather than only acting in association with other air pollutants. These findings highlight the possibility that NO₂ and PM₂._5_ contribute to alterations in brain structure and function through partially distinct neurobiological mechanisms during senstivie periods of development. The lack of direct convergence between structural and functional findings is not unexpected, as structural and functional MRI measures capture different aspects of brain organization and may differ in their sensitivity to environmental exposures and genetic vulnerability.

Although air pollution exposure was associated with the surface area of the frontal pole and resting-state functional connectivity, there were no significant interaction effects with genetic risk for schizophrenia on these measures (see Supplementary Material). This is somewhat surprising, as we were expecting that genetic susceptibility for schizophrenia would exacerbate the impact of air pollution on these brain measures. Instead, our findings suggest that air pollution and genetic risk may act independently on surface area and resting-state functional connectivity, similar to observations reported for other well-established risk factors for schizophrenia, such as cannabis use and obstetric complications.^75^^,^^76^

The design of our study does not allow us to disentangle whether the observed associations reflect a direct biological effect of air pollution, indirect pathways, or simply co-occurrence driven by contextual confounding. Notably, the associations reported remained significant after accounting for key socioeconomic and environmental factors, supporting the robustness of the findings. In addition, our results align with animal studies showing that exposure to air pollution during gestation and early postnatal period modulates neurodevelopment.^19^^,^^20^ Nevertheless, although causal interpretations cannot be made, several biological mechanisms may be considered to speculatively explain the associations we observed. Air pollution is known to induce neuroinflammation, which can activate microglia and disrupt normal synaptic pruning during adolescence.^3^ Of note, systemic inflammation has been associated with changes in striatal volume,^77^ pointing to a potential pathway through which air pollution may affect striatal structure. In addition, air pollution can cause oxidative stress and result in changes in brain plasticity and progressive neurodegeneration.^78^ Furthermore, air pollutants may interfere with neurodevelopmental processes by damaging epithelial and endothelial barriers, including the blood–brain barrier.^79^ Additional studies are needed to elucidate the biological mechanisms underlying the association between air pollution exposure, genetic risk for schizophrenia and brain abnormalities.

The study has several strengths, including a large nation-wide cohort with air pollution estimates with high spatiotemporal precision and the ability to adjust for demographic and socio-economic confounders. However, some limitations should also be acknowledged. First, the observational nature of the study precludes causal inference and therefore the reported associations should be interpreted as correlational rather than indicative of direct neurobiological effects. Second, we used a single measure of the concentration of different air pollutants at baseline to quantify air pollution exposure. As measures of lifetime cumulative exposure are not available in the ABCD dataset, we were not able to assess the role of lifetime air pollution exposure on brain structure and function. In addition, the ABCD dataset does not provide detailed information on residential mobility across follow-up, preventing us from determining whether participants remained at the same residence between 9-10 and 13-14 years. We acknowledge that this limitation affects the temporal interpretation of the exposure measures, as the baseline air pollution estimates may reflect either a proxy for longer-term exposure in the case of residential stability, or a more temporally limited exposure measure in the case of residential mobility. Future studies that incorporate these measures will allow us to identify specific windows of susceptibility and the short- or long-term impact of air pollution on brain development. Third, although baseline imaging is available in the ABCD study, we chose not to incorporate these measures into the present study which focuses specifically on the association between childhood air pollution exposure and brain measures in adolescence. This analytical decision limits the interpretation of the findings, as it prevents us from determining whether the observed differences reflect altered developmental trajectories or pre-existing structural differences. Fourth, this study was restricted to individuals of European ancestry, which limits generalizability of the results. We did not include individuals of other ancestries because the adequately powered GWAS for PRS derivation remain largely limited to European-ancestry populations; and applying European-derived PRS weights to individuals of other ancestries yields unreliable estimates due to differences in linkage disequilibrium patterns and allele frequencies between populations.^80^ We acknowledge this as a limitation and encourage future studies leveraging larger non-European ancestry GWAS resources to address this gap. Furthermore, the dataset does not provide comprehensive measures of near-roadway pollutants (i.e., carbon monoxide, hydrocarbons).^81^ Although sensitivity analyses suggested that our findings were robust to adjustment for population density and distance to major roads, these measures do not fully capture traffic-related pollution. More refined exposure metrics, including near-roadway pollutants, will be necessary to disentangle their effects on brain development. Lastly, although we accounted for potential confounding due to site-level differences by adjusting for study site in all statistical models, heterogeneity across study sites in both air pollution exposures and socioeconomic indicators should be considered when interpreting the findings.

In conclusion, the results show that exposure to air pollution during childhood is associated with structural and functional changes in areas that are implicated in cognitive and emotional processing, even at levels of exposure below the current standard. In addition, the interaction between genetic risk for schizophrenia and moderate levels of PM₂._5_ exposure was associated with striatal volume, suggesting potential environment-dependent effects of genetic susceptibility on brain structure. Future research should build on these findings by employing longitudinal neuroimaging designs to better capture developmental trajectories, examining sensitive windows of exposure across early life, and integrating broader social and environmental risk factors (i.e., socioeconomic adversity, urbanicity, and co-exposures) to more comprehensively characterize how environmental influences shape brain structure and function in vulnerable populations. In addition, future work should examine whether air pollution exposure, genetic liability for schizophrenia, and any associated brain alterations are linked to behavioral and physical health outcomes. Integrating these approaches with genetic risk could inform precision psychiatry strategies aimed at preventing neurodevelopmental changes in children and adolescents.

## Supplementary Material

Supplementary_Material_sbag138
