# Supplementary material for: The Relationship between Air Pollution Exposure, Genetic Liability for Schizophrenia, and Brain Outcomes in the ABCD Study
Source: Schizophr Bull. 2026 Oct 7;52(5):sbag138. doi: 10.1093/schbul/sbag138 (PMC13644880; doi:10.1093/schbul/sbag138)
Supplement: Supplementary file 1 [file supplementary_material_sbag138.docx]

# **The relationship Between Air Pollution Exposure, Genetic Liability for Schizophrenia, and Brain Outcomes in the ABCD Study**

*Supplementary Material*

**Supplementary Methods**

**Supplementary Results**

**Supplementary Tables 1-34**

**Supplementary Figures 1-4**

**Supplementary Methods**

**Participants**

We used data from the ABCD Study (https://abcdstudy.org/), a longitudinal study on children recruited at the age of nine to ten from 21 sites across the United States from 2016 to 2018. The ABCD Study includes sociodemographic data, including age, gender, race, socio-economic status, cognitive performance, mental and physical health assessment, structural and functional brain MRI scans, as well as measures of exposure to air pollution. Centralized approval from the Institutional Review Board was obtained from the University of California, San Diego. Written informed consent from the parents and assent from the child were obtained for each participant. Data collection and analysis were carried out in strict compliance with ethical regulations. The ABCD dataset is provided by the National Institute of Mental Health Data Archive (NDA) (<https://nda.nih.gov/>). In this study, we used data on air pollution exposure and genetic risk at baseline (T1, n=11,875 individuals aged 9-10) and on neuroimaging from the 4-year follow-up (T4, n=11,880 individuals aged 13-14). After excluding individuals based on missing data on air pollution exposure and genetic risk for schizophrenia (PRS-SCZ) at baseline and imaging quality control criteria at the T4 timepoint, a total of n=1,580 individuals were included in the final sample for the main analysis (see Supplementary Material). The mean (SD) age of the sample at the T4 timepoint included in the current study was 14.13 years (0.70). The sample was composed of 54.6% males. The majority (96.7%) were Non-Hispanic White, 1 % were Hispanic (code: 3), and 2.3% were Multi-Racial/Other (code: 5). Sociodemographic characteristics for the final study sample can be found in Table 1.

**MRI acquisition and preprocessing**

Imaging data at age 13-14 years (T4) were analysed. Imaging acquisition, processing, and harmonization have been extensively described elsewhere ^1^. Briefly, MRI scans were acquired across sites using harmonized protocols, using either a 3T Siemens, Phillips, or GE MRI scanner. A 3-D T1-weighted image with 1-mm voxel resolution was acquired for each participant. Individuals also completed four 5-minute resting-state scans to ensure at least eight minutes of relatively low-motion data. Preprocessing was performed by the ABCD Data Analysis and Informatics Core using a standardized pipeline ^2^. Motion detection and correction programs were used in real time at the Siemens and GE sites ^3,4^. The quality of the data was checked with both manual and automatic techniques. All images were examined by trained professionals for artifacts and abnormalities. The signal-to-noise-ratio and head motion measurements were calculated using automated processes. Cortical surface reconstruction (using the Desikan-Killiany atlas) and subcortical segmentation (using the aseg atlas) were completed using FreeSurfer (version 5.3), including total gray and white matter, as well as subcortical volumes, cortical thickness, and cortical surface area estimates for cortical regions ^2,5^. Based on previous findings ^6,7^ showing an effect of air pollution on frontal, parietal, temporal, and subcortical areas, we examined cortical volume, cortical thickness, and surface area (53 measures each) and subcortical volume (14 variables) of regions of interest (see Supplementary Material). Given prior evidence of hemisphere-specific differences in brain structure related to air pollution exposure ^8,9^, we conducted separate analyses for the left and right hemispheres. For fMRI analyses, fMRI time courses were projected onto FreeSurfer’s cortical surface. Using these time courses, within- and between-network connectivity were calculated using the Gordon parcellation scheme ^10^ for 12 predefined resting state networks (i.e., AN=Auditory Network, CON=Cingulo Opercular Network, CPN=Cingulo Parietal Network, DAN=Dorsal Attention Network, [DMN](https://www.sciencedirect.com/topics/neuroscience/default-mode-network)=Default Mode Network, FPN=Frontoparietal Network, RTN=Retrosplenial Temporal Network, SMN (H)=Sensorimotor Network (hand), SMN (M)=Sensorimotor Network (mouth), SN, VAN, VN=Visual Network). Correlation values for each pair of ROIs within- or between-networks were then Fisher’s Z transformed ^2^. Within-network connectivity reflects the average of the correlation over all pairs of ROIs within a network, while between-network connectivity reflects the average correlation value between all ROIs of two separate networks. Our analyses focused on both the within- and between-network functional connectivity of the following networks: CON, CPN, DAN, [DMN](https://www.sciencedirect.com/topics/neuroscience/default-mode-network), FPN, SN, and VAN (Tab S.1). We selected these networks because of their central role in cognitive and emotional processes, their implication in the pathophysiology of schizophrenia and prior studies reporting connectivity alterations within and between these circuits in relation to air pollution exposure ^9,11^. Scans that did not meet quality control criteria recommended by the ABCD Data Analysis, Informatics & Resource Center (DAIRC; see release notes) were excluded.

**Air pollution exposure measures**

Details on the collection of residential addresses and linkage to one-year annual average ambient particulate matter (PM₂.₅), nitrogen dioxide (NO₂), and ozone (O₃) have been previously described ^12^. Briefly, the address at which participants spent most of their time was obtained from the participant’s caregiver during the baseline study visit and geocoded by the DAIRC using the Google map API to generate latitude and longitude (Google Maps Platform Documentation, 2019). Daily concentrations of PM₂.₅, NO₂, and 8-hour maximum ground-level O₃ were estimated at a 1-km² resolution across the United States using hybrid spatiotemporal models that integrate satellite-based aerosol optical depth, land-use regression, and chemical transport models. ^13^. Their estimates were then averaged over the 2016 calendar year, when the children were aged 9-10 years and assigned to the geocoded primary residential address at the baseline ABCD study visit. The 2016 calendar year was chosen to correspond with the onset of enrolment in the ABCD study. PM₂.₅ was reported in µg/m3, and O₃ and NO₂ were reported in parts per billion (ppb). Nationwide, the annual PM₂.₅, NO₂, and O₃ concentration estimates are highly accurate, with a cross-validated R2 of 0.89, 0.84, and 0.90, respectively ^12–14^. The mean (SD) annual pollutants concentrations from the 2016 calendar year were as follows: PM₂.₅: 7.42 (1.40) µg/m3, NO₂: 19.14 (6.16) ppb, and O₃: 40.95 (4.35) ppb. PM₂.₅ and NO₂ levels were below current Environmental Protection Agency guidelines (https://www.epa.gov/criteria-air-pollutants). PM₂.₅ was positively correlated with NO₂ (ρ=0.12, p < 0.001) and negatively correlated with O₃ (ρ=-0.26, p < 0.001). The correlation between NO₂ and O₃ was not significant (ρ=0.01, p=0.62). We used one-way analysis of variance (ANOVA) to assess differences in air pollutant levels across the 21 study sites. Mean concentrations differed significantly between sites for PM₂.₅ (F=111.1, p<0.001) NO₂ (F=107.1, p<0.001), and O₃ (F=61.35, p<0.001) (Tab. S.3). Variance inflation factor analyses indicated no evidence of multicollinearity among PM2.5, NO2, and O3 exposure variables (all VIFs=1.00).

**Covariates of interest**

Potential covariates were selected based on previous studies^11^. The following covariates were included in the main analysis: baseline age, sex at birth, familial relationships, race and ethnicity (Non-Hispanic White, Non-Hispanic Black, Hispanic, Non- Hispanic Asian or Multi-Racial/Other), highest household education, total combined family income (high income: $100K, middle income: $50K<$100K, and low income: <$50K), child handedness, MRI manufacturer (Siemens, Philips, GE), and ABCD site. The distribution of combined household income varied significantly across the 21 study sites (χ²=169.96, p<0.001; Table S.4). Similarly, the distribution of highest parental education differed significantly across sites (χ²= 223.21, p<0.001; Table S.5). For subcortical volume analyses, intracranial volume (ICV) was also included as a covariate. Since air pollution exposure is likely to be impacted by the urban built environment, we conducted sensitivity analyses to assess additional confounding effects of population density and proximity to major roads. Residentially derived United Nations population density was estimated as persons per km^2^ as a proxy for urbanicity.

**Supplementary Results**

**Association between air pollution exposure and genetic risk of schizophrenia**

There was no significant correlation between air pollution exposures and PRS-SCZ. All associations were small in magnitude and non-significant for PM₂.₅ (r=0.050, p-FDR=0.145), NO₂ (r=−0.035, p-FDR=0.248), and O₃ (r=0.004, p-FDR=0.868), indicating no gene–environment correlation in this sample.

**Association between air pollution exposure and brain measures (adjusted model)**

Results reported are from the adjusted models, including covariates. PM₂.₅ levels were significantly associated with the area of the right frontal pole (p-FDR = 0.050) (Tab. S.17). Specifically, individuals in the low PM₂.₅ tertile showed greater cortical area compared to those in the high PM₂.₅ tertile (p-FDR = 0.030). Similarly, individuals in the medium PM₂.₅ tertile had significantly larger cortical area relative to the high group (p-FDR = 0.003) (Fig. S.3, Tab. S.18). Also, nitrogen dioxide (NO₂) levels were significantly associated with the resting-state fMRI functional connectivity between the Dorsal Attention Network (DAN) and the Default Mode Network (DMN) (p-FDR = 0.056, trend level) and with the resting-state fMRI connectivity between the DAN and the Frontoparietal Network (FPN) (p-FDR = 0.049) (Tab. S.29). In particular, connectivity between the DAN and the DMN was significantly higher in the low NO₂ tertile compared to the high NO₂ tertile (p-FDR = 0.006). Similarly, there was a greater connectivity between the DAN and the FPN in the low NO₂ tertile relative to both the medium NO₂ tertile (p-FDR = 0.003) and the high NO₂ tertile (p-FDR = 0.012) (Fig. S.2, Tab. S.30). No effect of O₃ on brain measures was observed.

**Table S.1. NIMH National Data Archive MRI variable names and variable definitions of the included brain areas and networks.**

| **Measure** | **NDA Variable Name** | **NDA Variable Definition** |
| --- | --- | --- |
| **sMRI** | | |
| **Cortical**  **Thickness** | SMRI_THICK_CDK_BANKSSTSLH  SMRI_THICK_CDK_CDACATELH  SMRI_THICK_CDK_CDMDFRLH  SMRI_THICK_CDK_FUSIFORMLH  SMRI_THICK_CDK_IFPLLH  SMRI_THICK_CDK_IFTMLH  SMRI_THICK_CDK_IHCATELH  SMRI_THICK_CDK_LOBFRLH  SMRI_THICK_CDK_MOBFRLH  SMRI_THICK_CDK_MDTMLH  SMRI_THICK_CDK_PARAHPALLH  SMRI_THICK_CDK_PARSOPCLH  SMRI_THICK_CDK_PARSOBISLH  SMRI_THICK_CDK_PARSTGRISLH  SMRI_THICK_CDK_PTCATELH  SMRI_THICK_CDK_PCLH  SMRI_THICK_CDK_RRACATELH  SMRI_THICK_CDK_RRMDFRLH  SMRI_THICK_CDK_SUFRLH  SMRI_THICK_CDK_SUPLLH  SMRI_THICK_CDK_SUTMLH  SMRI_THICK_CDK_FRPOLELH  SMRI_THICK_CDK_TMPOLELH  SMRI_THICK_CDK_TRVTMLH  SMRI_THICK_CDK_INSULALH  SMRI_THICK_CDK_BANKSSTSRH  SMRI_THICK_CDK_CDACATERH  SMRI_THICK_CDK_CDMDFRRH  SMRI_THICK_CDK_FUSIFORMRH  SMRI_THICK_CDK_IFPLRH  SMRI_THICK_CDK_IFTMRH  SMRI_THICK_CDK_IHCATERH  SMRI_THICK_CDK_LOBFRRH  SMRI_THICK_CDK_MOBFRRH  SMRI_THICK_CDK_MDTMRH  SMRI_THICK_CDK_PARAHPALRH  SMRI_THICK_CDK_PARSOPCRH  SMRI_THICK_CDK_PARSOBISRH  SMRI_THICK_CDK_PARSTGRISRH  SMRI_THICK_CDK_PTCATERH  SMRI_THICK_CDK_PCRH  SMRI_THICK_CDK_RRACATERH  SMRI_THICK_CDK_RRMDFRRH  SMRI_THICK_CDK_SUFRRH  SMRI_THICK_CDK_SUPLRH  SMRI_THICK_CDK_SUTMRH  SMRI_THICK_CDK_FRPOLERH  SMRI_THICK_CDK_TMPOLERH  SMRI_THICK_CDK_TRVTMRH  SMRI_THICK_CDK_INSULARH  SMRI_THICK_CDK_MEANLH  SMRI_THICK_CDK_MEANRH  SMRI_THICK_CDK_MEAN | Cortical thickness in mm of APARC ROI lh-Banks of Superior Temporal Sulcus  Cortical thickness in mm of APARC ROI lh-caudalanteriorcingulate  Cortical thickness in mm of APARC ROI lh-caudalmiddlefrontal  Cortical thickness in mm of APARC ROI lh-fusiform  Cortical thickness in mm of APARC ROI lh-inferiorparietal  Cortical thickness in mm of APARC ROI lh-inferiortemporal  Cortical thickness in mm of APARC ROI lh-isthmuscingulate  Cortical thickness in mm of APARC ROI lh-lateralorbitofrontal  Cortical thickness in mm of APARC ROI lh-medialorbitofrontal  Cortical thickness in mm of APARC ROI lh-middletemporal  Cortical thickness in mm of APARC ROI lh-parahippocampal  Cortical thickness in mm of APARC ROI lh-parsopercularis  Cortical thickness in mm of APARC ROI lh-parsorbitalis  Cortical thickness in mm of APARC ROI lh-parstriangularis  Cortical thickness in mm of APARC ROI lh-posteriorcingulate  Cortical thickness in mm of APARC ROI lh-precuneus  Cortical thickness in mm of APARC ROI lh-rostralanteriorcingulate  Cortical thickness in mm of APARC ROI lh-rostralmiddlefrontal  Cortical thickness in mm of APARC ROI lh-superiorfrontal  Cortical thickness in mm of APARC ROI lh-superiorparietal  Cortical thickness in mm of APARC ROI lh-superiortemporal  Cortical thickness in mm of APARC ROI lh-frontalpole  Cortical thickness in mm of APARC ROI lh-temporalpole  Cortical thickness in mm of APARC ROI lh-transversetemporal  Cortical thickness in mm of APARC ROI lh-insula  Cortical thickness in mm of APARC ROI rh-Banks of Superior Temporal Sulcus  Cortical thickness in mm of APARC ROI rh-caudalanteriorcingulate  Cortical thickness in mm of APARC ROI rh-caudalmiddlefrontal  Cortical thickness in mm of APARC ROI rh-fusiform  Cortical thickness in mm of APARC ROI rh-inferiorparietal  Cortical thickness in mm of APARC ROI rh-inferiortemporal  Cortical thickness in mm of APARC ROI rh-isthmuscingulate  Cortical thickness in mm of APARC ROI rh-lateralorbitofrontal  Cortical thickness in mm of APARC ROI rh-medialorbitofrontal  Cortical thickness in mm of APARC ROI rh-middletemporal  Cortical thickness in mm of APARC ROI rh-parahippocampal  Cortical thickness in mm of APARC ROI rh-parsopercularis  Cortical thickness in mm of APARC ROI rh-parsorbitalis  Cortical thickness in mm of APARC ROI rh-parstriangularis  Cortical thickness in mm of APARC ROI rh-posteriorcingulate  Cortical thickness in mm of APARC ROI rh-precuneus  Cortical thickness in mm of APARC ROI rh-rostralanteriorcingulate  Cortical thickness in mm of APARC ROI rh-rostralmiddlefrontal  Cortical thickness in mm of APARC ROI rh-superiorfrontal  Cortical thickness in mm of APARC ROI rh-superiorparietal  Cortical thickness in mm of APARC ROI rh-superiortemporal  Cortical thickness in mm of APARC ROI rh-frontalpole  Cortical thickness in mm of APARC ROI rh-temporalpole  Cortical thickness in mm of APARC ROI rh-transversetemporal  Cortical thickness in mm of APARC ROI rh-insula  Mean cortical thickness in mm for left hemisphere  Mean cortical thickness in mm for right hemisphere  Mean cortical thickness in mm for whole brain |
| **Surface area** | SMRI_AREA_CDK_BANKSSTSLH  SMRI_AREA_CDK_CDACATELH  SMRI_AREA_CDK_CDMDFRLH  SMRI_AREA_CDK_FUSIFORMLH  SMRI_AREA_CDK_IFPLLH  SMRI_AREA_CDK_IFTMLH  SMRI_AREA_CDK_IHCATELH  SMRI_AREA_CDK_LOBFRLH  SMRI_AREA_CDK_MOBFRLH  SMRI_AREA_CDK_MDTMLH  SMRI_AREA_CDK_PARAHPALLH  SMRI_AREA_CDK_PARSOPCLH  SMRI_AREA_CDK_PARSOBISLH  SMRI_AREA_CDK_PARSTGRISLH  SMRI_AREA_CDK_PTCATELH  SMRI_AREA_CDK_PCLH  SMRI_AREA_CDK_RRACATELH  SMRI_AREA_CDK_RRMDFRLH  SMRI_AREA_CDK_SUFRLH  SMRI_AREA_CDK_SUPLLH  SMRI_AREA_CDK_SUTMLH  SMRI_AREA_CDK_FRPOLELH  SMRI_AREA_CDK_TMPOLELH  SMRI_AREA_CDK_TRVTMLH  SMRI_AREA_CDK_INSULALH  SMRI_AREA_CDK_BANKSSTSRH  SMRI_AREA_CDK_CDACATERH  SMRI_AREA_CDK_CDMDFRRH  SMRI_AREA_CDK_FUSIFORMRH  SMRI_AREA_CDK_IFPLRH  SMRI_AREA_CDK_IFTMRH  SMRI_AREA_CDK_IHCATERH  SMRI_AREA_CDK_LOBFRRH  SMRI_AREA_CDK_MOBFRRH  SMRI_AREA_CDK_MDTMRH  SMRI_AREA_CDK_PARAHPALRH  SMRI_AREA_CDK_PARSOPCRH  SMRI_AREA_CDK_PARSOBISRH  SMRI_AREA_CDK_PARSTGRISRH  SMRI_AREA_CDK_PTCATERH  SMRI_AREA_CDK_PCRH  SMRI_AREA_CDK_RRACATERH  SMRI_AREA_CDK_RRMDFRRH  SMRI_AREA_CDK_SUFRRH  SMRI_AREA_CDK_SUPLRH  SMRI_AREA_CDK_SUTMRH  SMRI_AREA_CDK_FRPOLERH  SMRI_AREA_CDK_TMPOLERH  SMRI_AREA_CDK_TRVTMRH  SMRI_AREA_CDK_INSULARH  SMRI_AREA_CDK_MEANLH  SMRI_AREA_CDK_MEANRH  SMRI_AREA_CDK_MEAN | Cortical area in mm of APARC ROI lh-Banks of Superior Temporal Sulcus  Cortical area in mm of APARC ROI lh-caudalanteriorcingulate  Cortical area in mm of APARC ROI lh-caudalmiddlefrontal  Cortical area in mm of APARC ROI lh-fusiform  Cortical area in mm of APARC ROI lh-inferiorparietal  Cortical area in mm of APARC ROI lh-inferiortemporal  Cortical area in mm of APARC ROI lh-isthmuscingulate  Cortical area in mm of APARC ROI lh-lateralorbitofrontal  Cortical area in mm of APARC ROI lh-medialorbitofrontal  Cortical area in mm of APARC ROI lh-middletemporal  Cortical area in mm of APARC ROI lh-parahippocampal  Cortical area in mm of APARC ROI lh-parsopercularis  Cortical area in mm of APARC ROI lh-parsorbitalis  Cortical area in mm of APARC ROI lh-parstriangularis  Cortical area in mm of APARC ROI lh-posteriorcingulate  Cortical area in mm of APARC ROI lh-precuneus  Cortical area in mm of APARC ROI lh-rostralanteriorcingulate  Cortical area in mm of APARC ROI lh-rostralmiddlefrontal  Cortical area in mm of APARC ROI lh-superiorfrontal  Cortical area in mm of APARC ROI lh-superiorparietal  Cortical area in mm of APARC ROI lh-superiortemporal  Cortical area in mm of APARC ROI lh-frontalpole  Cortical area in mm of APARC ROI lh-temporalpole  Cortical area in mm of APARC ROI lh-transversetemporal  Cortical area in mm of APARC ROI lh-insula  Cortical area in mm of APARC ROI rh-Banks of Superior Temporal Sulcus  Cortical area in mm of APARC ROI rh-caudalanteriorcingulate  Cortical area in mm of APARC ROI rh-caudalmiddlefrontal  Cortical area in mm of APARC ROI rh-fusiform  Cortical area in mm of APARC ROI rh-inferiorparietal  Cortical area in mm of APARC ROI rh-inferiortemporal  Cortical area in mm of APARC ROI rh-isthmuscingulate  Cortical area in mm of APARC ROI rh-lateralorbitofrontal  Cortical area in mm of APARC ROI rh-medialorbitofrontal  Cortical area in mm of APARC ROI rh-middletemporal  Cortical area in mm of APARC ROI rh-parahippocampal  Cortical area in mm of APARC ROI rh-parsopercularis  Cortical area in mm of APARC ROI rh-parsorbitalis  Cortical area in mm of APARC ROI rh-parstriangularis  Cortical area in mm of APARC ROI rh-posteriorcingulate  Cortical area in mm of APARC ROI rh-precuneus  Cortical area in mm of APARC ROI rh-rostralanteriorcingulate  Cortical area in mm of APARC ROI rh-rostralmiddlefrontal  Cortical area in mm of APARC ROI rh-superiorfrontal  Cortical area in mm of APARC ROI rh-superiorparietal  Cortical area in mm of APARC ROI rh-superiortemporal  Cortical area in mm of APARC ROI rh-frontalpole  Cortical area in mm of APARC ROI rh-temporalpole  Cortical area in mm of APARC ROI rh-transversetemporal  Cortical area in mm of APARC ROI rh-insula  Mean cortical area in mm for left hemisphere  Mean cortical area in mm for right hemisphere  Mean cortical area in mm for whole brain |
| **Cortical volume** | SMRI_VOL_CDK_BANKSSTSLH  SMRI_VOL_CDK_CDACATELH  SMRI_VOL_CDK_CDMDFRLH  SMRI_VOL_CDK_FUSIFORMLH  SMRI_VOL_CDK_IFPLLH  SMRI_VOL_CDK_IFTMLH  SMRI_VOL_CDK_IHCATELH  SMRI_VOL_CDK_LOBFRLH  SMRI_VOL_CDK_MOBFRLH  SMRI_VOL_CDK_MDTMLH  SMRI_VOL_CDK_PARAHPALLH  SMRI_VOL_CDK_PARSOPCLH  SMRI_VOL_CDK_PARSOBISLH  SMRI_VOL_CDK_PARSTGRISLH  SMRI_VOL_CDK_PTCATELH  SMRI_VOL_CDK_PCLH  SMRI_VOL_CDK_RRACATELH  SMRI_VOL_CDK_RRMDFRLH  SMRI_VOL_CDK_SUFRLH  SMRI_VOL_CDK_SUPLLH  SMRI_VOL_CDK_SUTMLH  SMRI_VOL_CDK_FRPOLELH  SMRI_VOL_CDK_TMPOLELH  SMRI_VOL_CDK_TRVTMLH  SMRI_VOL_CDK_INSULALH  SMRI_VOL_CDK_BANKSSTSRH  SMRI_VOL_CDK_CDACATERH  SMRI_VOL_CDK_CDMDFRRH  SMRI_VOL_CDK_FUSIFORMRH  SMRI_VOL_CDK_IFPLRH  SMRI_VOL_CDK_IFTMRH  SMRI_VOL_CDK_IHCATERH  SMRI_VOL_CDK_LOBFRRH  SMRI_VOL_CDK_MOBFRRH  SMRI_VOL_CDK_MDTMRH  SMRI_VOL_CDK_PARAHPALRH  SMRI_VOL_CDK_PARSOPCRH  SMRI_VOL_CDK_PARSOBISRH  SMRI_VOL_CDK_PARSTGRISRH  SMRI_VOL_CDK_PTCATERH  SMRI_VOL_CDK_PCRH  SMRI_VOL_CDK_RRACATERH  SMRI_VOL_CDK_RRMDFRRH  SMRI_VOL_CDK_SUFRRH  SMRI_VOL_CDK_SUPLRH  SMRI_VOL_CDK_SUTMRH  SMRI_VOL_CDK_FRPOLERH  SMRI_VOL_CDK_TMPOLERH  SMRI_VOL_CDK_TRVTMRH  SMRI_VOL_CDK_INSULARH  SMRI_VOL_CDK_MEANLH  SMRI_VOL_CDK_MEANRH  SMRI_VOL_CDK_MEAN | Cortical volume in mm of APARC ROI lh-Banks of Superior Temporal Sulcus  Cortical volume in mm of APARC ROI lh-caudalanteriorcingulate  Cortical volume in mm of APARC ROI lh-caudalmiddlefrontal  Cortical volume in mm of APARC ROI lh-fusiform  Cortical volume in mm of APARC ROI lh-inferiorparietal  Cortical volume in mm of APARC ROI lh-inferiortemporal  Cortical volume in mm of APARC ROI lh-isthmuscingulate  Cortical volume in mm of APARC ROI lh-lateralorbitofrontal  Cortical volume in mm of APARC ROI lh-medialorbitofrontal  Cortical volume in mm of APARC ROI lh-middletemporal  Cortical volume in mm of APARC ROI lh-parahippocampal  Cortical volume in mm of APARC ROI lh-parsopercularis  Cortical volume in mm of APARC ROI lh-parsorbitalis  Cortical volume in mm of APARC ROI lh-parstriangularis  Cortical volume in mm of APARC ROI lh-posteriorcingulate  Cortical volume in mm of APARC ROI lh-precuneus  Cortical volume in mm of APARC ROI lh-rostralanteriorcingulate  Cortical volume in mm of APARC ROI lh-rostralmiddlefrontal  Cortical volume in mm of APARC ROI lh-superiorfrontal  Cortical volume in mm of APARC ROI lh-superiorparietal  Cortical volume in mm of APARC ROI lh-superiortemporal  Cortical volume in mm of APARC ROI lh-frontalpole  Cortical volume in mm of APARC ROI lh-temporalpole  Cortical volume in mm of APARC ROI lh-transversetemporal  Cortical volume in mm of APARC ROI lh-insula  Cortical volume in mm of APARC ROI rh-Banks of Superior Temporal Sulcus  Cortical volume in mm of APARC ROI rh-caudalanteriorcingulate  Cortical volume in mm of APARC ROI rh-caudalmiddlefrontal  Cortical volume in mm of APARC ROI rh-fusiform  Cortical volume in mm of APARC ROI rh-inferiorparietal  Cortical volume in mm of APARC ROI rh-inferiortemporal  Cortical volume in mm of APARC ROI rh-isthmuscingulate  Cortical volume in mm of APARC ROI rh-lateralorbitofrontal  Cortical volume in mm of APARC ROI rh-medialorbitofrontal  Cortical volume in mm of APARC ROI rh-middletemporal  Cortical volume in mm of APARC ROI rh-parahippocampal  Cortical volume in mm of APARC ROI rh-parsopercularis  Cortical volume in mm of APARC ROI rh-parsorbitalis  Cortical volume in mm of APARC ROI rh-parstriangularis  Cortical volume in mm of APARC ROI rh-posteriorcingulate  Cortical volume in mm of APARC ROI rh-precuneus  Cortical volume in mm of APARC ROI rh-rostralanteriorcingulate  Cortical volume in mm of APARC ROI rh-rostralmiddlefrontal  Cortical volume in mm of APARC ROI rh-superiorfrontal  Cortical volume in mm of APARC ROI rh-superiorparietal  Cortical volume in mm of APARC ROI rh-superiortemporal  Cortical volume in mm of APARC ROI rh-frontalpole  Cortical volume in mm of APARC ROI rh-temporalpole  Cortical volume in mm of APARC ROI rh-transversetemporal  Cortical volume in mm of APARC ROI rh-insula  Mean cortical volume in mm for left hemisphere  Mean cortical volume in mm for right hemisphere  Mean cortical volume in mm for whole brain |
| **Subcortical volume** | SMRI_VOL_SCS_TPLH  SMRI_VOL_SCS_CAUDATELH  SMRI_VOL_SCS_PUTAMENLH  SMRI_VOL_SCS_PALLIDUMLH  SMRI_VOL_SCS_HPUSLH  SMRI_VOL_SCS_AMYGDALALH  SMRI_VOL_SCS_AAL  SMRI_VOL_SCS_TPRH  SMRI_VOL_SCS_CAUDATERH  SMRI_VOL_SCS_PUTAMENRH  SMRI_VOL_SCS_PALLIDUMRH  SMRI_VOL_SCS_HPUSRH  SMRI_VOL_SCS_AMYGDALARH  SMRI_VOL_SCS_AAR  SMRI_VOL_SCS_INTRACRANIALV | Volume in mm³ of ASEG ROI left-thalamus-proper  Volume in mm³ of ASEG ROI left-caudate  Volume in mm³ of ASEG ROI left-putamen  Volume in mm³ of ASEG ROI left-pallidum  Volume in mm³ of ASEG ROI left-hippocampus  Volume in mm³ of ASEG ROI left-amygdala  Volume in mm³ of ASEG ROI left-accumbens-area  Volume in mm³ of ASEG ROI right-thalamus-proper  Volume in mm³ of ASEG ROI right-caudate  Volume in mm³ of ASEG ROI right-putamen  Volume in mm³ of ASEG ROI right -pallidum  Volume in mm³ of ASEG ROI right -hippocampus  Volume in mm³ of ASEG ROI right -amygdala  Volume in mm³ of ASEG ROI right -accumbens-area  Volume in mm³ of ASEG ROI intracranialvolume |
| **fMRI** | | |
| **Resting-state networks** | rsfmri_c_ngd_cgc_ngd_cgc rsfmri_c_ngd_cgc_ngd_ca rsfmri_c_ngd_cgc_ngd_dt rsfmri_c_ngd_cgc_ngd_dla rsfmri_c_ngd_cgc_ngd_fo rsfmri_c_ngd_cgc_ngd_sa rsfmri_c_ngd_cgc_ngd_vta rsfmri_c_ngd_ca_ngd_ca rsfmri_c_ngd_ca_ngd_dt rsfmri_c_ngd_ca_ngd_dla rsfmri_c_ngd_ca_ngd_fo rsfmri_c_ngd_ca_ngd_sa rsfmri_c_ngd_ca_ngd_vta rsfmri_c_ngd_dt_ngd_dt rsfmri_c_ngd_dt_ngd_dla rsfmri_c_ngd_dt_ngd_fo rsfmri_c_ngd_dt_ngd_sa rsfmri_c_ngd_dt_ngd_vta rsfmri_c_ngd_dla_ngd_dla rsfmri_c_ngd_dla_ngd_fo rsfmri_c_ngd_dla_ngd_sa rsfmri_c_ngd_dla_ngd_vta rsfmri_c_ngd_fo_ngd_fo rsfmri_c_ngd_fo_ngd_sa rsfmri_c_ngd_fo_ngd_vta rsfmri_c_ngd_sa_ngd_sa rsfmri_c_ngd_sa_ngd_vta rsfmri_c_ngd_vta_ngd_vta | Resting-state fMRI connectivity between Cingulate Gyrus and Cingulate Gyrus  Resting-state fMRI connectivity between Cingulate Gyrus and CinguloParietal  Resting-state fMRI connectivity between Cingulate Gyrus and Default Mode Network  Resting-state fMRI connectivity between Cingulate Gyrus and Dorsal Attention Network  Resting-state fMRI connectivity between Cingulate Gyrus and Frontoparietal Network  Resting-state fMRI connectivity between Cingulate Gyrus and Salience Network  Resting-state fMRI connectivity between Cingulate Gyrus and Ventral Attention Network  Resting-state fMRI connectivity between CinguloParietal and CinguloParietal  Resting-state fMRI connectivity between CinguloParietal and Default Mode Network  Resting-state fMRI connectivity between CinguloParietal and Dorsal Attention Network  Resting-state fMRI connectivity between CinguloParietal and Frontoparietal Network  Resting-state fMRI connectivity between CinguloParietal and Salience Network  Resting-state fMRI connectivity between CinguloParietal and Ventral Attention Network  Resting-state fMRI connectivity between Default Mode Network and Default Mode Network  Resting-state fMRI connectivity between Default Mode Network and Dorsal Attention Network  Resting-state fMRI connectivity between Default Mode Network and Frontoparietal Network  Resting-state fMRI connectivity between Default Mode Network and Salience Network  Resting-state fMRI connectivity between Default Mode Network and Ventral Attention Network  Resting-state fMRI connectivity between Dorsal Attention Network and Dorsal Attention Network  Resting-state fMRI connectivity between Dorsal Attention Network and Frontoparietal Network  Resting-state fMRI connectivity between Dorsal Attention Network and Salience Network  Resting-state fMRI connectivity between Dorsal Attention Network and Ventral Attention Network  Resting-state fMRI connectivity between Frontoparietal Network and Frontoparietal Network  Resting-state fMRI connectivity between Frontoparietal Network and Salience Network  Resting-state fMRI connectivity between Frontoparietal Network and Ventral Attention Network  Resting-state fMRI connectivity between Salience Network and Salience Network  Resting-state fMRI connectivity between Salience Network and Visual Network  Resting-state fMRI connectivity between Ventral Attention Network and Ventral Attention Network |

**Table S.2. Distribution of participants across PRS-SCZ and air pollutant tertiles.**

|  | **Low PRS-SCZ** | **Medium PRS-SCZ** | **High PRS-SCZ** |
| --- | --- | --- | --- |
| **PM₂.₅** | | | |
| **Low PM₂.₅** | 205 | 185 | 135 |
| **Medium PM₂.₅** | 193 | 198 | 152 |
| **High PM₂.₅** | 195 | 174 | 143 |
| **NO₂** | | | |
| **Low PM₂.₅** | 165 | 144 | 216 |
| **Medium PM₂.₅** | 180 | 146 | 217 |
| **High PM₂.₅** | 181 | 131 | 200 |
| **O₃** | | | |
| **Low O₃** | 211 | 182 | 132 |
| **Medium O₃** | 213 | 167 | 163 |
| **High O₃** | 204 | 172 | 136 |

**Table S.3. Distribution of air pollutants levels across sites.**

| **Site** | **n of participants** | **PM₂.₅ mean (SD)** | **NO₂ mean (SD)** | **O₃ mean (SD)** |
| --- | --- | --- | --- | --- |
| **site01** | 13 | 9.164 (1.321) | 19.589 (2.779) | 42.534 (5.758) |
| **site02** | 148 | 6.021 (1.329) | 26.11 (6.305) | 38.685 (2.527) |
| **site03** | 14 | 7.759 (0.554) | 16.381 (1.577) | 47.17 (2.037) |
| **site04** | 141 | 8.869 (0.321) | 15.245 (2.406) | 36.011 (1.16) |
| **site05** | 80 | 7.35 (0.699) | 11.231 (3.003) | 39.433 (1.28) |
| **site06** | 115 | 6.175 (1.158) | 19.36 (2.423) | 43.116 (3.995) |
| **site07** | 6 | 5.949 (0.351) | 14.514 (4.167) | 47.329 (1.084) |
| **site08** | 36 | 7.718 (1.064) | 15.565 (3.482) | 42.467 (4.314) |
| **site09** | 28 | 9.231 (0.909) | 18.818 (1.839) | 41.262 (5.223) |
| **site10** | 37 | 8.005 (0.704) | 16.916 (2.624) | 40.503 (4.414) |
| **site11** | 35 | 7.156 (0.864) | 11.6 (5.419) | 47.279 (2.601) |
| **site12** | 65 | 7.722 (1.138) | 24.218 (4.748) | 41.085 (4.936) |
| **site13** | 108 | 8.32 (0.627) | 21.398 (3.604) | 40.086 (1.026) |
| **site14** | 80 | 6.323 (0.523) | 13.496 (3.024) | 39.837 (2.734) |
| **site15** | 33 | 10.048 (1.843) | 17.161 (5.044) | 40.337 (2.537) |
| **site16** | 212 | 7.966 (1.014) | 24.433 (4.154) | 41.802 (5.015) |
| **site17** | 59 | 5.008 (0.57) | 11.725 (2.701) | 42.863 (2.877) |
| **site18** | 86 | 7.295 (0.564) | 21.85 (1.994) | 39.356 (2.009) |
| **site19** | 76 | 7.337 (0.685) | 14.169 (4.508) | 45.925 (4.024) |
| **site20** | 99 | 7.93 (0.503) | 17.784 (4.633) | 37.557 (2.021) |
| **site21** | 109 | 6.823 (0.97) | 21.058 (4.909) | 45.392 (2.53) |

**Table S.4. Distribution of participants stratified according to income across sites.**

| **Site** | **Low income**  **n (%)** | **Middle income n (%)** | **High income**  **n (%)** | **NA**  **n (%)** |
| --- | --- | --- | --- | --- |
| **site01** | 1 (7.69) | 1 (7.69) | 9 (69.23) | 2 (15.38) |
| **site02** | 11 (7.43) | 39 (26.35) | 93 (62.84) | 5 (3.38) |
| **site03** | 6 (42.86) | 3 (21.43) | 3 (21.43) | 2 (14.29) |
| **site04** | 32 (22.7) | 52 (36.88) | 50 (35.46) | 7 (4.96) |
| **site05** | 10 (12.5) | 16 (20) | 47 (58.75) | 7 (8.75) |
| **site06** | 13 (11.3) | 43 (37.39) | 58 (50.43) | 1 (0.87) |
| **site07** | 1 (16.67) | 1 (16.67) | 4 (66.67) | 0 (0) |
| **site08** | 2 (5.56) | 6 (16.67) | 26 (72.22) | 2 (5.56) |
| **site09** | 3 (10.71) | 4 (14.29) | 19 (67.86) | 2 (7.14) |
| **site10** | 1 (2.7) | 9 (24.32) | 23 (62.16) | 4 (10.81) |
| **site11** | 8 (22.86) | 15 (42.86) | 10 (28.57) | 2 (5.71) |
| **site12** | 1 (1.54) | 19 (29.23) | 42 (64.62) | 3 (4.62) |
| **site13** | 18 (16.67) | 33 (30.56) | 53 (49.07) | 4 (3.7) |
| **site14** | 3 (3.75) | 22 (27.5) | 51 (63.75) | 4 (5) |
| **site15** | 12 (36.36) | 10 (30.3) | 8 (24.24) | 3 (9.09) |
| **site16** | 36 (16.98) | 93 (43.87) | 81 (38.21) | 2 (0.94) |
| **site17** | 5 (8.47) | 15 (25.42) | 38 (64.41) | 1 (1.69) |
| **site18** | 5 (5.81) | 26 (30.23) | 53 (61.63) | 2 (2.33) |
| **site19** | 0 (0) | 27 (35.53) | 47 (61.84) | 2 (2.63) |
| **site20** | 17 (17.17) | 37 (37.37) | 43 (43.43) | 2 (2.02) |
| **site21** | 8 (7.34) | 23 (21.1) | 72 (66.06) | 6 (5.5) |

**Table S.5. Distribution of participants stratified according to education level.**

| **Site** | **Postgraduate degree**  **n (%)** | **Bachelor's degree**  **n (%)** | **Some college**  **n (%)** | **High school/GED**  **n (%)** | **Less than high school**  **n (%)** |
| --- | --- | --- | --- | --- | --- |
| **site01** | 7 (53.85) | 5 (38.46) | 0 (0) | 1 (7.69) | 0 (0) |
| **site02** | 57 (38.51) | 67 (45.27) | 23 (15.54) | 1 (0.68) | 0 (0) |
| **site03** | 1 (7.14) | 8 (57.14) | 3 (21.43) | 2 (14.29) | 0 (0) |
| **site04** | 57 (40.43) | 32 (22.7) | 39 (27.66) | 12 (8.51) | 1 (0.71) |
| **site05** | 45 (56.25) | 24 (30) | 9 (11.25) | 2 (2.5) | 0 (0) |
| **site06** | 49 (42.61) | 39 (33.91) | 25 (21.74) | 2 (1.74) | 0 (0) |
| **site07** | 3 (50) | 2 (33.33) | 1 (16.67) | 0 (0) | 0 (0) |
| **site08** | 24 (66.67) | 10 (27.78) | 1 (2.78) | 1 (2.78) | 0 (0) |
| **site09** | 9 (32.14) | 15 (53.57) | 4 (14.29) | 0 (0) | 0 (0) |
| **site10** | 17 (45.95) | 14 (37.84) | 6 (16.22) | 0 (0) | 0 (0) |
| **site11** | 16 (45.71) | 8 (22.86) | 8 (22.86) | 2 (5.71) | 1 (2.86) |
| **site12** | 44 (67.69) | 14 (21.54) | 4 (6.15) | 3 (4.62) | 0 (0) |
| **site13** | 52 (48.15) | 27 (25) | 25 (23.15) | 3 (2.78) | 1 (0.93) |
| **site14** | 31 (38.75) | 36 (45) | 13 (16.25) | 0 (0) | 0 (0) |
| **site15** | 7 (21.21) | 13 (39.39) | 11 (33.33) | 2 (6.06) | 0 (0) |
| **site16** | 75 (35.38) | 84 (39.62) | 51 (24.06) | 2 (0.94) | 0 (0) |
| **site17** | 38 (64.41) | 15 (25.42) | 5 (8.47) | 1 (1.69) | 0 (0) |
| **site18** | 52 (60.47) | 27 (31.4) | 6 (6.98) | 1 (1.16) | 0 (0) |
| **site19** | 33 (43.42) | 28 (36.84) | 15 (19.74) | 0 (0) | 0 (0) |
| **site20** | 32 (32.32) | 26 (26.26) | 34 (34.34) | 3 (3.03) | 4 (4.04) |
| **site21** | 63 (57.8) | 26 (23.85) | 14 (12.84) | 6 (5.5) | 0 (0) |

**Table S.6. Results from linear mixed-effects models testing the effects of PM₂.₅ tertile on surface area (fully adjusted model).**

| **Surface area** | **term** | **F** | **p-FDR** |
| --- | --- | --- | --- |
| Cortical area in mm of APARC ROI lh-Banks of Superior Temporal Sulcus | PM₂.₅tert | 3.335 | 0.958 |
| Cortical area in mm of APARC ROI lh-caudalanteriorcingulate | PM₂.₅tert | 0.508 | 0.958 |
| Cortical area in mm of APARC ROI lh-caudalmiddlefrontal | PM₂.₅tert | 1.152 | 0.958 |
| Cortical area in mm of APARC ROI lh-fusiform | PM₂.₅tert | 0.854 | 0.958 |
| Cortical area in mm of APARC ROI lh-inferiorparietal | PM₂.₅tert | 1.776 | 0.958 |
| Cortical area in mm of APARC ROI lh-inferiortemporal | PM₂.₅tert | 0.472 | 0.958 |
| Cortical area in mm of APARC ROI lh-isthmuscingulate | PM₂.₅tert | 0.667 | 0.958 |
| Cortical area in mm of APARC ROI lh-lateralorbitofrontal | PM₂.₅tert | 0.633 | 0.958 |
| Cortical area in mm of APARC ROI lh-medialorbitofrontal | PM₂.₅tert | 1.229 | 0.958 |
| Cortical area in mm of APARC ROI lh-middletemporal | PM₂.₅tert | 1.003 | 0.958 |
| Cortical area in mm of APARC ROI lh-parahippocampal | PM₂.₅tert | 0.613 | 0.958 |
| Cortical area in mm of APARC ROI lh-parsopercularis | PM₂.₅tert | 1.143 | 0.958 |
| Cortical area in mm of APARC ROI lh-parsorbitalis | PM₂.₅tert | 0.158 | 0.958 |
| Cortical area in mm of APARC ROI lh-parstriangularis | PM₂.₅tert | 0.152 | 0.958 |
| Cortical area in mm of APARC ROI lh-posteriorcingulate | PM₂.₅tert | 0.159 | 0.958 |
| Cortical area in mm of APARC ROI lh-precuneus | PM₂.₅tert | 0.190 | 0.958 |
| Cortical area in mm of APARC ROI lh-rostralanteriorcingulate | PM₂.₅tert | 0.315 | 0.958 |
| Cortical area in mm of APARC ROI lh-rostralmiddlefrontal | PM₂.₅tert | 0.946 | 0.958 |
| Cortical area in mm of APARC ROI lh-superiorfrontal | PM₂.₅tert | 0.417 | 0.958 |
| Cortical area in mm of APARC ROI lh-superiorparietal | PM₂.₅tert | 1.742 | 0.958 |
| Cortical area in mm of APARC ROI lh-superiortemporal | PM₂.₅tert | 0.417 | 0.958 |
| Cortical area in mm of APARC ROI lh-frontalpole | PM₂.₅tert | 1.846 | 0.958 |
| Cortical area in mm of APARC ROI lh-temporalpole | PM₂.₅tert | 0.992 | 0.958 |
| Cortical area in mm of APARC ROI lh-transversetemporal | PM₂.₅tert | 0.746 | 0.958 |
| Cortical area in mm of APARC ROI lh-insula | PM₂.₅tert | 0.035 | 0.966 |
| Cortical area in mm of APARC ROI rh-Banks of Superior Temporal Sulcus | PM₂.₅tert | 1.172 | 0.958 |
| Cortical area in mm of APARC ROI rh-caudalanteriorcingulate | PM₂.₅tert | 0.257 | 0.958 |
| Cortical area in mm of APARC ROI rh-caudalmiddlefrontal | PM₂.₅tert | 0.202 | 0.958 |
| Cortical area in mm of APARC ROI rh-fusiform | PM₂.₅tert | 0.350 | 0.958 |
| Cortical area in mm of APARC ROI rh-inferiorparietal | PM₂.₅tert | 1.749 | 0.958 |
| Cortical area in mm of APARC ROI rh-inferiortemporal | PM₂.₅tert | 0.994 | 0.958 |
| Cortical area in mm of APARC ROI rh-isthmuscingulate | PM₂.₅tert | 0.506 | 0.958 |
| Cortical area in mm of APARC ROI rh-lateralorbitofrontal | PM₂.₅tert | 0.532 | 0.958 |
| Cortical area in mm of APARC ROI rh-medialorbitofrontal | PM₂.₅tert | 0.142 | 0.958 |
| Cortical area in mm of APARC ROI rh-middletemporal | PM₂.₅tert | 2.004 | 0.958 |
| Cortical area in mm of APARC ROI rh-parahippocampal | PM₂.₅tert | 0.119 | 0.960 |
| Cortical area in mm of APARC ROI rh-parsopercularis | PM₂.₅tert | 0.053 | 0.966 |
| Cortical area in mm of APARC ROI rh-parsorbitalis | PM₂.₅tert | 1.206 | 0.958 |
| Cortical area in mm of APARC ROI rh-parstriangularis | PM₂.₅tert | 0.357 | 0.958 |
| Cortical area in mm of APARC ROI rh-posteriorcingulate | PM₂.₅tert | 0.505 | 0.958 |
| Cortical area in mm of APARC ROI rh-precuneus | PM₂.₅tert | 0.051 | 0.966 |
| Cortical area in mm of APARC ROI rh-rostralanteriorcingulate | PM₂.₅tert | 0.283 | 0.958 |
| Cortical area in mm of APARC ROI rh-rostralmiddlefrontal | PM₂.₅tert | 2.097 | 0.958 |
| Cortical area in mm of APARC ROI rh-superiorfrontal | PM₂.₅tert | 1.024 | 0.958 |
| Cortical area in mm of APARC ROI rh-superiorparietal | PM₂.₅tert | 0.362 | 0.958 |
| Cortical area in mm of APARC ROI rh-superiortemporal | PM₂.₅tert | 0.566 | 0.958 |
| Cortical area in mm of APARC ROI rh-frontalpole | PM₂.₅tert | 7.467 | **0.040** |
| Cortical area in mm of APARC ROI rh-temporalpole | PM₂.₅tert | 0.359 | 0.958 |
| Cortical area in mm of APARC ROI rh-transversetemporal | PM₂.₅tert | 0.795 | 0.958 |
| Cortical area in mm of APARC ROI rh-insula | PM₂.₅tert | 0.064 | 0.966 |
| Mean cortical area in mm for left hemisphere | PM₂.₅tert | 0.402 | 0.958 |
| Mean cortical area in mm for right hemisphere | PM₂.₅tert | 0.354 | 0.958 |
| Mean cortical area in mm for whole brain | PM₂.₅tert | 0.376 | 0.958 |

**Table S.7. Pairwise contrasts for the effects of PM₂.₅ tertile on surface area (fully adjusted model).**

| **Surface area** | **Contrast** | **Estimate** | **SE** | **p-FDR** |
| --- | --- | --- | --- | --- |
| Cortical area in mm of rh-frontal pole | PM₂.₅ Low - PM₂.₅ Medium | -1.153 | 3.348 | 0.937 |
|  | PM₂.₅ Low - PM₂.₅ High | 11.324 | 3.855 | **0.011** |
|  | PM₂.₅ Medium - PM₂.₅ High | 12.476 | 3.521 | **0.004** |

**Table S.8. Pairwise contrasts for the effects of PM₂.₅ tertile and polygenic risk score of schizophrenia tertile on left caudate volume (fully adjusted model).**

| **Contrast** | **Estimate** | **SE** | **p-FDR** |
| --- | --- | --- | --- |
| LowPRS LowPM₂.₅ - MediumPRS LowPM₂.₅ | 14.238 | 39.461 | 0.832 |
| LowPRS LowPM₂.₅ - HighPRS LowPM₂.₅ | 38.799 | 40.010 | 0.522 |
| LowPRS LowPM₂.₅ - LowPRS MediumPM₂.₅ | 92.589 | 43.181 | 0.197 |
| LowPRS LowPM₂.₅ - MediumPRS MediumPM₂.₅ | -41.459 | 42.267 | 0.522 |
| LowPRS LowPM₂.₅ - HighPRS MediumPM₂.₅ | -12.552 | 43.834 | 0.840 |
| LowPRS LowPM₂.₅ - LowPRS High PM₂.₅PM₂.₅ | -62.032 | 48.517 | 0.477 |
| LowPRS LowPM₂.₅ - MediumPRS HighPM₂.₅ | 49.522 | 46.143 | 0.522 |
| LowPRS LowPM₂.₅ - HighPRS HighPM₂.₅ | -1.166 | 47.960 | 0.981 |
| MediumPRS LowPM₂.₅ - HighPRS LowPM₂.₅ | 24.561 | 40.047 | 0.666 |
| MediumPRS LowPM₂.₅ - LowPRS MediumPM₂.₅ | 78.352 | 42.821 | 0.236 |
| MediumPRS LowPM₂.₅ - MediumPRS MediumPM₂.₅ | -55.696 | 41.945 | 0.477 |
| MediumPRS LowPM₂.₅ - HighPRS MediumPM₂.₅ | -26.789 | 43.269 | 0.666 |
| MediumPRS LowPM₂.₅ - LowPRS HighPM₂.₅ | -76.269 | 48.342 | 0.375 |
| MediumPRS LowPM₂.₅ - MediumPRS HighPM₂.₅ | 35.285 | 46.040 | 0.610 |
| MediumPRS LowPM₂.₅ - HighPRS HighPM₂.₅ | -15.403 | 47.779 | 0.840 |
| HighPRS LowPM₂.₅ - LowPRS MediumPM₂.₅ | 53.790 | 43.357 | 0.477 |
| HighPRS LowPM₂.₅ - MediumPRS MediumPM₂.₅ | -80.258 | 42.323 | 0.229 |
| HighPRS LowPM₂.₅ - HighPRS MediumPM₂.₅ | -51.351 | 43.756 | 0.512 |
| HighPRS LowPM₂.₅ - LowPRS HighPM₂.₅ | -100.830 | 49.021 | 0.197 |
| HighPRS LowPM₂.₅ - MediumPRS HighPM₂.₅ | 10.723 | 46.612 | 0.840 |
| HighPRS LowPM₂.₅ - HighPRS HighPM₂.₅ | -39.964 | 48.452 | 0.589 |
| LowPRS MediumPM₂.₅- MediumPRS MediumPM₂.₅ | -134.048 | 40.144 | **0.019** |
| LowPRS MediumPM₂.₅- HighPRS MediumPM₂.₅ | -105.141 | 42.007 | 0.151 |
| LowPRS MediumPM₂.₅- LowPRS HighPM₂.₅ | -154.621 | 47.005 | **0.019** |
| LowPRS MediumPM₂.₅- MediumPRS HighPM₂.₅ | -43.067 | 44.804 | 0.522 |
| LowPRS MediumPM₂.₅- HighPRS HighPM₂.₅ | -93.755 | 46.432 | 0.197 |
| MediumPRS MediumPM₂.₅- HighPRS MediumPM₂.₅ | 28.907 | 40.163 | 0.629 |
| MediumPRS MediumPM₂.₅- LowPRS HighPM₂.₅ | -20.573 | 46.320 | 0.789 |
| MediumPRS MediumPM₂.₅- MediumPRS HighPM₂.₅ | 90.981 | 44.122 | 0.197 |
| MediumPRS MediumPM₂.₅- HighPRS HighPM₂.₅ | 40.293 | 45.816 | 0.570 |
| HighPRS MediumPM₂.₅- LowPRS HighPM₂.₅ | -49.480 | 47.585 | 0.522 |
| HighPRS MediumPM₂.₅- MediumPRS HighPM₂.₅ | 62.074 | 45.646 | 0.477 |
| HighPRS MediumPM₂.₅- HighPRS HighPM₂.₅ | 11.386 | 47.250 | 0.840 |
| LowPRS HighPM₂.₅- MediumPRS HighPM₂.₅ | 111.554 | 47.061 | 0.154 |
| LowPRS HighPM₂.₅- HighPRS HighPM₂.₅ | 60.866 | 48.385 | 0.477 |
| MediumPRS HighPM₂.₅- HighPRS HighPM₂.₅ | -50.688 | 45.787 | 0.522 |

**Table S.9. Pairwise contrasts for the effects of PM₂.₅ tertile and polygenic risk score of schizophrenia tertile on right caudate volume (fully adjusted model).**

| **Contrast** | **Estimate** | **SE** | **p-FDR** |
| --- | --- | --- | --- |
| LowPRS LowPM₂.₅ - MediumPRS LowPM₂.₅ | 10.920 | 38.703 | 0.849 |
| LowPRS LowPM₂.₅ - HighPRS LowPM₂.₅ | 22.148 | 39.307 | 0.764 |
| LowPRS LowPM₂.₅ - LowPRS MediumPM₂.₅ | 73.824 | 42.664 | 0.336 |
| LowPRS LowPM₂.₅ - MediumPRS MediumPM₂.₅ | -43.888 | 41.896 | 0.590 |
| LowPRS LowPM₂.₅ - HighPRS MediumPM₂.₅ | -20.211 | 43.677 | 0.773 |
| LowPRS LowPM₂.₅ - LowPRS High PM₂.₅PM₂.₅ | -88.388 | 48.094 | 0.301 |
| LowPRS LowPM₂.₅ - MediumPRS HighPM₂.₅ | 21.179 | 45.586 | 0.773 |
| LowPRS LowPM₂.₅ - HighPRS HighPM₂.₅ | -20.165 | 47.547 | 0.780 |
| MediumPRS LowPM₂.₅ - HighPRS LowPM₂.₅ | 11.228 | 38.998 | 0.849 |
| MediumPRS LowPM₂.₅ - LowPRS MediumPM₂.₅ | 62.904 | 42.032 | 0.396 |
| MediumPRS LowPM₂.₅ - MediumPRS MediumPM₂.₅ | -54.808 | 41.285 | 0.443 |
| MediumPRS LowPM₂.₅ - HighPRS MediumPM₂.₅ | -31.131 | 42.841 | 0.702 |
| MediumPRS LowPM₂.₅ - LowPRS HighPM₂.₅ | -99.308 | 47.736 | 0.212 |
| MediumPRS LowPM₂.₅ - MediumPRS HighPM₂.₅ | 10.259 | 45.215 | 0.869 |
| MediumPRS LowPM₂.₅ - HighPRS HighPM₂.₅ | -31.084 | 47.166 | 0.735 |
| HighPRS LowPM₂.₅ - LowPRS MediumPM₂.₅ | 51.676 | 42.591 | 0.492 |
| HighPRS LowPM₂.₅ - MediumPRS MediumPM₂.₅ | -66.036 | 41.686 | 0.396 |
| HighPRS LowPM₂.₅ - HighPRS MediumPM₂.₅ | -42.359 | 43.341 | 0.590 |
| HighPRS LowPM₂.₅ - LowPRS HighPM₂.₅ | -110.536 | 48.411 | 0.173 |
| HighPRS LowPM₂.₅ - MediumPRS HighPM₂.₅ | -0.969 | 45.819 | 0.999 |
| HighPRS LowPM₂.₅ - HighPRS HighPM₂.₅ | -42.312 | 47.846 | 0.590 |
| LowPRS MediumPM₂.₅- MediumPRS MediumPM₂.₅ | -117.712 | 39.409 | 0.052 |
| LowPRS MediumPM₂.₅- HighPRS MediumPM₂.₅ | -94.035 | 41.638 | 0.173 |
| LowPRS MediumPM₂.₅- LowPRS HighPM₂.₅ | -162.212 | 46.466 | **0.018** |
| LowPRS MediumPM₂.₅- MediumPRS HighPM₂.₅ | -52.645 | 44.031 | 0.492 |
| LowPRS MediumPM₂.₅- HighPRS HighPM₂.₅ | -93.988 | 45.937 | 0.212 |
| MediumPRS MediumPM₂.₅- HighPRS MediumPM₂.₅ | 23.677 | 39.754 | 0.764 |
| MediumPRS MediumPM₂.₅- LowPRS HighPM₂.₅ | -44.500 | 45.830 | 0.590 |
| MediumPRS MediumPM₂.₅- MediumPRS HighPM₂.₅ | 65.067 | 43.395 | 0.396 |
| MediumPRS MediumPM₂.₅- HighPRS HighPM₂.₅ | 23.724 | 45.350 | 0.773 |
| HighPRS MediumPM₂.₅- LowPRS HighPM₂.₅ | -68.177 | 47.391 | 0.396 |
| HighPRS MediumPM₂.₅- MediumPRS HighPM₂.₅ | 41.390 | 45.250 | 0.590 |
| HighPRS MediumPM₂.₅- HighPRS HighPM₂.₅ | 0.046 | 47.101 | 0.999 |
| LowPRS HighPM₂.₅- MediumPRS HighPM₂.₅ | 109.567 | 46.236 | 0.173 |
| LowPRS HighPM₂.₅- HighPRS HighPM₂.₅ | 68.223 | 47.827 | 0.396 |
| MediumPRS HighPM₂.₅- HighPRS HighPM₂.₅ | -41.343 | 44.843 | 0.590 |

**Table S.10. Results from linear mixed-effects models testing the effects of polygenic risk score of schizophrenia tertile on subcortical volumes (fully adjusted model).**

| **Subcortical volume** | **term** | **F** | **p-FDR** |
| --- | --- | --- | --- |
| Volume in mm³ of left-thalamus-proper | PRS-SCZtert | 0.771 | 0.751 |
| Volume in mm³ of left-caudate | PRS-SCZtert | 0.007 | 0.996 |
| Volume in mm³ of left-putamen | PRS-SCZtert | 2.052 | 0.384 |
| Volume in mm³ of left-pallidum | PRS-SCZtert | 4.213 | 0.072 |
| Volume in mm³ of left-hippocampus | PRS-SCZtert | 0.431 | 0.851 |
| Volume in mm³ of left-amygdala | PRS-SCZtert | 1.086 | 0.668 |
| Volume in mm³ of left-accumbens-area | PRS-SCZtert | 0.459 | 0.847 |
| Volume in mm³ of right-thalamus-proper | PRS-SCZtert | 0.849 | 0.728 |
| Volume in mm³ of right-caudate | PRS-SCZtert | 0.004 | 0.996 |
| Volume in mm³ of right-putamen | PRS-SCZtert | 0.162 | 0.938 |
| Volume in mm³ of right -pallidum | PRS-SCZtert | 0.689 | 0.795 |
| Volume in mm³ of right -hippocampus | PRS-SCZtert | 0.304 | 0.887 |
| Volume in mm³ of right -amygdala | PRS-SCZtert | 1.335 | 0.628 |
| Volume in mm³ of right -accumbens-area | PRS-SCZtert | 4.605 | 0.053 |

**Table S.11. Results from linear mixed-effects models testing the effects of PM₂.₅ tertile on subcortical volumes (fully adjusted model).**

| **Subcortical volume** | **term** | **F** | **p-FDR** |
| --- | --- | --- | --- |
| Volume in mm³ of left-thalamus-proper | PM₂.₅tert | 0.125 | 0.945 |
| Volume in mm³ of left-caudate | PM₂.₅tert | 0.299 | 0.887 |
| Volume in mm³ of left-putamen | PM₂.₅tert | 0.454 | 0.847 |
| Volume in mm³ of left-pallidum | PM₂.₅tert | 0.531 | 0.839 |
| Volume in mm³ of left-hippocampus | PM₂.₅tert | 1.188 | 0.648 |
| Volume in mm³ of left-amygdala | PM₂.₅tert | 1.194 | 0.648 |
| Volume in mm³ of left-accumbens-area | PM₂.₅tert | 0.013 | 0.996 |
| Volume in mm³ of right-thalamus-proper | PM₂.₅tert | 0.507 | 0.839 |
| Volume in mm³ of right-caudate | PM₂.₅tert | 1.015 | 0.674 |
| Volume in mm³ of right-putamen | PM₂.₅tert | 1.313 | 0.628 |
| Volume in mm³ of right -pallidum | PM₂.₅tert | 0.022 | 0.996 |
| Volume in mm³ of right -hippocampus | PM₂.₅tert | 0.877 | 0.720 |
| Volume in mm³ of right -amygdala | PM₂.₅tert | 0.134 | 0.942 |
| Volume in mm³ of right -accumbens-area | PM₂.₅tert | 0.072 | 0.979 |

**Table S.12. Results from linear mixed-effects models testing the interaction effects of PM₂.₅** **tertile and polygenic risk score of schizophrenia tertile on subcortical volumes (fully adjusted model, European ancestry group only).**

| **Subcortical volume** | **term** | **F** | **p-FDR** |
| --- | --- | --- | --- |
| Volume in mm³ of left-thalamus-proper | PRS-SCZtert:PM₂.₅tert | 1.384 | 0.474 |
| Volume in mm³ of left-caudate | PRS-SCZtert:PM₂.₅tert | 4.324 | **0.025** |
| Volume in mm³ of left-putamen | PRS-SCZtert:PM₂.₅tert | 1.877 | 0.306 |
| Volume in mm³ of left-pallidum | PRS-SCZtert:PM₂.₅tert | 1.000 | 0.685 |
| Volume in mm³ of left-hippocampus | PRS-SCZtert:PM₂.₅tert | 0.570 | 0.866 |
| Volume in mm³ of left-amygdala | PRS-SCZtert:PM₂.₅tert | 0.306 | 0.874 |
| Volume in mm³ of left-accumbens-area | PRS-SCZtert:PM₂.₅tert | 0.939 | 0.685 |
| Volume in mm³ of right-thalamus-proper | PRS-SCZtert:PM₂.₅tert | 2.026 | 0.306 |
| Volume in mm³ of right-caudate | PRS-SCZtert:PM₂.₅tert | 3.859 | **0.028** |
| Volume in mm³ of right-putamen | PRS-SCZtert:PM₂.₅tert | 2.769 | 0.122 |
| Volume in mm³ of right -pallidum | PRS-SCZtert:PM₂.₅tert | 0.481 | 0.866 |
| Volume in mm³ of right -hippocampus | PRS-SCZtert:PM₂.₅tert | 0.406 | 0.866 |
| Volume in mm³ of right -amygdala | PRS-SCZtert:PM₂.₅tert | 1.776 | 0.306 |
| Volume in mm³ of right -accumbens-area | PRS-SCZtert:PM₂.₅tert | 0.577 | 0.866 |

**Table S.13. Results from linear mixed-effects models testing the effects of PM₂.₅ tertile on surface area including effects of population density and distance to road (fully adjusted model, sensitivity analysis).**

| **Surface area** | **term** | **F** | **p-FDR** |
| --- | --- | --- | --- |
| Cortical area in mm of APARC ROI lh-Banks of Superior Temporal Sulcus | PM₂.₅tert | 3.188 | 0.933 |
| Cortical area in mm of APARC ROI lh-caudalanteriorcingulate | PM₂.₅tert | 0.428 | 0.933 |
| Cortical area in mm of APARC ROI lh-caudalmiddlefrontal | PM₂.₅tert | 1.041 | 0.933 |
| Cortical area in mm of APARC ROI lh-fusiform | PM₂.₅tert | 0.860 | 0.933 |
| Cortical area in mm of APARC ROI lh-inferiorparietal | PM₂.₅tert | 1.695 | 0.933 |
| Cortical area in mm of APARC ROI lh-inferiortemporal | PM₂.₅tert | 0.438 | 0.933 |
| Cortical area in mm of APARC ROI lh-isthmuscingulate | PM₂.₅tert | 0.680 | 0.933 |
| Cortical area in mm of APARC ROI lh-lateralorbitofrontal | PM₂.₅tert | 0.671 | 0.933 |
| Cortical area in mm of APARC ROI lh-medialorbitofrontal | PM₂.₅tert | 1.150 | 0.933 |
| Cortical area in mm of APARC ROI lh-middletemporal | PM₂.₅tert | 1.095 | 0.933 |
| Cortical area in mm of APARC ROI lh-parahippocampal | PM₂.₅tert | 0.624 | 0.933 |
| Cortical area in mm of APARC ROI lh-parsopercularis | PM₂.₅tert | 1.281 | 0.933 |
| Cortical area in mm of APARC ROI lh-parsorbitalis | PM₂.₅tert | 0.110 | 0.933 |
| Cortical area in mm of APARC ROI lh-parstriangularis | PM₂.₅tert | 0.123 | 0.933 |
| Cortical area in mm of APARC ROI lh-posteriorcingulate | PM₂.₅tert | 0.105 | 0.933 |
| Cortical area in mm of APARC ROI lh-precuneus | PM₂.₅tert | 0.270 | 0.933 |
| Cortical area in mm of APARC ROI lh-rostralanteriorcingulate | PM₂.₅tert | 0.318 | 0.933 |
| Cortical area in mm of APARC ROI lh-rostralmiddlefrontal | PM₂.₅tert | 0.659 | 0.933 |
| Cortical area in mm of APARC ROI lh-superiorfrontal | PM₂.₅tert | 0.315 | 0.933 |
| Cortical area in mm of APARC ROI lh-superiorparietal | PM₂.₅tert | 1.845 | 0.933 |
| Cortical area in mm of APARC ROI lh-superiortemporal | PM₂.₅tert | 0.548 | 0.933 |
| Cortical area in mm of APARC ROI lh-frontalpole | PM₂.₅tert | 1.441 | 0.933 |
| Cortical area in mm of APARC ROI lh-temporalpole | PM₂.₅tert | 0.962 | 0.933 |
| Cortical area in mm of APARC ROI lh-transversetemporal | PM₂.₅tert | 0.858 | 0.933 |
| Cortical area in mm of APARC ROI lh-insula | PM₂.₅tert | 0.089 | 0.933 |
| Cortical area in mm of APARC ROI rh-Banks of Superior Temporal Sulcus | PM₂.₅tert | 1.711 | 0.933 |
| Cortical area in mm of APARC ROI rh-caudalanteriorcingulate | PM₂.₅tert | 0.252 | 0.933 |
| Cortical area in mm of APARC ROI rh-caudalmiddlefrontal | PM₂.₅tert | 0.175 | 0.933 |
| Cortical area in mm of APARC ROI rh-fusiform | PM₂.₅tert | 0.752 | 0.933 |
| Cortical area in mm of APARC ROI rh-inferiorparietal | PM₂.₅tert | 1.874 | 0.933 |
| Cortical area in mm of APARC ROI rh-inferiortemporal | PM₂.₅tert | 1.189 | 0.933 |
| Cortical area in mm of APARC ROI rh-isthmuscingulate | PM₂.₅tert | 0.499 | 0.933 |
| Cortical area in mm of APARC ROI rh-lateralorbitofrontal | PM₂.₅tert | 0.573 | 0.933 |
| Cortical area in mm of APARC ROI rh-medialorbitofrontal | PM₂.₅tert | 0.270 | 0.933 |
| Cortical area in mm of APARC ROI rh-middletemporal | PM₂.₅tert | 2.038 | 0.933 |
| Cortical area in mm of APARC ROI rh-parahippocampal | PM₂.₅tert | 0.124 | 0.933 |
| Cortical area in mm of APARC ROI rh-parsopercularis | PM₂.₅tert | 0.110 | 0.933 |
| Cortical area in mm of APARC ROI rh-parsorbitalis | PM₂.₅tert | 1.157 | 0.933 |
| Cortical area in mm of APARC ROI rh-parstriangularis | PM₂.₅tert | 0.298 | 0.933 |
| Cortical area in mm of APARC ROI rh-posteriorcingulate | PM₂.₅tert | 0.467 | 0.933 |
| Cortical area in mm of APARC ROI rh-precuneus | PM₂.₅tert | 0.049 | 0.952 |
| Cortical area in mm of APARC ROI rh-rostralanteriorcingulate | PM₂.₅tert | 0.373 | 0.933 |
| Cortical area in mm of APARC ROI rh-rostralmiddlefrontal | PM₂.₅tert | 1.504 | 0.933 |
| Cortical area in mm of APARC ROI rh-superiorfrontal | PM₂.₅tert | 0.958 | 0.933 |
| Cortical area in mm of APARC ROI rh-superiorparietal | PM₂.₅tert | 0.316 | 0.933 |
| Cortical area in mm of APARC ROI rh-superiortemporal | PM₂.₅tert | 0.525 | 0.933 |
| Cortical area in mm of APARC ROI rh-frontalpole | PM₂.₅tert | 7.630 | **0.034** |
| Cortical area in mm of APARC ROI rh-temporalpole | PM₂.₅tert | 0.405 | 0.933 |
| Cortical area in mm of APARC ROI rh-transversetemporal | PM₂.₅tert | 0.896 | 0.933 |
| Cortical area in mm of APARC ROI rh-insula | PM₂.₅tert | 0.162 | 0.933 |
| Mean cortical area in mm for left hemisphere | PM₂.₅tert | 0.417 | 0.933 |
| Mean cortical area in mm for right hemisphere | PM₂.₅tert | 0.371 | 0.933 |
| Mean cortical area in mm for whole brain | PM₂.₅tert | 0.393 | 0.933 |

**Table S.14. Results from linear mixed-effects models testing the interaction effects of PM₂.₅ tertile and polygenic risk score of schizophrenia tertile on subcortical volumes including effects of population density and distance to road (fully adjusted model, sensitivity analysis).**

| **Subcortical volume** | **term** | **F** | **p-FDR** |
| --- | --- | --- | --- |
| Volume in mm³ of left-thalamus-proper | PRS-SCZtert:PM₂.₅tert | 1.165 | 0.622 |
| Volume in mm³ of left-caudate | PRS-SCZtert:PM₂.₅tert | 4.557 | **0.016** |
| Volume in mm³ of left-putamen | PRS-SCZtert:PM₂.₅tert | 2.050 | 0.242 |
| Volume in mm³ of left-pallidum | PRS-SCZtert:PM₂.₅tert | 1.099 | 0.622 |
| Volume in mm³ of left-hippocampus | PRS-SCZtert:PM₂.₅tert | 0.602 | 0.842 |
| Volume in mm³ of left-amygdala | PRS-SCZtert:PM₂.₅tert | 0.294 | 0.882 |
| Volume in mm³ of left-accumbens-area | PRS-SCZtert:PM₂.₅tert | 0.913 | 0.708 |
| Volume in mm³ of right-thalamus-proper | PRS-SCZtert:PM₂.₅tert | 2.041 | 0.242 |
| Volume in mm³ of right-caudate | PRS-SCZtert:PM₂.₅tert | 3.819 | **0.030** |
| Volume in mm³ of right-putamen | PRS-SCZtert:PM₂.₅tert | 2.928 | 0.093 |
| Volume in mm³ of right -pallidum | PRS-SCZtert:PM₂.₅tert | 0.519 | 0.842 |
| Volume in mm³ of right -hippocampus | PRS-SCZtert:PM₂.₅tert | 0.421 | 0.854 |
| Volume in mm³ of right -amygdala | PRS-SCZtert:PM₂.₅tert | 1.541 | 0.438 |
| Volume in mm³ of right -accumbens-area | PRS-SCZtert:PM₂.₅tert | 0.588 | 0.842 |

**Table S.15. Results from linear mixed-effects models testing the interaction effects of PM₂.₅ tertile and polygenic risk score of schizophrenia tertile on subcortical volumes including effects of area deprivation index (fully adjusted model, sensitivity analysis).**

| **Subcortical volume** | **term** | **F** | **p-FDR** |
| --- | --- | --- | --- |
| Volume in mm³ of left-thalamus-proper | PRS-SCZtert:PM₂.₅tert | 1.301 | 0.535 |
| Volume in mm³ of left-caudate | PRS-SCZtert:PM₂.₅tert | 4.155 | **0.033** |
| Volume in mm³ of left-putamen | PRS-SCZtert:PM₂.₅tert | 2.072 | 0.230 |
| Volume in mm³ of left-pallidum | PRS-SCZtert:PM₂.₅tert | 1.164 | 0.569 |
| Volume in mm³ of left-hippocampus | PRS-SCZtert:PM₂.₅tert | 0.489 | 0.882 |
| Volume in mm³ of left-amygdala | PRS-SCZtert:PM₂.₅tert | 0.222 | 0.926 |
| Volume in mm³ of left-accumbens-area | PRS-SCZtert:PM₂.₅tert | 0.880 | 0.739 |
| Volume in mm³ of right-thalamus-proper | PRS-SCZtert:PM₂.₅tert | 2.371 | 0.177 |
| Volume in mm³ of right-caudate | PRS-SCZtert:PM₂.₅tert | 3.249 | **0.081** |
| Volume in mm³ of right-putamen | PRS-SCZtert:PM₂.₅tert | 2.632 | 0.153 |
| Volume in mm³ of right -pallidum | PRS-SCZtert:PM₂.₅tert | 0.472 | 0.882 |
| Volume in mm³ of right -hippocampus | PRS-SCZtert:PM₂.₅tert | 0.242 | 0.926 |
| Volume in mm³ of right -amygdala | PRS-SCZtert:PM₂.₅tert | 1.404 | 0.535 |
| Volume in mm³ of right -accumbens-area | PRS-SCZtert:PM₂.₅tert | 0.713 | 0.816 |

**Table S.16. Results from linear mixed-effects models testing the effects of PM₂.₅ tertile on surface area including effects of area deprivation index (fully adjusted model, sensitivity analysis).**

| **Surface area** | **term** | **F** | **p-FDR** |
| --- | --- | --- | --- |
| Cortical area in mm of APARC ROI lh-Banks of Superior Temporal Sulcus | PM₂.₅tert | 2.425 | 0.989 |
| Cortical area in mm of APARC ROI lh-caudalanteriorcingulate | PM₂.₅tert | 0.029 | 0.989 |
| Cortical area in mm of APARC ROI lh-caudalmiddlefrontal | PM₂.₅tert | 1.008 | 0.989 |
| Cortical area in mm of APARC ROI lh-fusiform | PM₂.₅tert | 1.422 | 0.989 |
| Cortical area in mm of APARC ROI lh-inferiorparietal | PM₂.₅tert | 1.495 | 0.989 |
| Cortical area in mm of APARC ROI lh-inferiortemporal | PM₂.₅tert | 0.585 | 0.989 |
| Cortical area in mm of APARC ROI lh-isthmuscingulate | PM₂.₅tert | 0.338 | 0.989 |
| Cortical area in mm of APARC ROI lh-lateralorbitofrontal | PM₂.₅tert | 0.559 | 0.989 |
| Cortical area in mm of APARC ROI lh-medialorbitofrontal | PM₂.₅tert | 0.577 | 0.989 |
| Cortical area in mm of APARC ROI lh-middletemporal | PM₂.₅tert | 0.963 | 0.989 |
| Cortical area in mm of APARC ROI lh-parahippocampal | PM₂.₅tert | 0.488 | 0.989 |
| Cortical area in mm of APARC ROI lh-parsopercularis | PM₂.₅tert | 1.187 | 0.989 |
| Cortical area in mm of APARC ROI lh-parsorbitalis | PM₂.₅tert | 0.147 | 0.989 |
| Cortical area in mm of APARC ROI lh-parstriangularis | PM₂.₅tert | 0.507 | 0.989 |
| Cortical area in mm of APARC ROI lh-posteriorcingulate | PM₂.₅tert | 0.182 | 0.989 |
| Cortical area in mm of APARC ROI lh-precuneus | PM₂.₅tert | 0.057 | 0.989 |
| Cortical area in mm of APARC ROI lh-rostralanteriorcingulate | PM₂.₅tert | 0.202 | 0.989 |
| Cortical area in mm of APARC ROI lh-rostralmiddlefrontal | PM₂.₅tert | 0.128 | 0.989 |
| Cortical area in mm of APARC ROI lh-superiorfrontal | PM₂.₅tert | 0.062 | 0.989 |
| Cortical area in mm of APARC ROI lh-superiorparietal | PM₂.₅tert | 3.462 | 0.989 |
| Cortical area in mm of APARC ROI lh-superiortemporal | PM₂.₅tert | 0.416 | 0.989 |
| Cortical area in mm of APARC ROI lh-frontalpole | PM₂.₅tert | 1.112 | 0.989 |
| Cortical area in mm of APARC ROI lh-temporalpole | PM₂.₅tert | 0.821 | 0.989 |
| Cortical area in mm of APARC ROI lh-transversetemporal | PM₂.₅tert | 0.545 | 0.989 |
| Cortical area in mm of APARC ROI lh-insula | PM₂.₅tert | 0.069 | 0.989 |
| Cortical area in mm of APARC ROI rh-Banks of Superior Temporal Sulcus | PM₂.₅tert | 1.794 | 0.989 |
| Cortical area in mm of APARC ROI rh-caudalanteriorcingulate | PM₂.₅tert | 0.011 | 0.989 |
| Cortical area in mm of APARC ROI rh-caudalmiddlefrontal | PM₂.₅tert | 0.643 | 0.989 |
| Cortical area in mm of APARC ROI rh-fusiform | PM₂.₅tert | 0.158 | 0.989 |
| Cortical area in mm of APARC ROI rh-inferiorparietal | PM₂.₅tert | 2.206 | 0.989 |
| Cortical area in mm of APARC ROI rh-inferiortemporal | PM₂.₅tert | 1.080 | 0.989 |
| Cortical area in mm of APARC ROI rh-isthmuscingulate | PM₂.₅tert | 0.419 | 0.989 |
| Cortical area in mm of APARC ROI rh-lateralorbitofrontal | PM₂.₅tert | 0.217 | 0.989 |
| Cortical area in mm of APARC ROI rh-medialorbitofrontal | PM₂.₅tert | 0.114 | 0.989 |
| Cortical area in mm of APARC ROI rh-middletemporal | PM₂.₅tert | 2.736 | 0.989 |
| Cortical area in mm of APARC ROI rh-parahippocampal | PM₂.₅tert | 0.049 | 0.989 |
| Cortical area in mm of APARC ROI rh-parsopercularis | PM₂.₅tert | 0.027 | 0.989 |
| Cortical area in mm of APARC ROI rh-parsorbitalis | PM₂.₅tert | 0.270 | 0.989 |
| Cortical area in mm of APARC ROI rh-parstriangularis | PM₂.₅tert | 0.257 | 0.989 |
| Cortical area in mm of APARC ROI rh-posteriorcingulate | PM₂.₅tert | 0.014 | 0.989 |
| Cortical area in mm of APARC ROI rh-precuneus | PM₂.₅tert | 0.552 | 0.989 |
| Cortical area in mm of APARC ROI rh-rostralanteriorcingulate | PM₂.₅tert | 0.723 | 0.989 |
| Cortical area in mm of APARC ROI rh-rostralmiddlefrontal | PM₂.₅tert | 0.355 | 0.989 |
| Cortical area in mm of APARC ROI rh-superiorfrontal | PM₂.₅tert | 0.529 | 0.989 |
| Cortical area in mm of APARC ROI rh-superiorparietal | PM₂.₅tert | 1.000 | 0.989 |
| Cortical area in mm of APARC ROI rh-superiortemporal | PM₂.₅tert | 0.803 | 0.989 |
| Cortical area in mm of APARC ROI rh-frontalpole | PM₂.₅tert | 2.765 | **0.049** |
| Cortical area in mm of APARC ROI rh-temporalpole | PM₂.₅tert | 0.360 | 0.989 |
| Cortical area in mm of APARC ROI rh-transversetemporal | PM₂.₅tert | 0.451 | 0.989 |
| Cortical area in mm of APARC ROI rh-insula | PM₂.₅tert | 0.011 | 0.989 |
| Mean cortical area in mm for left hemisphere | PM₂.₅tert | 0.766 | 0.989 |
| Mean cortical area in mm for right hemisphere | PM₂.₅tert | 0.853 | 0.989 |
| Mean cortical area in mm for whole brain | PM₂.₅tert | 0.815 | 0.989 |

**Table S.17. Results from linear mixed-effects models testing the effects of PM₂.₅ tertile on surface area (adjusted model).**

| **Surface area** | **term** | **F** | **p-FDR** |
| --- | --- | --- | --- |
| Cortical area in mm of APARC ROI lh-Banks of Superior Temporal Sulcus | PM₂.₅tert | 3.570 | 0.766 |
| Cortical area in mm of APARC ROI lh-caudalanteriorcingulate | PM₂.₅tert | 0.258 | 0.949 |
| Cortical area in mm of APARC ROI lh-caudalmiddlefrontal | PM₂.₅tert | 0.385 | 0.949 |
| Cortical area in mm of APARC ROI lh-fusiform | PM₂.₅tert | 0.403 | 0.949 |
| Cortical area in mm of APARC ROI lh-inferiorparietal | PM₂.₅tert | 0.757 | 0.949 |
| Cortical area in mm of APARC ROI lh-inferiortemporal | PM₂.₅tert | 0.219 | 0.949 |
| Cortical area in mm of APARC ROI lh-isthmuscingulate | PM₂.₅tert | 1.009 | 0.949 |
| Cortical area in mm of APARC ROI lh-lateralorbitofrontal | PM₂.₅tert | 0.404 | 0.949 |
| Cortical area in mm of APARC ROI lh-medialorbitofrontal | PM₂.₅tert | 0.731 | 0.949 |
| Cortical area in mm of APARC ROI lh-middletemporal | PM₂.₅tert | 0.727 | 0.949 |
| Cortical area in mm of APARC ROI lh-parahippocampal | PM₂.₅tert | 0.368 | 0.949 |
| Cortical area in mm of APARC ROI lh-parsopercularis | PM₂.₅tert | 1.489 | 0.949 |
| Cortical area in mm of APARC ROI lh-parsorbitalis | PM₂.₅tert | 0.111 | 0.949 |
| Cortical area in mm of APARC ROI lh-parstriangularis | PM₂.₅tert | 0.137 | 0.949 |
| Cortical area in mm of APARC ROI lh-posteriorcingulate | PM₂.₅tert | 0.010 | 0.990 |
| Cortical area in mm of APARC ROI lh-precuneus | PM₂.₅tert | 0.217 | 0.949 |
| Cortical area in mm of APARC ROI lh-rostralanteriorcingulate | PM₂.₅tert | 0.391 | 0.949 |
| Cortical area in mm of APARC ROI lh-rostralmiddlefrontal | PM₂.₅tert | 0.776 | 0.949 |
| Cortical area in mm of APARC ROI lh-superiorfrontal | PM₂.₅tert | 0.361 | 0.949 |
| Cortical area in mm of APARC ROI lh-superiorparietal | PM₂.₅tert | 1.384 | 0.949 |
| Cortical area in mm of APARC ROI lh-superiortemporal | PM₂.₅tert | 0.105 | 0.949 |
| Cortical area in mm of APARC ROI lh-frontalpole | PM₂.₅tert | 1.399 | 0.949 |
| Cortical area in mm of APARC ROI lh-temporalpole | PM₂.₅tert | 0.952 | 0.949 |
| Cortical area in mm of APARC ROI lh-transversetemporal | PM₂.₅tert | 0.448 | 0.949 |
| Cortical area in mm of APARC ROI lh-insula | PM₂.₅tert | 0.100 | 0.949 |
| Cortical area in mm of APARC ROI rh-Banks of Superior Temporal Sulcus | PM₂.₅tert | 1.075 | 0.949 |
| Cortical area in mm of APARC ROI rh-caudalanteriorcingulate | PM₂.₅tert | 0.381 | 0.949 |
| Cortical area in mm of APARC ROI rh-caudalmiddlefrontal | PM₂.₅tert | 0.284 | 0.949 |
| Cortical area in mm of APARC ROI rh-fusiform | PM₂.₅tert | 0.244 | 0.949 |
| Cortical area in mm of APARC ROI rh-inferiorparietal | PM₂.₅tert | 1.264 | 0.949 |
| Cortical area in mm of APARC ROI rh-inferiortemporal | PM₂.₅tert | 0.277 | 0.949 |
| Cortical area in mm of APARC ROI rh-isthmuscingulate | PM₂.₅tert | 0.807 | 0.949 |
| Cortical area in mm of APARC ROI rh-lateralorbitofrontal | PM₂.₅tert | 0.310 | 0.949 |
| Cortical area in mm of APARC ROI rh-medialorbitofrontal | PM₂.₅tert | 0.965 | 0.949 |
| Cortical area in mm of APARC ROI rh-middletemporal | PM₂.₅tert | 1.249 | 0.949 |
| Cortical area in mm of APARC ROI rh-parahippocampal | PM₂.₅tert | 0.374 | 0.949 |
| Cortical area in mm of APARC ROI rh-parsopercularis | PM₂.₅tert | 0.329 | 0.949 |
| Cortical area in mm of APARC ROI rh-parsorbitalis | PM₂.₅tert | 1.008 | 0.949 |
| Cortical area in mm of APARC ROI rh-parstriangularis | PM₂.₅tert | 0.024 | 0.990 |
| Cortical area in mm of APARC ROI rh-posteriorcingulate | PM₂.₅tert | 0.705 | 0.949 |
| Cortical area in mm of APARC ROI rh-precuneus | PM₂.₅tert | 0.192 | 0.949 |
| Cortical area in mm of APARC ROI rh-rostralanteriorcingulate | PM₂.₅tert | 0.562 | 0.949 |
| Cortical area in mm of APARC ROI rh-rostralmiddlefrontal | PM₂.₅tert | 1.410 | 0.949 |
| Cortical area in mm of APARC ROI rh-superiorfrontal | PM₂.₅tert | 0.583 | 0.949 |
| Cortical area in mm of APARC ROI rh-superiorparietal | PM₂.₅tert | 0.237 | 0.949 |
| Cortical area in mm of APARC ROI rh-superiortemporal | PM₂.₅tert | 0.168 | 0.949 |
| Cortical area in mm of APARC ROI rh-frontalpole | PM₂.₅tert | 7.131 | **0.050** |
| Cortical area in mm of APARC ROI rh-temporalpole | PM₂.₅tert | 0.146 | 0.949 |
| Cortical area in mm of APARC ROI rh-transversetemporal | PM₂.₅tert | 0.491 | 0.949 |
| Cortical area in mm of APARC ROI rh-insula | PM₂.₅tert | 0.090 | 0.949 |
| Mean cortical area in mm for left hemisphere | PM₂.₅tert | 0.228 | 0.949 |
| Mean cortical area in mm for right hemisphere | PM₂.₅tert | 0.169 | 0.949 |
| Mean cortical area in mm for whole brain | PM₂.₅tert | 0.197 | 0.949 |

**Table S.18. Pairwise contrasts for the effects of PM₂.₅ tertile on resting-state surface area (adjusted model).**

| **Surface area** | **Contrast** | **Estimate** | **SE** | **p-FDR** |
| --- | --- | --- | --- | --- |
| Cortical area in mm of rh-frontal pole | PM₂.₅ Low - PM₂.₅ Medium | -2.204 | 3.401 | 0.794 |
|  | PM₂.₅ Low - PM₂.₅ High | 10.646 | 3.909 | **0.030** |
|  | PM₂.₅ Medium - PM₂.₅ High | 12.850 | 3.581 | **0.003** |

**Table S.19. Results from linear mixed-effects models testing the effects of PM₂.₅ tertile on surface area including effects of population density and distance to road (adjusted model, sensitivity analysis).**

| **Surface area** | **term** | **F** | **p-FDR** |
| --- | --- | --- | --- |
| Cortical area in mm of APARC ROI lh-Banks of Superior Temporal Sulcus | PM₂.₅tert | 3.023 | 0.965 |
| Cortical area in mm of APARC ROI lh-caudalanteriorcingulate | PM₂.₅tert | 0.512 | 0.965 |
| Cortical area in mm of APARC ROI lh-caudalmiddlefrontal | PM₂.₅tert | 0.564 | 0.965 |
| Cortical area in mm of APARC ROI lh-fusiform | PM₂.₅tert | 0.960 | 0.965 |
| Cortical area in mm of APARC ROI lh-inferiorparietal | PM₂.₅tert | 1.279 | 0.965 |
| Cortical area in mm of APARC ROI lh-inferiortemporal | PM₂.₅tert | 0.102 | 0.977 |
| Cortical area in mm of APARC ROI lh-isthmuscingulate | PM₂.₅tert | 0.647 | 0.965 |
| Cortical area in mm of APARC ROI lh-lateralorbitofrontal | PM₂.₅tert | 0.635 | 0.965 |
| Cortical area in mm of APARC ROI lh-medialorbitofrontal | PM₂.₅tert | 1.043 | 0.965 |
| Cortical area in mm of APARC ROI lh-middletemporal | PM₂.₅tert | 0.630 | 0.965 |
| Cortical area in mm of APARC ROI lh-parahippocampal | PM₂.₅tert | 0.748 | 0.965 |
| Cortical area in mm of APARC ROI lh-parsopercularis | PM₂.₅tert | 1.181 | 0.965 |
| Cortical area in mm of APARC ROI lh-parsorbitalis | PM₂.₅tert | 0.106 | 0.977 |
| Cortical area in mm of APARC ROI lh-parstriangularis | PM₂.₅tert | 0.075 | 0.980 |
| Cortical area in mm of APARC ROI lh-posteriorcingulate | PM₂.₅tert | 0.007 | 0.993 |
| Cortical area in mm of APARC ROI lh-precuneus | PM₂.₅tert | 0.244 | 0.970 |
| Cortical area in mm of APARC ROI lh-rostralanteriorcingulate | PM₂.₅tert | 0.269 | 0.970 |
| Cortical area in mm of APARC ROI lh-rostralmiddlefrontal | PM₂.₅tert | 1.497 | 0.965 |
| Cortical area in mm of APARC ROI lh-superiorfrontal | PM₂.₅tert | 0.922 | 0.965 |
| Cortical area in mm of APARC ROI lh-superiorparietal | PM₂.₅tert | 0.692 | 0.965 |
| Cortical area in mm of APARC ROI lh-superiortemporal | PM₂.₅tert | 0.531 | 0.965 |
| Cortical area in mm of APARC ROI lh-frontalpole | PM₂.₅tert | 1.526 | 0.965 |
| Cortical area in mm of APARC ROI lh-temporalpole | PM₂.₅tert | 0.797 | 0.965 |
| Cortical area in mm of APARC ROI lh-transversetemporal | PM₂.₅tert | 0.626 | 0.965 |
| Cortical area in mm of APARC ROI lh-insula | PM₂.₅tert | 0.143 | 0.977 |
| Cortical area in mm of APARC ROI rh-Banks of Superior Temporal Sulcus | PM₂.₅tert | 0.591 | 0.965 |
| Cortical area in mm of APARC ROI rh-caudalanteriorcingulate | PM₂.₅tert | 0.409 | 0.965 |
| Cortical area in mm of APARC ROI rh-caudalmiddlefrontal | PM₂.₅tert | 0.059 | 0.980 |
| Cortical area in mm of APARC ROI rh-fusiform | PM₂.₅tert | 0.645 | 0.965 |
| Cortical area in mm of APARC ROI rh-inferiorparietal | PM₂.₅tert | 0.764 | 0.965 |
| Cortical area in mm of APARC ROI rh-inferiortemporal | PM₂.₅tert | 0.546 | 0.965 |
| Cortical area in mm of APARC ROI rh-isthmuscingulate | PM₂.₅tert | 0.506 | 0.965 |
| Cortical area in mm of APARC ROI rh-lateralorbitofrontal | PM₂.₅tert | 0.716 | 0.965 |
| Cortical area in mm of APARC ROI rh-medialorbitofrontal | PM₂.₅tert | 0.883 | 0.965 |
| Cortical area in mm of APARC ROI rh-middletemporal | PM₂.₅tert | 1.132 | 0.965 |
| Cortical area in mm of APARC ROI rh-parahippocampal | PM₂.₅tert | 0.468 | 0.965 |
| Cortical area in mm of APARC ROI rh-parsopercularis | PM₂.₅tert | 0.371 | 0.965 |
| Cortical area in mm of APARC ROI rh-parsorbitalis | PM₂.₅tert | 1.559 | 0.965 |
| Cortical area in mm of APARC ROI rh-parstriangularis | PM₂.₅tert | 0.213 | 0.970 |
| Cortical area in mm of APARC ROI rh-posteriorcingulate | PM₂.₅tert | 0.480 | 0.965 |
| Cortical area in mm of APARC ROI rh-precuneus | PM₂.₅tert | 0.038 | 0.981 |
| Cortical area in mm of APARC ROI rh-rostralanteriorcingulate | PM₂.₅tert | 0.232 | 0.970 |
| Cortical area in mm of APARC ROI rh-rostralmiddlefrontal | PM₂.₅tert | 2.010 | 0.965 |
| Cortical area in mm of APARC ROI rh-superiorfrontal | PM₂.₅tert | 0.707 | 0.965 |
| Cortical area in mm of APARC ROI rh-superiorparietal | PM₂.₅tert | 0.216 | 0.970 |
| Cortical area in mm of APARC ROI rh-superiortemporal | PM₂.₅tert | 0.194 | 0.970 |
| Cortical area in mm of APARC ROI rh-frontalpole | PM₂.₅tert | 7.717 | **0.029** |
| Cortical area in mm of APARC ROI rh-temporalpole | PM₂.₅tert | 0.142 | 0.977 |
| Cortical area in mm of APARC ROI rh-transversetemporal | PM₂.₅tert | 0.949 | 0.965 |
| Cortical area in mm of APARC ROI rh-insula | PM₂.₅tert | 0.244 | 0.970 |
| Mean cortical area in mm for left hemisphere | PM₂.₅tert | 0.459 | 0.965 |
| Mean cortical area in mm for right hemisphere | PM₂.₅tert | 0.369 | 0.965 |
| Mean cortical area in mm for whole brain | PM₂.₅tert | 0.411 | 0.965 |

**Table S.20. Results from linear mixed-effects models testing the interaction effects of PM₂.₅ tertile and polygenic risk score of schizophrenia tertile on surface area (fully adjusted model).**

| **Surface area** | **term** | **F** | **p-FDR** |
| --- | --- | --- | --- |
| Cortical area in mm of APARC ROI lh-Banks of Superior Temporal Sulcus | PRS-SCZtert:PM₂.₅tert | 0.115 | 0.977 |
| Cortical area in mm of APARC ROI lh-caudalanteriorcingulate | PRS-SCZtert:PM₂.₅tert | 0.990 | 0.825 |
| Cortical area in mm of APARC ROI lh-caudalmiddlefrontal | PRS-SCZtert:PM₂.₅tert | 0.331 | 0.977 |
| Cortical area in mm of APARC ROI lh-fusiform | PRS-SCZtert:PM₂.₅tert | 0.264 | 0.977 |
| Cortical area in mm of APARC ROI lh-inferiorparietal | PRS-SCZtert:PM₂.₅tert | 0.181 | 0.977 |
| Cortical area in mm of APARC ROI lh-inferiortemporal | PRS-SCZtert:PM₂.₅tert | 0.189 | 0.977 |
| Cortical area in mm of APARC ROI lh-isthmuscingulate | PRS-SCZtert:PM₂.₅tert | 1.538 | 0.770 |
| Cortical area in mm of APARC ROI lh-lateralorbitofrontal | PRS-SCZtert:PM₂.₅tert | 1.288 | 0.825 |
| Cortical area in mm of APARC ROI lh-medialorbitofrontal | PRS-SCZtert:PM₂.₅tert | 1.600 | 0.759 |
| Cortical area in mm of APARC ROI lh-middletemporal | PRS-SCZtert:PM₂.₅tert | 0.818 | 0.825 |
| Cortical area in mm of APARC ROI lh-parahippocampal | PRS-SCZtert:PM₂.₅tert | 0.888 | 0.825 |
| Cortical area in mm of APARC ROI lh-parsopercularis | PRS-SCZtert:PM₂.₅tert | 3.235 | 0.313 |
| Cortical area in mm of APARC ROI lh-parsorbitalis | PRS-SCZtert:PM₂.₅tert | 1.798 | 0.672 |
| Cortical area in mm of APARC ROI lh-parstriangularis | PRS-SCZtert:PM₂.₅tert | 4.282 | 0.100 |
| Cortical area in mm of APARC ROI lh-posteriorcingulate | PRS-SCZtert:PM₂.₅tert | 0.334 | 0.977 |
| Cortical area in mm of APARC ROI lh-precuneus | PRS-SCZtert:PM₂.₅tert | 2.221 | 0.570 |
| Cortical area in mm of APARC ROI lh-rostralanteriorcingulate | PRS-SCZtert:PM₂.₅tert | 0.908 | 0.825 |
| Cortical area in mm of APARC ROI lh-rostralmiddlefrontal | PRS-SCZtert:PM₂.₅tert | 0.673 | 0.899 |
| Cortical area in mm of APARC ROI lh-superiorfrontal | PRS-SCZtert:PM₂.₅tert | 1.048 | 0.825 |
| Cortical area in mm of APARC ROI lh-superiorparietal | PRS-SCZtert:PM₂.₅tert | 0.578 | 0.899 |
| Cortical area in mm of APARC ROI lh-superiortemporal | PRS-SCZtert:PM₂.₅tert | 1.217 | 0.825 |
| Cortical area in mm of APARC ROI lh-frontalpole | PRS-SCZtert:PM₂.₅tert | 0.823 | 0.825 |
| Cortical area in mm of APARC ROI lh-temporalpole | PRS-SCZtert:PM₂.₅tert | 0.139 | 0.977 |
| Cortical area in mm of APARC ROI lh-transversetemporal | PRS-SCZtert:PM₂.₅tert | 2.385 | 0.524 |
| Cortical area in mm of APARC ROI lh-insula | PRS-SCZtert:PM₂.₅tert | 0.624 | 0.899 |
| Cortical area in mm of APARC ROI rh-Banks of Superior Temporal Sulcus | PRS-SCZtert:PM₂.₅tert | 2.477 | 0.524 |
| Cortical area in mm of APARC ROI rh-caudalanteriorcingulate | PRS-SCZtert:PM₂.₅tert | 1.141 | 0.825 |
| Cortical area in mm of APARC ROI rh-caudalmiddlefrontal | PRS-SCZtert:PM₂.₅tert | 0.452 | 0.977 |
| Cortical area in mm of APARC ROI rh-fusiform | PRS-SCZtert:PM₂.₅tert | 0.412 | 0.977 |
| Cortical area in mm of APARC ROI rh-inferiorparietal | PRS-SCZtert:PM₂.₅tert | 0.371 | 0.977 |
| Cortical area in mm of APARC ROI rh-inferiortemporal | PRS-SCZtert:PM₂.₅tert | 0.723 | 0.873 |
| Cortical area in mm of APARC ROI rh-isthmuscingulate | PRS-SCZtert:PM₂.₅tert | 0.788 | 0.830 |
| Cortical area in mm of APARC ROI rh-lateralorbitofrontal | PRS-SCZtert:PM₂.₅tert | 2.703 | 0.515 |
| Cortical area in mm of APARC ROI rh-medialorbitofrontal | PRS-SCZtert:PM₂.₅tert | 1.059 | 0.825 |
| Cortical area in mm of APARC ROI rh-middletemporal | PRS-SCZtert:PM₂.₅tert | 0.583 | 0.899 |
| Cortical area in mm of APARC ROI rh-parahippocampal | PRS-SCZtert:PM₂.₅tert | 0.956 | 0.825 |
| Cortical area in mm of APARC ROI rh-parsopercularis | PRS-SCZtert:PM₂.₅tert | 1.650 | 0.759 |
| Cortical area in mm of APARC ROI rh-parsorbitalis | PRS-SCZtert:PM₂.₅tert | 2.106 | 0.589 |
| Cortical area in mm of APARC ROI rh-parstriangularis | PRS-SCZtert:PM₂.₅tert | 1.958 | 0.598 |
| Cortical area in mm of APARC ROI rh-posteriorcingulate | PRS-SCZtert:PM₂.₅tert | 1.213 | 0.825 |
| Cortical area in mm of APARC ROI rh-precuneus | PRS-SCZtert:PM₂.₅tert | 1.192 | 0.825 |
| Cortical area in mm of APARC ROI rh-rostralanteriorcingulate | PRS-SCZtert:PM₂.₅tert | 1.226 | 0.825 |
| Cortical area in mm of APARC ROI rh-rostralmiddlefrontal | PRS-SCZtert:PM₂.₅tert | 0.164 | 0.977 |
| Cortical area in mm of APARC ROI rh-superiorfrontal | PRS-SCZtert:PM₂.₅tert | 0.260 | 0.977 |
| Cortical area in mm of APARC ROI rh-superiorparietal | PRS-SCZtert:PM₂.₅tert | 0.305 | 0.977 |
| Cortical area in mm of APARC ROI rh-superiortemporal | PRS-SCZtert:PM₂.₅tert | 0.890 | 0.825 |
| Cortical area in mm of APARC ROI rh-frontalpole | PRS-SCZtert:PM₂.₅tert | 1.051 | 0.825 |
| Cortical area in mm of APARC ROI rh-temporalpole | PRS-SCZtert:PM₂.₅tert | 0.590 | 0.899 |
| Cortical area in mm of APARC ROI rh-transversetemporal | PRS-SCZtert:PM₂.₅tert | 1.154 | 0.825 |
| Cortical area in mm of APARC ROI rh-insula | PRS-SCZtert:PM₂.₅tert | 1.938 | 0.598 |
| Mean cortical area in mm for left hemisphere | PRS-SCZtert:PM₂.₅tert | 0.818 | 0.825 |
| Mean cortical area in mm for right hemisphere | PRS-SCZtert:PM₂.₅tert | 0.914 | 0.825 |
| Mean cortical area in mm for whole brain | PRS-SCZtert:PM₂.₅tert | 0.870 | 0.825 |

**Table 21. Results from linear mixed-effects models testing the interaction effects of PM₂.₅** **tertile and polygenic risk score of schizophrenia tertile on subcortical volumes stratified according to manufacturer (fully adjusted model).**

| **Subcortical volume** | **term** | **F** | **p-FDR** |
| --- | --- | --- | --- |
| **GE Medical Systems** | | | |
| Volume in mm³ of left-thalamus-proper | PRS-SCZtert:PM₂.₅tert | 0.944 | 0.877 |
| Volume in mm³ of left-caudate | PRS-SCZtert:PM₂.₅tert | 2.339 | 0.587 |
| Volume in mm³ of left-putamen | PRS-SCZtert:PM₂.₅tert | 1.510 | 0.587 |
| Volume in mm³ of left-pallidum | PRS-SCZtert:PM₂.₅tert | 0.339 | 0.883 |
| Volume in mm³ of left-hippocampus | PRS-SCZtert:PM₂.₅tert | 1.883 | 0.883 |
| Volume in mm³ of left-amygdala | PRS-SCZtert:PM₂.₅tert | 0.383 | 0.883 |
| Volume in mm³ of left-accumbens-area | PRS-SCZtert:PM₂.₅tert | 0.305 | 0.883 |
| Volume in mm³ of right-thalamus-proper | PRS-SCZtert:PM₂.₅tert | 0.339 | 0.883 |
| Volume in mm³ of right-caudate | PRS-SCZtert:PM₂.₅tert | 1.791 | 0.587 |
| Volume in mm³ of right-putamen | PRS-SCZtert:PM₂.₅tert | 1.426 | 0.587 |
| Volume in mm³ of right -pallidum | PRS-SCZtert:PM₂.₅tert | 0.293 | 0.883 |
| Volume in mm³ of right -hippocampus | PRS-SCZtert:PM₂.₅tert | 0.536 | 0.883 |
| Volume in mm³ of right -amygdala | PRS-SCZtert:PM₂.₅tert | 0.694 | 0.883 |
| Volume in mm³ of right -accumbens-area | PRS-SCZtert:PM₂.₅tert | 1.348 | 0.883 |
| **Philips Medical Systems** | | | |
| Volume in mm³ of left-thalamus-proper | PRS-SCZtert:PM₂.₅tert | 2.142 | 0.144 |
| Volume in mm³ of left-caudate | PRS-SCZtert:PM₂.₅tert | 4.871 | **0.013** |
| Volume in mm³ of left-putamen | PRS-SCZtert:PM₂.₅tert | 1.931 | 0.170 |
| Volume in mm³ of left-pallidum | PRS-SCZtert:PM₂.₅tert | 1.154 | 0.426 |
| Volume in mm³ of left-hippocampus | PRS-SCZtert:PM₂.₅tert | 3.210 | 0.143 |
| Volume in mm³ of left-amygdala | PRS-SCZtert:PM₂.₅tert | 2.123 | 0.144 |
| Volume in mm³ of left-accumbens-area | PRS-SCZtert:PM₂.₅tert | 0.220 | 0.980 |
| Volume in mm³ of right-thalamus-proper | PRS-SCZtert:PM₂.₅tert | 5.303 | 0.108 |
| Volume in mm³ of right-caudate | PRS-SCZtert:PM₂.₅tert | 3.724 | **0.029** |
| Volume in mm³ of right-putamen | PRS-SCZtert:PM₂.₅tert | 0.107 | 0.068 |
| Volume in mm³ of right -pallidum | PRS-SCZtert:PM₂.₅tert | 1.864 | 0.170 |
| Volume in mm³ of right -hippocampus | PRS-SCZtert:PM₂.₅tert | 4.129 | 0.108 |
| Volume in mm³ of right -amygdala | PRS-SCZtert:PM₂.₅tert | 3.004 | 0.056 |
| Volume in mm³ of right -accumbens-area | PRS-SCZtert:PM₂.₅tert | 0.613 | 0.764 |
| **Siemens** | | | |
| Volume in mm³ of left-thalamus-proper | PRS-SCZtert:PM₂.₅tert | 2.572 | 0.586 |
| Volume in mm³ of left-caudate | PRS-SCZtert:PM₂.₅tert | 1.996 | 0.261 |
| Volume in mm³ of left-putamen | PRS-SCZtert:PM₂.₅tert | 1.820 | 0.568 |
| Volume in mm³ of left-pallidum | PRS-SCZtert:PM₂.₅tert | 1.983 | 0.586 |
| Volume in mm³ of left-hippocampus | PRS-SCZtert:PM₂.₅tert | 0.475 | 0.754 |
| Volume in mm³ of left-amygdala | PRS-SCZtert:PM₂.₅tert | 0.905 | 0.586 |
| Volume in mm³ of left-accumbens-area | PRS-SCZtert:PM₂.₅tert | 1.723 | 0.754 |
| Volume in mm³ of right-thalamus-proper | PRS-SCZtert:PM₂.₅tert | 1.852 | 0.754 |
| Volume in mm³ of right-caudate | PRS-SCZtert:PM₂.₅tert | 1.627 | 0.261 |
| Volume in mm³ of right-putamen | PRS-SCZtert:PM₂.₅tert | 2.170 | 0.261 |
| Volume in mm³ of right -pallidum | PRS-SCZtert:PM₂.₅tert | 1.552 | 0.754 |
| Volume in mm³ of right -hippocampus | PRS-SCZtert:PM₂.₅tert | 0.531 | 0.754 |
| Volume in mm³ of right -amygdala | PRS-SCZtert:PM₂.₅tert | 1.547 | 0.586 |
| Volume in mm³ of right -accumbens-area | PRS-SCZtert:PM₂.₅tert | 0.515 | 0.754 |

**Table 22. Results from linear mixed-effects models testing the interaction effects of PM₂.₅** **tertile and polygenic risk score of schizophrenia tertile on subcortical volumes stratified according to income (fully adjusted model).**

| **Subcortical volume** | **term** | **F** | **p-FDR** |
| --- | --- | --- | --- |
| **Low income** | | | |
| Volume in mm³ of left-thalamus-proper | PRS-SCZtert:PM₂.₅tert | 0.348 | 0.845 |
| Volume in mm³ of left-caudate | PRS-SCZtert:PM₂.₅tert | 1.475 | 0.438 |
| Volume in mm³ of left-putamen | PRS-SCZtert:PM₂.₅tert | 2.300 | 0.307 |
| Volume in mm³ of left-pallidum | PRS-SCZtert:PM₂.₅tert | 1.766 | 0.438 |
| Volume in mm³ of left-hippocampus | PRS-SCZtert:PM₂.₅tert | 1.451 | 0.438 |
| Volume in mm³ of left-amygdala | PRS-SCZtert:PM₂.₅tert | 1.269 | 0.497 |
| Volume in mm³ of left-accumbens-area | PRS-SCZtert:PM₂.₅tert | 1.598 | 0.438 |
| Volume in mm³ of right-thalamus-proper | PRS-SCZtert:PM₂.₅tert | 0.380 | 0.845 |
| Volume in mm³ of right-caudate | PRS-SCZtert:PM₂.₅tert | 2.251 | 0.307 |
| Volume in mm³ of right-putamen | PRS-SCZtert:PM₂.₅tert | 2.310 | 0.307 |
| Volume in mm³ of right -pallidum | PRS-SCZtert:PM₂.₅tert | 0.502 | 0.845 |
| Volume in mm³ of right -hippocampus | PRS-SCZtert:PM₂.₅tert | 0.703 | 0.827 |
| Volume in mm³ of right -amygdala | PRS-SCZtert:PM₂.₅tert | 0.982 | 0.651 |
| Volume in mm³ of right -accumbens-area | PRS-SCZtert:PM₂.₅tert | 0.559 | 0.845 |
| **Mid income** | | | |
| Volume in mm³ of left-thalamus-proper | PRS-SCZtert:PM₂.₅tert | 0.964 | 0.988 |
| Volume in mm³ of left-caudate | PRS-SCZtert:PM₂.₅tert | 1.590 | 0.911 |
| Volume in mm³ of left-putamen | PRS-SCZtert:PM₂.₅tert | 0.081 | 0.988 |
| Volume in mm³ of left-pallidum | PRS-SCZtert:PM₂.₅tert | 1.370 | 0.911 |
| Volume in mm³ of left-hippocampus | PRS-SCZtert:PM₂.₅tert | 0.572 | 0.988 |
| Volume in mm³ of left-amygdala | PRS-SCZtert:PM₂.₅tert | 0.140 | 0.988 |
| Volume in mm³ of left-accumbens-area | PRS-SCZtert:PM₂.₅tert | 0.135 | 0.988 |
| Volume in mm³ of right-thalamus-proper | PRS-SCZtert:PM₂.₅tert | 1.337 | 0.911 |
| Volume in mm³ of right-caudate | PRS-SCZtert:PM₂.₅tert | 1.323 | 0.911 |
| Volume in mm³ of right-putamen | PRS-SCZtert:PM₂.₅tert | 1.157 | 0.922 |
| Volume in mm³ of right -pallidum | PRS-SCZtert:PM₂.₅tert | 0.456 | 0.988 |
| Volume in mm³ of right -hippocampus | PRS-SCZtert:PM₂.₅tert | 0.648 | 0.988 |
| Volume in mm³ of right -amygdala | PRS-SCZtert:PM₂.₅tert | 0.423 | 0.988 |
| Volume in mm³ of right -accumbens-area | PRS-SCZtert:PM₂.₅tert | 0.209 | 0.988 |
| **High income** | | | |
| Volume in mm³ of left-thalamus-proper | PRS-SCZtert:PM₂.₅tert | 1.057 | 0.553 |
| Volume in mm³ of left-caudate | PRS-SCZtert:PM₂.₅tert | 2.853 | 0.321 |
| Volume in mm³ of left-putamen | PRS-SCZtert:PM₂.₅tert | 1.608 | 0.398 |
| Volume in mm³ of left-pallidum | PRS-SCZtert:PM₂.₅tert | 0.207 | 0.941 |
| Volume in mm³ of left-hippocampus | PRS-SCZtert:PM₂.₅tert | 1.022 | 0.553 |
| Volume in mm³ of left-amygdala | PRS-SCZtert:PM₂.₅tert | 0.195 | 0.941 |
| Volume in mm³ of left-accumbens-area | PRS-SCZtert:PM₂.₅tert | 0.872 | 0.611 |
| Volume in mm³ of right-thalamus-proper | PRS-SCZtert:PM₂.₅tert | 1.200 | 0.542 |
| Volume in mm³ of right-caudate | PRS-SCZtert:PM₂.₅tert | 1.824 | 0.347 |
| Volume in mm³ of right-putamen | PRS-SCZtert:PM₂.₅tert | 1.276 | 0.542 |
| Volume in mm³ of right -pallidum | PRS-SCZtert:PM₂.₅tert | 0.536 | 0.827 |
| Volume in mm³ of right -hippocampus | PRS-SCZtert:PM₂.₅tert | 1.996 | 0.347 |
| Volume in mm³ of right -amygdala | PRS-SCZtert:PM₂.₅tert | 2.098 | 0.347 |
| Volume in mm³ of right -accumbens-area | PRS-SCZtert:PM₂.₅tert | 1.816 | 0.347 |

**Table S.23. Results from linear mixed-effects models testing the effects of PM₂.₅ tertile on surface area stratified according to income (fully adjusted model).**

| **Surface area** | **term** | **F** | **p-FDR** |
| --- | --- | --- | --- |
| **Low income** | | | |
| Cortical area in mm of APARC ROI lh-Banks of Superior Temporal Sulcus | PM₂.₅tert | 1.720 | 0.990 |
| Cortical area in mm of APARC ROI lh-caudalanteriorcingulate | PM₂.₅tert | 0.356 | 0.990 |
| Cortical area in mm of APARC ROI lh-caudalmiddlefrontal | PM₂.₅tert | 0.117 | 0.990 |
| Cortical area in mm of APARC ROI lh-fusiform | PM₂.₅tert | 0.301 | 0.990 |
| Cortical area in mm of APARC ROI lh-inferiorparietal | PM₂.₅tert | 0.495 | 0.990 |
| Cortical area in mm of APARC ROI lh-inferiortemporal | PM₂.₅tert | 0.016 | 0.990 |
| Cortical area in mm of APARC ROI lh-isthmuscingulate | PM₂.₅tert | 0.715 | 0.990 |
| Cortical area in mm of APARC ROI lh-lateralorbitofrontal | PM₂.₅tert | 0.252 | 0.990 |
| Cortical area in mm of APARC ROI lh-medialorbitofrontal | PM₂.₅tert | 1.954 | 0.990 |
| Cortical area in mm of APARC ROI lh-middletemporal | PM₂.₅tert | 0.457 | 0.990 |
| Cortical area in mm of APARC ROI lh-parahippocampal | PM₂.₅tert | 0.935 | 0.990 |
| Cortical area in mm of APARC ROI lh-parsopercularis | PM₂.₅tert | 0.393 | 0.990 |
| Cortical area in mm of APARC ROI lh-parsorbitalis | PM₂.₅tert | 0.253 | 0.990 |
| Cortical area in mm of APARC ROI lh-parstriangularis | PM₂.₅tert | 0.912 | 0.990 |
| Cortical area in mm of APARC ROI lh-posteriorcingulate | PM₂.₅tert | 0.978 | 0.990 |
| Cortical area in mm of APARC ROI lh-precuneus | PM₂.₅tert | 0.127 | 0.990 |
| Cortical area in mm of APARC ROI lh-rostralanteriorcingulate | PM₂.₅tert | 0.528 | 0.990 |
| Cortical area in mm of APARC ROI lh-rostralmiddlefrontal | PM₂.₅tert | 1.017 | 0.990 |
| Cortical area in mm of APARC ROI lh-superiorfrontal | PM₂.₅tert | 0.373 | 0.990 |
| Cortical area in mm of APARC ROI lh-superiorparietal | PM₂.₅tert | 1.035 | 0.990 |
| Cortical area in mm of APARC ROI lh-superiortemporal | PM₂.₅tert | 0.115 | 0.990 |
| Cortical area in mm of APARC ROI lh-frontalpole | PM₂.₅tert | 1.093 | 0.990 |
| Cortical area in mm of APARC ROI lh-temporalpole | PM₂.₅tert | 1.499 | 0.990 |
| Cortical area in mm of APARC ROI lh-transversetemporal | PM₂.₅tert | 0.412 | 0.990 |
| Cortical area in mm of APARC ROI lh-insula | PM₂.₅tert | 1.006 | 0.990 |
| Cortical area in mm of APARC ROI rh-Banks of Superior Temporal Sulcus | PM₂.₅tert | 0.727 | 0.990 |
| Cortical area in mm of APARC ROI rh-caudalanteriorcingulate | PM₂.₅tert | 0.482 | 0.990 |
| Cortical area in mm of APARC ROI rh-caudalmiddlefrontal | PM₂.₅tert | 2.062 | 0.990 |
| Cortical area in mm of APARC ROI rh-fusiform | PM₂.₅tert | 0.167 | 0.990 |
| Cortical area in mm of APARC ROI rh-inferiorparietal | PM₂.₅tert | 0.595 | 0.990 |
| Cortical area in mm of APARC ROI rh-inferiortemporal | PM₂.₅tert | 0.051 | 0.990 |
| Cortical area in mm of APARC ROI rh-isthmuscingulate | PM₂.₅tert | 0.379 | 0.990 |
| Cortical area in mm of APARC ROI rh-lateralorbitofrontal | PM₂.₅tert | 0.684 | 0.990 |
| Cortical area in mm of APARC ROI rh-medialorbitofrontal | PM₂.₅tert | 0.094 | 0.990 |
| Cortical area in mm of APARC ROI rh-middletemporal | PM₂.₅tert | 0.028 | 0.990 |
| Cortical area in mm of APARC ROI rh-parahippocampal | PM₂.₅tert | 0.740 | 0.990 |
| Cortical area in mm of APARC ROI rh-parsopercularis | PM₂.₅tert | 0.260 | 0.990 |
| Cortical area in mm of APARC ROI rh-parsorbitalis | PM₂.₅tert | 0.178 | 0.990 |
| Cortical area in mm of APARC ROI rh-parstriangularis | PM₂.₅tert | 0.321 | 0.990 |
| Cortical area in mm of APARC ROI rh-posteriorcingulate | PM₂.₅tert | 0.092 | 0.990 |
| Cortical area in mm of APARC ROI rh-precuneus | PM₂.₅tert | 0.344 | 0.990 |
| Cortical area in mm of APARC ROI rh-rostralanteriorcingulate | PM₂.₅tert | 0.010 | 0.990 |
| Cortical area in mm of APARC ROI rh-rostralmiddlefrontal | PM₂.₅tert | 0.218 | 0.990 |
| Cortical area in mm of APARC ROI rh-superiorfrontal | PM₂.₅tert | 1.613 | 0.990 |
| Cortical area in mm of APARC ROI rh-superiorparietal | PM₂.₅tert | 1.488 | 0.990 |
| Cortical area in mm of APARC ROI rh-superiortemporal | PM₂.₅tert | 0.086 | 0.990 |
| Cortical area in mm of APARC ROI rh-frontalpole | PM₂.₅tert | 1.582 | 0.990 |
| Cortical area in mm of APARC ROI rh-temporalpole | PM₂.₅tert | 2.523 | 0.990 |
| Cortical area in mm of APARC ROI rh-transversetemporal | PM₂.₅tert | 1.531 | 0.990 |
| Cortical area in mm of APARC ROI rh-insula | PM₂.₅tert | 0.452 | 0.990 |
| Mean cortical area in mm for left hemisphere | PM₂.₅tert | 0.362 | 0.990 |
| Mean cortical area in mm for right hemisphere | PM₂.₅tert | 0.381 | 0.990 |
| Mean cortical area in mm for whole brain | PM₂.₅tert | 0.371 | 0.990 |
| **Middle income** | | | |
| Cortical area in mm of APARC ROI lh-Banks of Superior Temporal Sulcus | PM₂.₅tert | 2.240 | 0.647 |
| Cortical area in mm of APARC ROI lh-caudalanteriorcingulate | PM₂.₅tert | 0.635 | 0.778 |
| Cortical area in mm of APARC ROI lh-caudalmiddlefrontal | PM₂.₅tert | 1.314 | 0.692 |
| Cortical area in mm of APARC ROI lh-fusiform | PM₂.₅tert | 1.964 | 0.647 |
| Cortical area in mm of APARC ROI lh-inferiorparietal | PM₂.₅tert | 1.722 | 0.647 |
| Cortical area in mm of APARC ROI lh-inferiortemporal | PM₂.₅tert | 0.515 | 0.807 |
| Cortical area in mm of APARC ROI lh-isthmuscingulate | PM₂.₅tert | 0.152 | 0.929 |
| Cortical area in mm of APARC ROI lh-lateralorbitofrontal | PM₂.₅tert | 1.794 | 0.647 |
| Cortical area in mm of APARC ROI lh-medialorbitofrontal | PM₂.₅tert | 0.376 | 0.847 |
| Cortical area in mm of APARC ROI lh-middletemporal | PM₂.₅tert | 1.501 | 0.663 |
| Cortical area in mm of APARC ROI lh-parahippocampal | PM₂.₅tert | 0.486 | 0.807 |
| Cortical area in mm of APARC ROI lh-parsopercularis | PM₂.₅tert | 1.084 | 0.706 |
| Cortical area in mm of APARC ROI lh-parsorbitalis | PM₂.₅tert | 0.696 | 0.778 |
| Cortical area in mm of APARC ROI lh-parstriangularis | PM₂.₅tert | 0.486 | 0.807 |
| Cortical area in mm of APARC ROI lh-posteriorcingulate | PM₂.₅tert | 0.613 | 0.778 |
| Cortical area in mm of APARC ROI lh-precuneus | PM₂.₅tert | 0.103 | 0.938 |
| Cortical area in mm of APARC ROI lh-rostralanteriorcingulate | PM₂.₅tert | 1.012 | 0.706 |
| Cortical area in mm of APARC ROI lh-rostralmiddlefrontal | PM₂.₅tert | 0.618 | 0.778 |
| Cortical area in mm of APARC ROI lh-superiorfrontal | PM₂.₅tert | 1.976 | 0.647 |
| Cortical area in mm of APARC ROI lh-superiorparietal | PM₂.₅tert | 1.882 | 0.647 |
| Cortical area in mm of APARC ROI lh-superiortemporal | PM₂.₅tert | 4.331 | 0.647 |
| Cortical area in mm of APARC ROI lh-frontalpole | PM₂.₅tert | 2.112 | 0.647 |
| Cortical area in mm of APARC ROI lh-temporalpole | PM₂.₅tert | 0.006 | 0.994 |
| Cortical area in mm of APARC ROI lh-transversetemporal | PM₂.₅tert | 1.064 | 0.706 |
| Cortical area in mm of APARC ROI lh-insula | PM₂.₅tert | 1.608 | 0.647 |
| Cortical area in mm of APARC ROI rh-Banks of Superior Temporal Sulcus | PM₂.₅tert | 0.309 | 0.859 |
| Cortical area in mm of APARC ROI rh-caudalanteriorcingulate | PM₂.₅tert | 1.045 | 0.706 |
| Cortical area in mm of APARC ROI rh-caudalmiddlefrontal | PM₂.₅tert | 0.126 | 0.934 |
| Cortical area in mm of APARC ROI rh-fusiform | PM₂.₅tert | 0.353 | 0.847 |
| Cortical area in mm of APARC ROI rh-inferiorparietal | PM₂.₅tert | 2.263 | 0.647 |
| Cortical area in mm of APARC ROI rh-inferiortemporal | PM₂.₅tert | 1.165 | 0.706 |
| Cortical area in mm of APARC ROI rh-isthmuscingulate | PM₂.₅tert | 0.163 | 0.929 |
| Cortical area in mm of APARC ROI rh-lateralorbitofrontal | PM₂.₅tert | 2.270 | 0.647 |
| Cortical area in mm of APARC ROI rh-medialorbitofrontal | PM₂.₅tert | 0.819 | 0.735 |
| Cortical area in mm of APARC ROI rh-middletemporal | PM₂.₅tert | 2.977 | 0.647 |
| Cortical area in mm of APARC ROI rh-parahippocampal | PM₂.₅tert | 1.362 | 0.688 |
| Cortical area in mm of APARC ROI rh-parsopercularis | PM₂.₅tert | 0.691 | 0.778 |
| Cortical area in mm of APARC ROI rh-parsorbitalis | PM₂.₅tert | 1.964 | 0.647 |
| Cortical area in mm of APARC ROI rh-parstriangularis | PM₂.₅tert | 0.353 | 0.847 |
| Cortical area in mm of APARC ROI rh-posteriorcingulate | PM₂.₅tert | 0.273 | 0.859 |
| Cortical area in mm of APARC ROI rh-precuneus | PM₂.₅tert | 0.473 | 0.807 |
| Cortical area in mm of APARC ROI rh-rostralanteriorcingulate | PM₂.₅tert | 0.016 | 0.994 |
| Cortical area in mm of APARC ROI rh-rostralmiddlefrontal | PM₂.₅tert | 1.893 | 0.647 |
| Cortical area in mm of APARC ROI rh-superiorfrontal | PM₂.₅tert | 1.609 | 0.647 |
| Cortical area in mm of APARC ROI rh-superiorparietal | PM₂.₅tert | 1.443 | 0.663 |
| Cortical area in mm of APARC ROI rh-superiortemporal | PM₂.₅tert | 1.830 | 0.647 |
| Cortical area in mm of APARC ROI rh-frontalpole | PM₂.₅tert | 1.081 | 0.706 |
| Cortical area in mm of APARC ROI rh-temporalpole | PM₂.₅tert | 0.292 | 0.859 |
| Cortical area in mm of APARC ROI rh-transversetemporal | PM₂.₅tert | 2.429 | 0.647 |
| Cortical area in mm of APARC ROI rh-insula | PM₂.₅tert | 0.989 | 0.706 |
| Mean cortical area in mm for left hemisphere | PM₂.₅tert | 0.824 | 0.735 |
| Mean cortical area in mm for right hemisphere | PM₂.₅tert | 0.906 | 0.735 |
| Mean cortical area in mm for whole brain | PM₂.₅tert | 0.867 | 0.735 |
| **High income** | | | |
| Cortical area in mm of APARC ROI lh-Banks of Superior Temporal Sulcus | PM₂.₅tert | 0.925 | 0.984 |
| Cortical area in mm of APARC ROI lh-caudalanteriorcingulate | PM₂.₅tert | 0.448 | 0.984 |
| Cortical area in mm of APARC ROI lh-caudalmiddlefrontal | PM₂.₅tert | 0.355 | 0.984 |
| Cortical area in mm of APARC ROI lh-fusiform | PM₂.₅tert | 0.772 | 0.984 |
| Cortical area in mm of APARC ROI lh-inferiorparietal | PM₂.₅tert | 0.227 | 0.984 |
| Cortical area in mm of APARC ROI lh-inferiortemporal | PM₂.₅tert | 0.085 | 0.984 |
| Cortical area in mm of APARC ROI lh-isthmuscingulate | PM₂.₅tert | 2.229 | 0.984 |
| Cortical area in mm of APARC ROI lh-lateralorbitofrontal | PM₂.₅tert | 0.125 | 0.984 |
| Cortical area in mm of APARC ROI lh-medialorbitofrontal | PM₂.₅tert | 0.275 | 0.984 |
| Cortical area in mm of APARC ROI lh-middletemporal | PM₂.₅tert | 0.033 | 0.984 |
| Cortical area in mm of APARC ROI lh-parahippocampal | PM₂.₅tert | 1.067 | 0.984 |
| Cortical area in mm of APARC ROI lh-parsopercularis | PM₂.₅tert | 0.578 | 0.984 |
| Cortical area in mm of APARC ROI lh-parsorbitalis | PM₂.₅tert | 0.043 | 0.984 |
| Cortical area in mm of APARC ROI lh-parstriangularis | PM₂.₅tert | 0.035 | 0.984 |
| Cortical area in mm of APARC ROI lh-posteriorcingulate | PM₂.₅tert | 0.224 | 0.984 |
| Cortical area in mm of APARC ROI lh-precuneus | PM₂.₅tert | 0.093 | 0.984 |
| Cortical area in mm of APARC ROI lh-rostralanteriorcingulate | PM₂.₅tert | 0.346 | 0.984 |
| Cortical area in mm of APARC ROI lh-rostralmiddlefrontal | PM₂.₅tert | 1.155 | 0.984 |
| Cortical area in mm of APARC ROI lh-superiorfrontal | PM₂.₅tert | 0.601 | 0.984 |
| Cortical area in mm of APARC ROI lh-superiorparietal | PM₂.₅tert | 0.803 | 0.984 |
| Cortical area in mm of APARC ROI lh-superiortemporal | PM₂.₅tert | 0.871 | 0.984 |
| Cortical area in mm of APARC ROI lh-frontalpole | PM₂.₅tert | 0.921 | 0.984 |
| Cortical area in mm of APARC ROI lh-temporalpole | PM₂.₅tert | 0.697 | 0.984 |
| Cortical area in mm of APARC ROI lh-transversetemporal | PM₂.₅tert | 0.137 | 0.984 |
| Cortical area in mm of APARC ROI lh-insula | PM₂.₅tert | 2.383 | 0.984 |
| Cortical area in mm of APARC ROI rh-Banks of Superior Temporal Sulcus | PM₂.₅tert | 0.478 | 0.984 |
| Cortical area in mm of APARC ROI rh-caudalanteriorcingulate | PM₂.₅tert | 0.277 | 0.984 |
| Cortical area in mm of APARC ROI rh-caudalmiddlefrontal | PM₂.₅tert | 0.598 | 0.984 |
| Cortical area in mm of APARC ROI rh-fusiform | PM₂.₅tert | 0.415 | 0.984 |
| Cortical area in mm of APARC ROI rh-inferiorparietal | PM₂.₅tert | 0.461 | 0.984 |
| Cortical area in mm of APARC ROI rh-inferiortemporal | PM₂.₅tert | 0.124 | 0.984 |
| Cortical area in mm of APARC ROI rh-isthmuscingulate | PM₂.₅tert | 1.518 | 0.984 |
| Cortical area in mm of APARC ROI rh-lateralorbitofrontal | PM₂.₅tert | 0.367 | 0.984 |
| Cortical area in mm of APARC ROI rh-medialorbitofrontal | PM₂.₅tert | 0.420 | 0.984 |
| Cortical area in mm of APARC ROI rh-middletemporal | PM₂.₅tert | 0.373 | 0.984 |
| Cortical area in mm of APARC ROI rh-parahippocampal | PM₂.₅tert | 0.016 | 0.984 |
| Cortical area in mm of APARC ROI rh-parsopercularis | PM₂.₅tert | 0.069 | 0.984 |
| Cortical area in mm of APARC ROI rh-parsorbitalis | PM₂.₅tert | 0.220 | 0.984 |
| Cortical area in mm of APARC ROI rh-parstriangularis | PM₂.₅tert | 1.442 | 0.984 |
| Cortical area in mm of APARC ROI rh-posteriorcingulate | PM₂.₅tert | 0.651 | 0.984 |
| Cortical area in mm of APARC ROI rh-precuneus | PM₂.₅tert | 0.225 | 0.984 |
| Cortical area in mm of APARC ROI rh-rostralanteriorcingulate | PM₂.₅tert | 0.933 | 0.984 |
| Cortical area in mm of APARC ROI rh-rostralmiddlefrontal | PM₂.₅tert | 0.682 | 0.984 |
| Cortical area in mm of APARC ROI rh-superiorfrontal | PM₂.₅tert | 0.068 | 0.984 |
| Cortical area in mm of APARC ROI rh-superiorparietal | PM₂.₅tert | 0.446 | 0.984 |
| Cortical area in mm of APARC ROI rh-superiortemporal | PM₂.₅tert | 0.291 | 0.984 |
| Cortical area in mm of APARC ROI rh-frontalpole | PM₂.₅tert | 4.877 | 0.418 |
| Cortical area in mm of APARC ROI rh-temporalpole | PM₂.₅tert | 0.645 | 0.984 |
| Cortical area in mm of APARC ROI rh-transversetemporal | PM₂.₅tert | 0.639 | 0.984 |
| Cortical area in mm of APARC ROI rh-insula | PM₂.₅tert | 0.407 | 0.984 |
| Mean cortical area in mm for left hemisphere | PM₂.₅tert | 0.080 | 0.984 |
| Mean cortical area in mm for right hemisphere | PM₂.₅tert | 0.088 | 0.984 |
| Mean cortical area in mm for whole brain | PM₂.₅tert | 0.085 | 0.984 |

**Table S.24. Results from linear mixed-effects models testing the effects of NO₂ tertile on resting-state functional connectivity (fully adjusted model).**

| **Resting-state fMRI connectivity** | **term** | **F** | **p-FDR** |
| --- | --- | --- | --- |
| Resting-state fMRI connectivity between Cingulate Gyrus and Cingulate Gyrus | NO₂tert | 0.090 | 0.946 |
| Resting-state fMRI connectivity between Cingulate Gyrus and CinguloParietal | NO₂tert | 0.971 | 0.737 |
| Resting-state fMRI connectivity between Cingulate Gyrus and Default Mode Network | NO₂tert | 3.706 | 0.152 |
| Resting-state fMRI connectivity between Cingulate Gyrus and Dorsal Attention Network | NO₂tert | 0.366 | 0.924 |
| Resting-state fMRI connectivity between Cingulate Gyrus and Frontoparietal Network | NO₂tert | 3.096 | 0.171 |
| Resting-state fMRI connectivity between Cingulate Gyrus and Salience Network | NO₂tert | 0.205 | 0.945 |
| Resting-state fMRI connectivity between Cingulate Gyrus and Ventral Attention Network | NO₂tert | 0.278 | 0.924 |
| Resting-state fMRI connectivity between CinguloParietal and CinguloParietal | NO₂tert | 0.097 | 0.946 |
| Resting-state fMRI connectivity between CinguloParietal and Default Mode Network | NO₂tert | 3.046 | 0.171 |
| Resting-state fMRI connectivity between CinguloParietal and Dorsal Attention Network | NO₂tert | 0.537 | 0.894 |
| Resting-state fMRI connectivity between CinguloParietal and Frontoparietal Network | NO₂tert | 0.270 | 0.924 |
| Resting-state fMRI connectivity between CinguloParietal and Salience Network | NO₂tert | 0.361 | 0.924 |
| Resting-state fMRI connectivity between CinguloParietal and Ventral Attention Network | NO₂tert | 0.722 | 0.883 |
| Resting-state fMRI connectivity between Default Mode Network and Default Mode Network | NO₂tert | 1.565 | 0.471 |
| Resting-state fMRI connectivity between Default Mode Network and Dorsal Attention Network | NO₂tert | 5.541 | **0.043** |
| Resting-state fMRI connectivity between Default Mode Network and Frontoparietal Network | NO₂tert | 2.097 | 0.332 |
| Resting-state fMRI connectivity between Default Mode Network and Salience Network | NO₂tert | 0.338 | 0.924 |
| Resting-state fMRI connectivity between Default Mode Network and Ventral Attention Network | NO₂tert | 1.502 | 0.471 |
| Resting-state fMRI connectivity between Dorsal Attention Network and Dorsal Attention Network | NO₂tert | 2.789 | 0.185 |
| Resting-state fMRI connectivity between Dorsal Attention Network and Frontoparietal Network | NO₂tert | 6.312 | **0.043** |
| Resting-state fMRI connectivity between Dorsal Attention Network and Salience Network | NO₂tert | 3.751 | 0.152 |
| Resting-state fMRI connectivity between Dorsal Attention Network and Ventral Attention Network | NO₂tert | 0.555 | 0.894 |
| Resting-state fMRI connectivity between Frontoparietal Network and Frontoparietal Network | NO₂tert | 4.343 | 0.128 |
| Resting-state fMRI connectivity between Frontoparietal Network and Salience Network | NO₂tert | 1.876 | 0.378 |
| Resting-state fMRI connectivity between Frontoparietal Network and Ventral Attention Network | NO₂tert | 0.037 | 0.963 |
| Resting-state fMRI connectivity between Salience Network and Salience Network | NO₂tert | 0.575 | 0.894 |
| Resting-state fMRI connectivity between Salience Network and Visual Network | NO₂tert | 0.103 | 0.946 |
| Resting-state fMRI connectivity between Ventral Attention Network and Ventral Attention Network | NO₂tert | 3.573 | 0.171 |

**Table S.25. Pairwise contrasts for the effects of NO₂ tertile on resting-state functional connectivity (fully adjusted model).**

| **Resting-state fMRI connectivity** | **Contrast** | **Estimate** | **SE** | **p-FDR** |
| --- | --- | --- | --- | --- |
| Resting-state fMRI connectivity between Default Mode Network and Dorsal Attention Network | NO₂ Low - NO₂ Medium | 0.005 | 0.004 | 0.263 |
|  | NO₂ Low - NO₂ High | 0.016 | 0.005 | **0.002** |
|  | NO₂ Medium - NO₂ High | 0.011 | 0.004 | **0.017** |
| Resting-state fMRI connectivity between Dorsal Attention Network and Frontoparietal Network | NO₂ Low - NO₂ Medium | 0.011 | 0.003 | **0.005** |
|  | NO₂ Low - NO₂ High | 0.009 | 0.004 | **0.017** |
|  | NO₂ Medium - NO₂ High | -0.002 | 0.003 | 0.634 |

**Table S.26. Results from linear mixed-effects models testing the interaction effects of NO₂ tertile and polygenic risk score of schizophrenia tertile on resting-state functional connectivity (fully adjusted model).**

| **Resting-state fMRI connectivity** | **term** | **F** | **p-FDR** |
| --- | --- | --- | --- |
| Resting-state fMRI connectivity between Cingulate Gyrus and Cingulate Gyrus | PRS-SCZtert:NO₂tert | 1.345 | 0.866 |
| Resting-state fMRI connectivity between Cingulate Gyrus and CinguloParietal | PRS-SCZtert:NO₂tert | 0.887 | 0.866 |
| Resting-state fMRI connectivity between Cingulate Gyrus and Default Mode Network | PRS-SCZtert:NO₂tert | 2.109 | 0.866 |
| Resting-state fMRI connectivity between Cingulate Gyrus and Dorsal Attention Network | PRS-SCZtert:NO₂tert | 0.516 | 0.922 |
| Resting-state fMRI connectivity between Cingulate Gyrus and Frontoparietal Network | PRS-SCZtert:NO₂tert | 0.169 | 0.954 |
| Resting-state fMRI connectivity between Cingulate Gyrus and Salience Network | PRS-SCZtert:NO₂tert | 0.668 | 0.905 |
| Resting-state fMRI connectivity between Cingulate Gyrus and Ventral Attention Network | PRS-SCZtert:NO₂tert | 0.868 | 0.866 |
| Resting-state fMRI connectivity between CinguloParietal and CinguloParietal | PRS-SCZtert:NO₂tert | 2.534 | 0.866 |
| Resting-state fMRI connectivity between CinguloParietal and Default Mode Network | PRS-SCZtert:NO₂tert | 0.959 | 0.866 |
| Resting-state fMRI connectivity between CinguloParietal and Dorsal Attention Network | PRS-SCZtert:NO₂tert | 0.694 | 0.905 |
| Resting-state fMRI connectivity between CinguloParietal and Frontoparietal Network | PRS-SCZtert:NO₂tert | 1.218 | 0.866 |
| Resting-state fMRI connectivity between CinguloParietal and Salience Network | PRS-SCZtert:NO₂tert | 1.948 | 0.866 |
| Resting-state fMRI connectivity between CinguloParietal and Ventral Attention Network | PRS-SCZtert:NO₂tert | 0.342 | 0.923 |
| Resting-state fMRI connectivity between Default Mode Network and Default Mode Network | PRS-SCZtert:NO₂tert | 0.848 | 0.866 |
| Resting-state fMRI connectivity between Default Mode Network and Dorsal Attention Network | PRS-SCZtert:NO₂tert | 1.434 | 0.866 |
| Resting-state fMRI connectivity between Default Mode Network and Frontoparietal Network | PRS-SCZtert:NO₂tert | 1.338 | 0.866 |
| Resting-state fMRI connectivity between Default Mode Network and Salience Network | PRS-SCZtert:NO₂tert | 0.691 | 0.905 |
| Resting-state fMRI connectivity between Default Mode Network and Ventral Attention Network | PRS-SCZtert:NO₂tert | 0.353 | 0.923 |
| Resting-state fMRI connectivity between Dorsal Attention Network and Dorsal Attention Network | PRS-SCZtert:NO₂tert | 0.939 | 0.866 |
| Resting-state fMRI connectivity between Dorsal Attention Network and Frontoparietal Network | PRS-SCZtert:NO₂tert | 0.306 | 0.923 |
| Resting-state fMRI connectivity between Dorsal Attention Network and Salience Network | PRS-SCZtert:NO₂tert | 0.281 | 0.923 |
| Resting-state fMRI connectivity between Dorsal Attention Network and Ventral Attention Network | PRS-SCZtert:NO₂tert | 0.531 | 0.922 |
| Resting-state fMRI connectivity between Frontoparietal Network and Frontoparietal Network | PRS-SCZtert:NO₂tert | 1.530 | 0.866 |
| Resting-state fMRI connectivity between Frontoparietal Network and Salience Network | PRS-SCZtert:NO₂tert | 0.356 | 0.923 |
| Resting-state fMRI connectivity between Frontoparietal Network and Ventral Attention Network | PRS-SCZtert:NO₂tert | 1.272 | 0.866 |
| Resting-state fMRI connectivity between Salience Network and Salience Network | PRS-SCZtert:NO₂tert | 0.875 | 0.866 |
| Resting-state fMRI connectivity between Salience Network and Visual Network | PRS-SCZtert:NO₂tert | 0.520 | 0.922 |
| Resting-state fMRI connectivity between Ventral Attention Network and Ventral Attention Network | PRS-SCZtert:NO₂tert | 0.959 | 0.866 |

**Table S.27. Results from linear mixed-effects models testing the effects of NO₂ tertile on resting-state functional connectivity including effects of population density and distance to road (fully adjusted model, sensitivity analysis).**

| **Resting-state fMRI connectivity** | **term** | **F** | **p-FDR** |
| --- | --- | --- | --- |
| Resting-state fMRI connectivity between Cingulate Gyrus and Cingulate Gyrus | NO₂tert | 0.268 | 0.899 |
| Resting-state fMRI connectivity between Cingulate Gyrus and CinguloParietal | NO₂tert | 1.156 | 0.634 |
| Resting-state fMRI connectivity between Cingulate Gyrus and Default Mode Network | NO₂tert | 3.364 | 0.166 |
| Resting-state fMRI connectivity between Cingulate Gyrus and Dorsal Attention Network | NO₂tert | 0.532 | 0.899 |
| Resting-state fMRI connectivity between Cingulate Gyrus and Frontoparietal Network | NO₂tert | 3.449 | 0.166 |
| Resting-state fMRI connectivity between Cingulate Gyrus and Salience Network | NO₂tert | 0.178 | 0.899 |
| Resting-state fMRI connectivity between Cingulate Gyrus and Ventral Attention Network | NO₂tert | 0.260 | 0.899 |
| Resting-state fMRI connectivity between CinguloParietal and CinguloParietal | NO₂tert | 0.143 | 0.899 |
| Resting-state fMRI connectivity between CinguloParietal and Default Mode Network | NO₂tert | 3.110 | 0.174 |
| Resting-state fMRI connectivity between CinguloParietal and Dorsal Attention Network | NO₂tert | 0.556 | 0.899 |
| Resting-state fMRI connectivity between CinguloParietal and Frontoparietal Network | NO₂tert | 0.289 | 0.899 |
| Resting-state fMRI connectivity between CinguloParietal and Salience Network | NO₂tert | 0.382 | 0.899 |
| Resting-state fMRI connectivity between CinguloParietal and Ventral Attention Network | NO₂tert | 0.995 | 0.695 |
| Resting-state fMRI connectivity between Default Mode Network and Default Mode Network | NO₂tert | 1.491 | 0.489 |
| Resting-state fMRI connectivity between Default Mode Network and Dorsal Attention Network | NO₂tert | 6.131 | **0.034** |
| Resting-state fMRI connectivity between Default Mode Network and Frontoparietal Network | NO₂tert | 2.220 | 0.313 |
| Resting-state fMRI connectivity between Default Mode Network and Salience Network | NO₂tert | 0.259 | 0.899 |
| Resting-state fMRI connectivity between Default Mode Network and Ventral Attention Network | NO₂tert | 1.516 | 0.489 |
| Resting-state fMRI connectivity between Dorsal Attention Network and Dorsal Attention Network | NO₂tert | 3.044 | 0.174 |
| Resting-state fMRI connectivity between Dorsal Attention Network and Frontoparietal Network | NO₂tert | 6.885 | **0.034** |
| Resting-state fMRI connectivity between Dorsal Attention Network and Salience Network | NO₂tert | 4.103 | 0.165 |
| Resting-state fMRI connectivity between Dorsal Attention Network and Ventral Attention Network | NO₂tert | 0.768 | 0.812 |
| Resting-state fMRI connectivity between Frontoparietal Network and Frontoparietal Network | NO₂tert | 3.408 | 0.166 |
| Resting-state fMRI connectivity between Frontoparietal Network and Salience Network | NO₂tert | 1.700 | 0.474 |
| Resting-state fMRI connectivity between Frontoparietal Network and Ventral Attention Network | NO₂tert | 0.069 | 0.934 |
| Resting-state fMRI connectivity between Salience Network and Salience Network | NO₂tert | 0.451 | 0.899 |
| Resting-state fMRI connectivity between Salience Network and Visual Network | NO₂tert | 0.193 | 0.899 |
| Resting-state fMRI connectivity between Ventral Attention Network and Ventral Attention Network | NO₂tert | 2.657 | 0.220 |

**Table S.28. Results from linear mixed-effects models testing the effects of NO₂ tertile on resting-state functional connectivity including effects of area deprivation index (fully adjusted model, sensitivity analysis).**

| **Resting-state fMRI connectivity** | **term** | **F** | **p-FDR** |
| --- | --- | --- | --- |
| Resting-state fMRI connectivity between Cingulate Gyrus and Cingulate Gyrus | NO₂tert | 0.491 | 0.910 |
| Resting-state fMRI connectivity between Cingulate Gyrus and CinguloParietal | NO₂tert | 0.380 | 0.913 |
| Resting-state fMRI connectivity between Cingulate Gyrus and Default Mode Network | NO₂tert | 3.319 | 0.260 |
| Resting-state fMRI connectivity between Cingulate Gyrus and Dorsal Attention Network | NO₂tert | 0.197 | 0.954 |
| Resting-state fMRI connectivity between Cingulate Gyrus and Frontoparietal Network | NO₂tert | 5.064 | 0.074 |
| Resting-state fMRI connectivity between Cingulate Gyrus and Salience Network | NO₂tert | 0.041 | 0.965 |
| Resting-state fMRI connectivity between Cingulate Gyrus and Ventral Attention Network | NO₂tert | 0.482 | 0.910 |
| Resting-state fMRI connectivity between CinguloParietal and CinguloParietal | NO₂tert | 0.142 | 0.954 |
| Resting-state fMRI connectivity between CinguloParietal and Default Mode Network | NO₂tert | 1.701 | 0.515 |
| Resting-state fMRI connectivity between CinguloParietal and Dorsal Attention Network | NO₂tert | 0.539 | 0.910 |
| Resting-state fMRI connectivity between CinguloParietal and Frontoparietal Network | NO₂tert | 0.121 | 0.954 |
| Resting-state fMRI connectivity between CinguloParietal and Salience Network | NO₂tert | 0.035 | 0.965 |
| Resting-state fMRI connectivity between CinguloParietal and Ventral Attention Network | NO₂tert | 1.147 | 0.723 |
| Resting-state fMRI connectivity between Default Mode Network and Default Mode Network | NO₂tert | 0.977 | 0.756 |
| Resting-state fMRI connectivity between Default Mode Network and Dorsal Attention Network | NO₂tert | 4.919 | **0.074** |
| Resting-state fMRI connectivity between Default Mode Network and Frontoparietal Network | NO₂tert | 1.096 | 0.723 |
| Resting-state fMRI connectivity between Default Mode Network and Salience Network | NO₂tert | 0.410 | 0.913 |
| Resting-state fMRI connectivity between Default Mode Network and Ventral Attention Network | NO₂tert | 2.835 | 0.337 |
| Resting-state fMRI connectivity between Dorsal Attention Network and Dorsal Attention Network | NO₂tert | 2.459 | 0.337 |
| Resting-state fMRI connectivity between Dorsal Attention Network and Frontoparietal Network | NO₂tert | 8.483 | **0.008** |
| Resting-state fMRI connectivity between Dorsal Attention Network and Salience Network | NO₂tert | 2.541 | 0.337 |
| Resting-state fMRI connectivity between Dorsal Attention Network and Ventral Attention Network | NO₂tert | 0.604 | 0.910 |
| Resting-state fMRI connectivity between Frontoparietal Network and Frontoparietal Network | NO₂tert | 1.893 | 0.470 |
| Resting-state fMRI connectivity between Frontoparietal Network and Salience Network | NO₂tert | 1.544 | 0.552 |
| Resting-state fMRI connectivity between Frontoparietal Network and Ventral Attention Network | NO₂tert | 0.228 | 0.954 |
| Resting-state fMRI connectivity between Salience Network and Salience Network | NO₂tert | 0.666 | 0.910 |
| Resting-state fMRI connectivity between Salience Network and Visual Network | NO₂tert | 0.203 | 0.954 |
| Resting-state fMRI connectivity between Ventral Attention Network and Ventral Attention Network | NO₂tert | 2.345 | 0.337 |

**Table S.29. Results from linear mixed-effects models testing the effects of NO₂ tertile on resting-state functional connectivity (adjusted model).**

| **Resting-state fMRI connectivity** | **term** | **F** | **p-FDR** |
| --- | --- | --- | --- |
| Resting-state fMRI connectivity between Cingulate Gyrus and Cingulate Gyrus | NO₂tert | 0.419 | 0.961 |
| Resting-state fMRI connectivity between Cingulate Gyrus and CinguloParietal | NO₂tert | 1.908 | 0.422 |
| Resting-state fMRI connectivity between Cingulate Gyrus and Default Mode Network | NO₂tert | 3.331 | 0.178 |
| Resting-state fMRI connectivity between Cingulate Gyrus and Dorsal Attention Network | NO₂tert | 0.332 | 0.961 |
| Resting-state fMRI connectivity between Cingulate Gyrus and Frontoparietal Network | NO₂tert | 3.317 | 0.178 |
| Resting-state fMRI connectivity between Cingulate Gyrus and Salience Network | NO₂tert | 0.279 | 0.961 |
| Resting-state fMRI connectivity between Cingulate Gyrus and Ventral Attention Network | NO₂tert | 0.132 | 0.986 |
| Resting-state fMRI connectivity between CinguloParietal and CinguloParietal | NO₂tert | 0.027 | 0.986 |
| Resting-state fMRI connectivity between CinguloParietal and Default Mode Network | NO₂tert | 4.592 | 0.099 |
| Resting-state fMRI connectivity between CinguloParietal and Dorsal Attention Network | NO₂tert | 0.779 | 0.818 |
| Resting-state fMRI connectivity between CinguloParietal and Frontoparietal Network | NO₂tert | 0.062 | 0.986 |
| Resting-state fMRI connectivity between CinguloParietal and Salience Network | NO₂tert | 0.236 | 0.961 |
| Resting-state fMRI connectivity between CinguloParietal and Ventral Attention Network | NO₂tert | 0.702 | 0.818 |
| Resting-state fMRI connectivity between Default Mode Network and Default Mode Network | NO₂tert | 1.240 | 0.627 |
| Resting-state fMRI connectivity between Default Mode Network and Dorsal Attention Network | NO₂tert | 5.590 | 0.056 |
| Resting-state fMRI connectivity between Default Mode Network and Frontoparietal Network | NO₂tert | 1.748 | 0.450 |
| Resting-state fMRI connectivity between Default Mode Network and Salience Network | NO₂tert | 0.282 | 0.961 |
| Resting-state fMRI connectivity between Default Mode Network and Ventral Attention Network | NO₂tert | 1.141 | 0.645 |
| Resting-state fMRI connectivity between Dorsal Attention Network and Dorsal Attention Network | NO₂tert | 2.801 | 0.221 |
| Resting-state fMRI connectivity between Dorsal Attention Network and Frontoparietal Network | NO₂tert | 6.618 | **0.049** |
| Resting-state fMRI connectivity between Dorsal Attention Network and Salience Network | NO₂tert | 3.330 | 0.178 |
| Resting-state fMRI connectivity between Dorsal Attention Network and Ventral Attention Network | NO₂tert | 0.737 | 0.818 |
| Resting-state fMRI connectivity between Frontoparietal Network and Frontoparietal Network | NO₂tert | 2.608 | 0.256 |
| Resting-state fMRI connectivity between Frontoparietal Network and Salience Network | NO₂tert | 1.301 | 0.627 |
| Resting-state fMRI connectivity between Frontoparietal Network and Ventral Attention Network | NO₂tert | 0.014 | 0.986 |
| Resting-state fMRI connectivity between Salience Network and Salience Network | NO₂tert | 0.476 | 0.961 |
| Resting-state fMRI connectivity between Salience Network and Visual Network | NO₂tert | 0.075 | 0.986 |
| Resting-state fMRI connectivity between Ventral Attention Network and Ventral Attention Network | NO₂tert | 2.946 | 0.212 |

**Table S.30. Pairwise contrasts for the effects of NO₂ tertile on resting-state functional connectivity (adjusted model).**

| **Resting-state fMRI connectivity** | **Contrast** | **Estimate** | **SE** | **p-FDR** |
| --- | --- | --- | --- | --- |
| Resting-state fMRI connectivity between Default Mode Network and Dorsal Attention Network | NO₂ Low - NO₂ Medium | 0.006 | 0.004 | 0.185 |
|  | NO₂ Low - NO₂ High | 0.014 | 0.005 | **0.006** |
|  | NO₂ Medium - NO₂ High | 0.009 | 0.004 | 0.063 |
| Resting-state fMRI connectivity between Dorsal Attention Network and Frontoparietal Network | NO₂ Low - NO₂ Medium | 0.011 | 0.003 | **0.003** |
|  | NO₂ Low - NO₂ High | 0.010 | 0.004 | **0.012** |
|  | NO₂ Medium - NO₂ High | -0.002 | 0.003 | 0.597 |

**Table S.31. Results from linear mixed-effects models testing the effects of NO₂ tertile on resting-state functional connectivity including effects of population density and distance to road (adjusted model, sensitivity analysis).**

| **Resting-state fMRI connectivity** | **term** | **F** | **p-FDR** |
| --- | --- | --- | --- |
| Resting-state fMRI connectivity between Cingulate Gyrus and Cingulate Gyrus | NO₂tert | 0.363 | 0.917 |
| Resting-state fMRI connectivity between Cingulate Gyrus and CinguloParietal | NO₂tert | 1.623 | 0.508 |
| Resting-state fMRI connectivity between Cingulate Gyrus and Default Mode Network | NO₂tert | 3.354 | 0.164 |
| Resting-state fMRI connectivity between Cingulate Gyrus and Dorsal Attention Network | NO₂tert | 0.581 | 0.872 |
| Resting-state fMRI connectivity between Cingulate Gyrus and Frontoparietal Network | NO₂tert | 3.280 | 0.164 |
| Resting-state fMRI connectivity between Cingulate Gyrus and Salience Network | NO₂tert | 0.284 | 0.917 |
| Resting-state fMRI connectivity between Cingulate Gyrus and Ventral Attention Network | NO₂tert | 0.289 | 0.917 |
| Resting-state fMRI connectivity between CinguloParietal and CinguloParietal | NO₂tert | 0.064 | 0.952 |
| Resting-state fMRI connectivity between CinguloParietal and Default Mode Network | NO₂tert | 4.387 | 0.121 |
| Resting-state fMRI connectivity between CinguloParietal and Dorsal Attention Network | NO₂tert | 0.846 | 0.756 |
| Resting-state fMRI connectivity between CinguloParietal and Frontoparietal Network | NO₂tert | 0.049 | 0.952 |
| Resting-state fMRI connectivity between CinguloParietal and Salience Network | NO₂tert | 0.308 | 0.917 |
| Resting-state fMRI connectivity between CinguloParietal and Ventral Attention Network | NO₂tert | 0.843 | 0.756 |
| Resting-state fMRI connectivity between Default Mode Network and Default Mode Network | NO₂tert | 1.183 | 0.647 |
| Resting-state fMRI connectivity between Default Mode Network and Dorsal Attention Network | NO₂tert | 5.831 | **0.045** |
| Resting-state fMRI connectivity between Default Mode Network and Frontoparietal Network | NO₂tert | 1.650 | 0.508 |
| Resting-state fMRI connectivity between Default Mode Network and Salience Network | NO₂tert | 0.231 | 0.922 |
| Resting-state fMRI connectivity between Default Mode Network and Ventral Attention Network | NO₂tert | 1.139 | 0.647 |
| Resting-state fMRI connectivity between Dorsal Attention Network and Dorsal Attention Network | NO₂tert | 3.243 | 0.164 |
| Resting-state fMRI connectivity between Dorsal Attention Network and Frontoparietal Network | NO₂tert | 7.382 | **0.025** |
| Resting-state fMRI connectivity between Dorsal Attention Network and Salience Network | NO₂tert | 3.746 | 0.164 |
| Resting-state fMRI connectivity between Dorsal Attention Network and Ventral Attention Network | NO₂tert | 0.730 | 0.794 |
| Resting-state fMRI connectivity between Frontoparietal Network and Frontoparietal Network | NO₂tert | 2.957 | 0.192 |
| Resting-state fMRI connectivity between Frontoparietal Network and Salience Network | NO₂tert | 1.392 | 0.588 |
| Resting-state fMRI connectivity between Frontoparietal Network and Ventral Attention Network | NO₂tert | 0.064 | 0.952 |
| Resting-state fMRI connectivity between Salience Network and Salience Network | NO₂tert | 0.460 | 0.917 |
| Resting-state fMRI connectivity between Salience Network and Visual Network | NO₂tert | 0.195 | 0.922 |
| Resting-state fMRI connectivity between Ventral Attention Network and Ventral Attention Network | NO₂tert | 2.814 | 0.192 |

**Table S.32. Results from linear mixed-effects models testing the effects of NO₂ tertile on resting-state functional connectivity stratified according to income (fully adjusted model).**

| **Resting-state fMRI connectivity** | **term** | **F** | **p-FDR** |
| --- | --- | --- | --- |
| **Low income** | | | |
| Resting-state fMRI connectivity between Cingulate Gyrus and Cingulate Gyrus | NO₂tert | 0.764 | 0.775 |
| Resting-state fMRI connectivity between Cingulate Gyrus and CinguloParietal | NO₂tert | 0.228 | 0.900 |
| Resting-state fMRI connectivity between Cingulate Gyrus and Default Mode Network | NO₂tert | 5.203 | 0.092 |
| Resting-state fMRI connectivity between Cingulate Gyrus and Dorsal Attention Network | NO₂tert | 0.222 | 0.900 |
| Resting-state fMRI connectivity between Cingulate Gyrus and Frontoparietal Network | NO₂tert | 2.871 | 0.427 |
| Resting-state fMRI connectivity between Cingulate Gyrus and Salience Network | NO₂tert | 2.426 | 0.504 |
| Resting-state fMRI connectivity between Cingulate Gyrus and Ventral Attention Network | NO₂tert | 0.480 | 0.867 |
| Resting-state fMRI connectivity between CinguloParietal and CinguloParietal | NO₂tert | 0.432 | 0.868 |
| Resting-state fMRI connectivity between CinguloParietal and Default Mode Network | NO₂tert | 2.254 | 0.504 |
| Resting-state fMRI connectivity between CinguloParietal and Dorsal Attention Network | NO₂tert | 0.022 | 0.978 |
| Resting-state fMRI connectivity between CinguloParietal and Frontoparietal Network | NO₂tert | 0.142 | 0.900 |
| Resting-state fMRI connectivity between CinguloParietal and Salience Network | NO₂tert | 0.806 | 0.775 |
| Resting-state fMRI connectivity between CinguloParietal and Ventral Attention Network | NO₂tert | 0.356 | 0.893 |
| Resting-state fMRI connectivity between Default Mode Network and Default Mode Network | NO₂tert | 1.775 | 0.680 |
| Resting-state fMRI connectivity between Default Mode Network and Dorsal Attention Network | NO₂tert | 2.853 | 0.427 |
| Resting-state fMRI connectivity between Default Mode Network and Frontoparietal Network | NO₂tert | 1.060 | 0.775 |
| Resting-state fMRI connectivity between Default Mode Network and Salience Network | NO₂tert | 0.792 | 0.775 |
| Resting-state fMRI connectivity between Default Mode Network and Ventral Attention Network | NO₂tert | 1.031 | 0.775 |
| Resting-state fMRI connectivity between Dorsal Attention Network and Dorsal Attention Network | NO₂tert | 0.764 | 0.775 |
| Resting-state fMRI connectivity between Dorsal Attention Network and Frontoparietal Network | NO₂tert | 1.219 | 0.775 |
| Resting-state fMRI connectivity between Dorsal Attention Network and Salience Network | NO₂tert | 6.264 | 0.069 |
| Resting-state fMRI connectivity between Dorsal Attention Network and Ventral Attention Network | NO₂tert | 1.666 | 0.680 |
| Resting-state fMRI connectivity between Frontoparietal Network and Frontoparietal Network | NO₂tert | 0.151 | 0.900 |
| Resting-state fMRI connectivity between Frontoparietal Network and Salience Network | NO₂tert | 0.778 | 0.775 |
| Resting-state fMRI connectivity between Frontoparietal Network and Ventral Attention Network | NO₂tert | 0.167 | 0.900 |
| Resting-state fMRI connectivity between Salience Network and Salience Network | NO₂tert | 1.490 | 0.713 |
| Resting-state fMRI connectivity between Salience Network and Visual Network | NO₂tert | 0.560 | 0.843 |
| Resting-state fMRI connectivity between Ventral Attention Network and Ventral Attention Network | NO₂tert | 0.611 | 0.843 |
| **Middle income** | | | |
| Resting-state fMRI connectivity between Cingulate Gyrus and Cingulate Gyrus | NO₂tert | 0.812 | 0.832 |
| Resting-state fMRI connectivity between Cingulate Gyrus and CinguloParietal | NO₂tert | 0.500 | 0.971 |
| Resting-state fMRI connectivity between Cingulate Gyrus and Default Mode Network | NO₂tert | 1.176 | 0.727 |
| Resting-state fMRI connectivity between Cingulate Gyrus and Dorsal Attention Network | NO₂tert | 1.369 | 0.723 |
| Resting-state fMRI connectivity between Cingulate Gyrus and Frontoparietal Network | NO₂tert | 0.190 | 0.971 |
| Resting-state fMRI connectivity between Cingulate Gyrus and Salience Network | NO₂tert | 0.366 | 0.971 |
| Resting-state fMRI connectivity between Cingulate Gyrus and Ventral Attention Network | NO₂tert | 0.888 | 0.827 |
| Resting-state fMRI connectivity between CinguloParietal and CinguloParietal | NO₂tert | 0.248 | 0.971 |
| Resting-state fMRI connectivity between CinguloParietal and Default Mode Network | NO₂tert | 2.909 | 0.356 |
| Resting-state fMRI connectivity between CinguloParietal and Dorsal Attention Network | NO₂tert | 1.390 | 0.723 |
| Resting-state fMRI connectivity between CinguloParietal and Frontoparietal Network | NO₂tert | 0.326 | 0.971 |
| Resting-state fMRI connectivity between CinguloParietal and Salience Network | NO₂tert | 0.010 | 0.990 |
| Resting-state fMRI connectivity between CinguloParietal and Ventral Attention Network | NO₂tert | 3.547 | 0.321 |
| Resting-state fMRI connectivity between Default Mode Network and Default Mode Network | NO₂tert | 1.622 | 0.723 |
| Resting-state fMRI connectivity between Default Mode Network and Dorsal Attention Network | NO₂tert | 2.830 | 0.356 |
| Resting-state fMRI connectivity between Default Mode Network and Frontoparietal Network | NO₂tert | 0.256 | 0.971 |
| Resting-state fMRI connectivity between Default Mode Network and Salience Network | NO₂tert | 0.030 | 0.990 |
| Resting-state fMRI connectivity between Default Mode Network and Ventral Attention Network | NO₂tert | 0.091 | 0.990 |
| Resting-state fMRI connectivity between Dorsal Attention Network and Dorsal Attention Network | NO₂tert | 1.391 | 0.723 |
| Resting-state fMRI connectivity between Dorsal Attention Network and Frontoparietal Network | NO₂tert | 5.016 | 0.263 |
| Resting-state fMRI connectivity between Dorsal Attention Network and Salience Network | NO₂tert | 0.887 | 0.827 |
| Resting-state fMRI connectivity between Dorsal Attention Network and Ventral Attention Network | NO₂tert | 0.305 | 0.971 |
| Resting-state fMRI connectivity between Frontoparietal Network and Frontoparietal Network | NO₂tert | 3.681 | 0.321 |
| Resting-state fMRI connectivity between Frontoparietal Network and Salience Network | NO₂tert | 0.183 | 0.971 |
| Resting-state fMRI connectivity between Frontoparietal Network and Ventral Attention Network | NO₂tert | 1.193 | 0.727 |
| Resting-state fMRI connectivity between Salience Network and Salience Network | NO₂tert | 0.042 | 0.990 |
| Resting-state fMRI connectivity between Salience Network and Visual Network | NO₂tert | 0.551 | 0.971 |
| Resting-state fMRI connectivity between Ventral Attention Network and Ventral Attention Network | NO₂tert | 2.044 | 0.611 |
| **High income** | | | |
| Resting-state fMRI connectivity between Cingulate Gyrus and Cingulate Gyrus | NO₂tert | 0.546 | 0.812 |
| Resting-state fMRI connectivity between Cingulate Gyrus and CinguloParietal | NO₂tert | 0.821 | 0.812 |
| Resting-state fMRI connectivity between Cingulate Gyrus and Default Mode Network | NO₂tert | 0.822 | 0.812 |
| Resting-state fMRI connectivity between Cingulate Gyrus and Dorsal Attention Network | NO₂tert | 2.095 | 0.650 |
| Resting-state fMRI connectivity between Cingulate Gyrus and Frontoparietal Network | NO₂tert | 5.558 | 0.123 |
| Resting-state fMRI connectivity between Cingulate Gyrus and Salience Network | NO₂tert | 0.028 | 0.983 |
| Resting-state fMRI connectivity between Cingulate Gyrus and Ventral Attention Network | NO₂tert | 1.566 | 0.671 |
| Resting-state fMRI connectivity between CinguloParietal and CinguloParietal | NO₂tert | 0.408 | 0.887 |
| Resting-state fMRI connectivity between CinguloParietal and Default Mode Network | NO₂tert | 1.823 | 0.650 |
| Resting-state fMRI connectivity between CinguloParietal and Dorsal Attention Network | NO₂tert | 0.150 | 0.975 |
| Resting-state fMRI connectivity between CinguloParietal and Frontoparietal Network | NO₂tert | 0.581 | 0.812 |
| Resting-state fMRI connectivity between CinguloParietal and Salience Network | NO₂tert | 0.611 | 0.812 |
| Resting-state fMRI connectivity between CinguloParietal and Ventral Attention Network | NO₂tert | 0.099 | 0.975 |
| Resting-state fMRI connectivity between Default Mode Network and Default Mode Network | NO₂tert | 0.017 | 0.983 |
| Resting-state fMRI connectivity between Default Mode Network and Dorsal Attention Network | NO₂tert | 1.703 | 0.650 |
| Resting-state fMRI connectivity between Default Mode Network and Frontoparietal Network | NO₂tert | 2.314 | 0.650 |
| Resting-state fMRI connectivity between Default Mode Network and Salience Network | NO₂tert | 0.545 | 0.812 |
| Resting-state fMRI connectivity between Default Mode Network and Ventral Attention Network | NO₂tert | 3.623 | 0.283 |
| Resting-state fMRI connectivity between Dorsal Attention Network and Dorsal Attention Network | NO₂tert | 0.866 | 0.812 |
| Resting-state fMRI connectivity between Dorsal Attention Network and Frontoparietal Network | NO₂tert | 4.001 | 0.280 |
| Resting-state fMRI connectivity between Dorsal Attention Network and Salience Network | NO₂tert | 1.870 | 0.650 |
| Resting-state fMRI connectivity between Dorsal Attention Network and Ventral Attention Network | NO₂tert | 0.126 | 0.975 |
| Resting-state fMRI connectivity between Frontoparietal Network and Frontoparietal Network | NO₂tert | 1.072 | 0.812 |
| Resting-state fMRI connectivity between Frontoparietal Network and Salience Network | NO₂tert | 1.071 | 0.812 |
| Resting-state fMRI connectivity between Frontoparietal Network and Ventral Attention Network | NO₂tert | 0.733 | 0.812 |
| Resting-state fMRI connectivity between Salience Network and Salience Network | NO₂tert | 0.239 | 0.975 |
| Resting-state fMRI connectivity between Salience Network and Visual Network | NO₂tert | 0.172 | 0.975 |
| Resting-state fMRI connectivity between Ventral Attention Network and Ventral Attention Network | NO₂tert | 0.810 | 0.812 |

**Table S.33. Results from linear mixed-effects models examining resting-state functional connectivity in relation to structural brain measures (fully adjusted model).**

| **Resting-state fMRI connectivity** | **term** | **F** | **p-FDR** |
| --- | --- | --- | --- |
| Resting-state fMRI connectivity between Cingulate Gyrus and Cingulate Gyrus | Right frontal pole area | 1.331 | 0.410 |
| Resting-state fMRI connectivity between Cingulate Gyrus and CinguloParietal | Right frontal pole area | 0.269 | 0.774 |
| Resting-state fMRI connectivity between Cingulate Gyrus and Default Mode Network | Right frontal pole area | 2.501 | 0.248 |
| Resting-state fMRI connectivity between Cingulate Gyrus and Dorsal Attention Network | Right frontal pole area | 8.203 | 0.024 |
| Resting-state fMRI connectivity between Cingulate Gyrus and Frontoparietal Network | Right frontal pole area | 1.999 | 0.294 |
| Resting-state fMRI connectivity between Cingulate Gyrus and Salience Network | Right frontal pole area | 3.708 | 0.218 |
| Resting-state fMRI connectivity between Cingulate Gyrus and Ventral Attention Network | Right frontal pole area | 3.705 | 0.218 |
| Resting-state fMRI connectivity between CinguloParietal and CinguloParietal | Right frontal pole area | 0.263 | 0.774 |
| Resting-state fMRI connectivity between CinguloParietal and Default Mode Network | Right frontal pole area | 0.440 | 0.710 |
| Resting-state fMRI connectivity between CinguloParietal and Dorsal Attention Network | Right frontal pole area | 0.000 | 0.985 |
| Resting-state fMRI connectivity between CinguloParietal and Frontoparietal Network | Right frontal pole area | 2.536 | 0.248 |
| Resting-state fMRI connectivity between CinguloParietal and Salience Network | Right frontal pole area | 0.030 | 0.916 |
| Resting-state fMRI connectivity between CinguloParietal and Ventral Attention Network | Right frontal pole area | 0.107 | 0.867 |
| Resting-state fMRI connectivity between Default Mode Network and Default Mode Network | Right frontal pole area | 8.999 | 0.024 |
| Resting-state fMRI connectivity between Default Mode Network and Dorsal Attention Network | Right frontal pole area | 2.448 | 0.248 |
| Resting-state fMRI connectivity between Default Mode Network and Frontoparietal Network | Right frontal pole area | 3.094 | 0.228 |
| Resting-state fMRI connectivity between Default Mode Network and Salience Network | Right frontal pole area | 0.183 | 0.814 |
| Resting-state fMRI connectivity between Default Mode Network and Ventral Attention Network | Right frontal pole area | 10.375 | 0.018 |
| Resting-state fMRI connectivity between Dorsal Attention Network and Dorsal Attention Network | Right frontal pole area | 12.321 | 0.013 |
| Resting-state fMRI connectivity between Dorsal Attention Network and Frontoparietal Network | Right frontal pole area | 3.037 | 0.228 |
| Resting-state fMRI connectivity between Dorsal Attention Network and Salience Network | Right frontal pole area | 3.480 | 0.218 |
| Resting-state fMRI connectivity between Dorsal Attention Network and Ventral Attention Network | Right frontal pole area | 8.408 | 0.024 |
| Resting-state fMRI connectivity between Frontoparietal Network and Frontoparietal Network | Right frontal pole area | 1.073 | 0.467 |
| Resting-state fMRI connectivity between Frontoparietal Network and Salience Network | Right frontal pole area | 1.442 | 0.403 |
| Resting-state fMRI connectivity between Frontoparietal Network and Ventral Attention Network | Right frontal pole area | 0.734 | 0.577 |
| Resting-state fMRI connectivity between Salience Network and Salience Network | Right frontal pole area | 0.044 | 0.916 |
| Resting-state fMRI connectivity between Salience Network and Visual Network | Right frontal pole area | 0.022 | 0.916 |
| Resting-state fMRI connectivity between Ventral Attention Network and Ventral Attention Network | Right frontal pole area | 2.369 | 0.248 |
| Resting-state fMRI connectivity between Cingulate Gyrus and Cingulate Gyrus | Left caudate volume | 0.672 | 0.999 |
| Resting-state fMRI connectivity between Cingulate Gyrus and CinguloParietal | Left caudate volume | 0.416 | 0.999 |
| Resting-state fMRI connectivity between Cingulate Gyrus and Default Mode Network | Left caudate volume | 0.249 | 0.999 |
| Resting-state fMRI connectivity between Cingulate Gyrus and Dorsal Attention Network | Left caudate volume | 0.204 | 0.999 |
| Resting-state fMRI connectivity between Cingulate Gyrus and Frontoparietal Network | Left caudate volume | 3.659 | 0.725 |
| Resting-state fMRI connectivity between Cingulate Gyrus and Salience Network | Left caudate volume | 0.027 | 0.999 |
| Resting-state fMRI connectivity between Cingulate Gyrus and Ventral Attention Network | Left caudate volume | 0.138 | 0.999 |
| Resting-state fMRI connectivity between CinguloParietal and CinguloParietal | Left caudate volume | 0.058 | 0.999 |
| Resting-state fMRI connectivity between CinguloParietal and Default Mode Network | Left caudate volume | 0.001 | 0.999 |
| Resting-state fMRI connectivity between CinguloParietal and Dorsal Attention Network | Left caudate volume | 0.000 | 0.999 |
| Resting-state fMRI connectivity between CinguloParietal and Frontoparietal Network | Left caudate volume | 0.525 | 0.999 |
| Resting-state fMRI connectivity between CinguloParietal and Salience Network | Left caudate volume | 0.009 | 0.999 |
| Resting-state fMRI connectivity between CinguloParietal and Ventral Attention Network | Left caudate volume | 2.673 | 0.816 |
| Resting-state fMRI connectivity between Default Mode Network and Default Mode Network | Left caudate volume | 3.010 | 0.774 |
| Resting-state fMRI connectivity between Default Mode Network and Dorsal Attention Network | Left caudate volume | 0.430 | 0.999 |
| Resting-state fMRI connectivity between Default Mode Network and Frontoparietal Network | Left caudate volume | 0.091 | 0.999 |
| Resting-state fMRI connectivity between Default Mode Network and Salience Network | Left caudate volume | 0.289 | 0.999 |
| Resting-state fMRI connectivity between Default Mode Network and Ventral Attention Network | Left caudate volume | 1.693 | 0.999 |
| Resting-state fMRI connectivity between Dorsal Attention Network and Dorsal Attention Network | Left caudate volume | 0.511 | 0.999 |
| Resting-state fMRI connectivity between Dorsal Attention Network and Frontoparietal Network | Left caudate volume | 0.007 | 0.999 |
| Resting-state fMRI connectivity between Dorsal Attention Network and Salience Network | Left caudate volume | 0.738 | 0.999 |
| Resting-state fMRI connectivity between Dorsal Attention Network and Ventral Attention Network | Left caudate volume | 0.011 | 0.999 |
| Resting-state fMRI connectivity between Frontoparietal Network and Frontoparietal Network | Left caudate volume | 0.585 | 0.999 |
| Resting-state fMRI connectivity between Frontoparietal Network and Salience Network | Left caudate volume | 4.395 | 0.676 |
| Resting-state fMRI connectivity between Frontoparietal Network and Ventral Attention Network | Left caudate volume | 0.668 | 0.999 |
| Resting-state fMRI connectivity between Salience Network and Salience Network | Left caudate volume | 0.641 | 0.999 |
| Resting-state fMRI connectivity between Salience Network and Visual Network | Left caudate volume | 0.024 | 0.999 |
| Resting-state fMRI connectivity between Ventral Attention Network and Ventral Attention Network | Left caudate volume | 1.056 | 0.999 |
| Resting-state fMRI connectivity between Cingulate Gyrus and Cingulate Gyrus | Right caudate volume | 1.277 | 0.999 |
| Resting-state fMRI connectivity between Cingulate Gyrus and CinguloParietal | Right caudate volume | 0.208 | 0.999 |
| Resting-state fMRI connectivity between Cingulate Gyrus and Default Mode Network | Right caudate volume | 0.012 | 0.999 |
| Resting-state fMRI connectivity between Cingulate Gyrus and Dorsal Attention Network | Right caudate volume | 0.769 | 0.999 |
| Resting-state fMRI connectivity between Cingulate Gyrus and Frontoparietal Network | Right caudate volume | 6.022 | 0.676 |
| Resting-state fMRI connectivity between Cingulate Gyrus and Salience Network | Right caudate volume | 1.640 | 0.999 |
| Resting-state fMRI connectivity between Cingulate Gyrus and Ventral Attention Network | Right caudate volume | 0.001 | 0.999 |
| Resting-state fMRI connectivity between CinguloParietal and CinguloParietal | Right caudate volume | 0.026 | 0.999 |
| Resting-state fMRI connectivity between CinguloParietal and Default Mode Network | Right caudate volume | 0.001 | 0.999 |
| Resting-state fMRI connectivity between CinguloParietal and Dorsal Attention Network | Right caudate volume | 0.075 | 0.999 |
| Resting-state fMRI connectivity between CinguloParietal and Frontoparietal Network | Right caudate volume | 0.009 | 0.999 |
| Resting-state fMRI connectivity between CinguloParietal and Salience Network | Right caudate volume | 0.030 | 0.999 |
| Resting-state fMRI connectivity between CinguloParietal and Ventral Attention Network | Right caudate volume | 1.116 | 0.999 |
| Resting-state fMRI connectivity between Default Mode Network and Default Mode Network | Right caudate volume | 1.826 | 0.999 |
| Resting-state fMRI connectivity between Default Mode Network and Dorsal Attention Network | Right caudate volume | 0.110 | 0.999 |
| Resting-state fMRI connectivity between Default Mode Network and Frontoparietal Network | Right caudate volume | 0.262 | 0.999 |
| Resting-state fMRI connectivity between Default Mode Network and Salience Network | Right caudate volume | 0.213 | 0.999 |
| Resting-state fMRI connectivity between Default Mode Network and Ventral Attention Network | Right caudate volume | 1.396 | 0.999 |
| Resting-state fMRI connectivity between Dorsal Attention Network and Dorsal Attention Network | Right caudate volume | 0.708 | 0.999 |
| Resting-state fMRI connectivity between Dorsal Attention Network and Frontoparietal Network | Right caudate volume | 0.000 | 0.999 |
| Resting-state fMRI connectivity between Dorsal Attention Network and Salience Network | Right caudate volume | 3.417 | 0.725 |
| Resting-state fMRI connectivity between Dorsal Attention Network and Ventral Attention Network | Right caudate volume | 0.124 | 0.999 |
| Resting-state fMRI connectivity between Frontoparietal Network and Frontoparietal Network | Right caudate volume | 0.656 | 0.999 |
| Resting-state fMRI connectivity between Frontoparietal Network and Salience Network | Right caudate volume | 4.901 | 0.676 |
| Resting-state fMRI connectivity between Frontoparietal Network and Ventral Attention Network | Right caudate volume | 2.466 | 0.816 |
| Resting-state fMRI connectivity between Salience Network and Salience Network | Right caudate volume | 0.250 | 0.999 |
| Resting-state fMRI connectivity between Salience Network and Visual Network | Right caudate volume | 0.038 | 0.999 |
| Resting-state fMRI connectivity between Ventral Attention Network and Ventral Attention Network | Right caudate volume | 0.871 | 0.999 |

**Table S.34. Results from linear mixed-effects models testing the association of air pollution, genetic risk for schizophrenia, and brain measures with mental health**

| **Measures** | **F** | **p-value** |
| --- | --- | --- |
| **Internalising symptoms** | | |
| PM₂.₅ | 0.198 | 0.819 |
| NO₂ | 1.067 | 0.346 |
| O₃ | 2.097 | 0.124 |
| PRS-SCZ | 2.619 | 0.073 |
| Cortical area in mm² of right frontal pole | 0.469 | 0.493 |
| Volume in mm³ of left-caudate | 0.243 | 0.621 |
| Volume in mm³ of right-caudate | 0.013 | 0.906 |
| Resting-state fMRI connectivity between Default Mode Network and Dorsal Attention Network | 6.450 | **0.011** |
| Resting-state fMRI connectivity between Dorsal Attention Network and Frontoparietal Network | 0.605 | 0.436 |
| **Externalising symptoms** | | |
| PM₂.₅ | 1.449 | 0.235 |
| NO₂ | 1.635 | 0.199 |
| O₃ | 0.900 | 0.408 |
| PRS-SCZ | 0.983 | 0.374 |
| Cortical area in mm² of right frontal pole | 0.228 | 0.956 |
| Volume in mm³ of left-caudate | 3.046 | 0.081 |
| Volume in mm³ of right-caudate | 2.092 | 0.148 |
| Resting-state fMRI connectivity between Default Mode Network and Dorsal Attention Network | 4.903 | **0.026** |
| Resting-state fMRI connectivity between Dorsal Attention Network and Frontoparietal Network | 0.813 | 0.367 |
| **Psychotic like experiences** | | |
| PM₂.₅ | 3.206 | **0.046** |
| NO₂ | 0.194 | 0.823 |
| O₃ | 0.327 | 0.720 |
| PRS-SCZ | 2.613 | 0.106 |
| Cortical area in mm² of right frontal pole | 0.620 | 0.912 |
| Volume in mm³ of left-caudate | 0.652 | 0.419 |
| Volume in mm³ of right-caudate | 0.262 | 0.608 |
| Resting-state fMRI connectivity between Default Mode Network and Dorsal Attention Network | 0.824 | 0.363 |
| Resting-state fMRI connectivity between Dorsal Attention Network and Frontoparietal Network | 0.015 | 0.901 |

**Figure S.1. Construction of the analytic sample**

50 excluded due to poor quality imaging

1,404 excluded due to missing values on PRS-SCZ or air pollution exposure

1,580 analyzed

8,834 excluded due to missing imaging data at year 4

11,868 enrolled

**Figure S.2. Association between NO₂ exposure and resting-state functional connectivity in Default Mode Network-Dorsal Attention Network and Dorsal Attention Network*–* Frontoparietal Network (fully adjusted model).** Estimated differences in resting-state functional connectivity across tertiles of NO₂ exposure. The left side shows the connectivity between the Default Mode Network (DMN) and Dorsal Attention Network (DAN), while the right side shows the connectivity between the Dorsal Attention Network (DAN) and Frontoparietal Network (FPN). Points represent estimated contrasts, with 95% confidence intervals shown as error bars. Statistical significance is indicated as follows: ** p ≤ 0.01, * p ≤ 0.05. Graphs were generated using **ggplot2** in R.

**Figure S.3**. **Association between NO₂ exposure and resting-state functional connectivity in Default Mode Network-Dorsal Attention Network and Dorsal Attention Network*–* Frontoparietal Network (adjusted model).** Estimated differences in resting-state functional connectivity across tertiles of NO₂ exposure. Left side shows the connectivity between the Default Mode Network (DMN) and Dorsal Attention Network (DLA), while right side shows the connectivity between the Dorsal Attention Network (DLA) and Frontoparietal Network (FPN). Points represent estimated contrasts, with 95% confidence intervals shown as error bars. Statistical significance is indicated as follows: ** p ≤ 0.01, * p ≤ 0.05, ns = not significant. Graphs were generated using **ggplot2** in R.

**Figure S.4. Association between PM₂.₅ exposure and right frontal pole area (adjusted model)**. Differences in right frontal pole cortical area (mm²) across PM₂.₅ exposure tertiles. Points represent estimated differences between tertiles, with 95% confidence intervals shown as error bars. Statistical significance is indicated as follows: ** p ≤ 0.01, * p ≤ 0.05, ns = not significant. Graphs were generated using **ggplot2** in R.

**References**

1.	Casey BJ, Cannonier T, Conley MI, et al. The Adolescent Brain Cognitive Development (ABCD) study: Imaging acquisition across 21 sites. *Dev Cogn Neurosci*. 2018;32:43-54. doi:10.1016/J.DCN.2018.03.001

2.	Hagler DJ, Hatton SN, Cornejo MD, et al. Image processing and analysis methods for the Adolescent Brain Cognitive Development Study. *Neuroimage*. 2019;202:116091. doi:10.1016/J.NEUROIMAGE.2019.116091

3.	White N, Roddey C, Shankaranarayanan A, et al. PROMO: Real-time prospective motion correction in MRI using image-based tracking. *Magn Reson Med*. 2010;63(1):91-105. doi:10.1002/MRM.22176

4.	Tisdall MD, Hess AT, Reuter M, Meintjes EM, Fischl B, Van Der Kouwe AJW. Volumetric navigators for prospective motion correction and selective reacquisition in neuroanatomical MRI. *Magn Reson Med*. 2012;68(2):389-399. doi:10.1002/MRM.23228

5.	Dale AM, Fischl B, Sereno MI. Cortical Surface-Based Analysis: I. Segmentation and Surface Reconstruction. *Neuroimage*. 1999;9(2):179-194. doi:10.1006/NIMG.1998.0395

6.	Calderón-Garcidueñas L, Calderón-Garcidueñas A, Torres-Jardón R, Avila-Ramírez J, Kulesza RJ, Angiulli AD. Air pollution and your brain: what do you need to know right now. *Prim Health Care Res Dev*. 2015;16(4):329-345. doi:10.1017/S146342361400036X

7.	Herting MM, Younan D, Campbell CE, Chen JC. Outdoor Air Pollution and Brain Structure and Function From Across Childhood to Young Adulthood: A Methodological Review of Brain MRI Studies. *Front Public Health*. 2019;7:466667. doi:10.3389/FPUBH.2019.00332/XML

8.	Cserbik D, Chen JC, McConnell R, et al. Fine particulate matter exposure during childhood relates to hemispheric-specific differences in brain structure. *Environ Int*. 2020;143:105933. doi:10.1016/J.ENVINT.2020.105933

9.	Zundel CG, Ryan P, Brokamp C, et al. Air pollution, depressive and anxiety disorders, and brain effects: A systematic review. *Neurotoxicology*. *Elsevier B.V.* 2022;93:272-300. doi:10.1016/j.neuro.2022.10.011

10.	Gordon EM, Laumann TO, Adeyemo B, Huckins JF, Kelley WM, Petersen SE. Generation and Evaluation of a Cortical Area Parcellation from Resting-State Correlations. *Cerebral Cortex*. 2016;26(1):288-303. doi:10.1093/CERCOR/BHU239

11.	Cotter DL, Campbell CE, Sukumaran K, et al. Effects of ambient fine particulates, nitrogen dioxide, and ozone on maturation of functional brain networks across early adolescence. *Environ Int*. 2023;177:108001. doi:10.1016/J.ENVINT.2023.108001

12.	Fan CC, Marshall A, Smolker H, et al. Adolescent Brain Cognitive Development (ABCD) study Linked External Data (LED): Protocol and practices for geocoding and assignment of environmental data. *Dev Cogn Neurosci*. 2021;52:101030. doi:10.1016/J.DCN.2021.101030

13.	Di Q, Amini H, Shi L, et al. An ensemble-based model of PM2.5 concentration across the contiguous United States with high spatiotemporal resolution. *Environ Int*. 2019;130:104909. doi:10.1016/J.ENVINT.2019.104909

14.	Di Q, Amini H, Shi L, et al. Assessing NO2 Concentration and Model Uncertainty with High Spatiotemporal Resolution across the Contiguous United States Using Ensemble Model Averaging. *Environ Sci Technol*. 2020;54(3):1372-1384. doi:10.1021/ACS.EST.9B03358
